# New Pregnane Glycosides from *Mandevilla dardanoi* and Their Anti-Inflammatory Activity

**DOI:** 10.3390/molecules27185992

**Published:** 2022-09-14

**Authors:** Francisca S. V. Lins, Thalisson A. de Souza, Luiza C. F. Opretzka, Joanda P. R. e Silva, Laiane C. O. Pereira, Lucas S. Abreu, Anderson A. V. Pinheiro, George L. D. dos Santos, Yuri M. do Nascimento, José Iranildo Miranda de Melo, Raimundo Braz-Filho, Cristiane F. Villarreal, Marcelo S. da Silva, Josean F. Tavares

**Affiliations:** 1 Postgraduate Program in Natural and Synthetic Bioactive Products, Federal University of Paraíba, João Pessoa 58051-90, Paraíba, Brazil; 2 School of Pharmacy, Federal University of Bahia, Salvador 40170-115, Bahia, Brazil; 3 Department of Chemistry, Institute of Chemistry, Fluminense Federal University, Niterói 24020-150, Rio de Janeiro, Brazil; 4 Department of Biology, Centre of Biological Sciences and Health, State University of Paraíba, Campina Grande 58429-500, Paraíba, Brazil; 5 Department of Chemistry, Institute of Chemistry, Federal Rural University of Rio de Janeiro, Seropédica 23851-970, Rio de Janeiro, Brazil

**Keywords:** *Mandevilla dardanoi*, Apocynaceae, pregnane glycosides, anti-inflammatory

## Abstract

*Mandevilla* Lindl. is an important genus of the Apocynaceae family, not only as ornamental plants but also for its medicinal uses. In Brazil, *Mandevilla* species are indicated to treat asthma and skin infections, their anti-inflammatory potential and wound healing properties are also reported in the literature. Concerning their chemical composition, this group of plants is a conspicuous producer of pregnane glycosides. *Mandevilla dardanoi* is an endemic species from the Brazilian semiarid region not studied by any phytochemical methods. In view of the medicinal potential of *Mandevilla* species, this study aimed to isolate new pregnane glycosides from *M. dardanoi*. To achieve this main goal, modern chromatography techniques were employed. Five new pregnane glycosides, dardanols A-E, were isolated from the roots of *M. dardanoi* by HPLC. Their structures were determined using extensive 1D and 2D-NMR and mass spectrometry (MS^n^ and HRESIMS) data. The cytotoxicity and the anti-inflammatory potential of these compounds were evaluated. The first was evaluated by measuring proinflammatory cytokines and nitric oxide production by stimulated macrophages. Dardanols were able to inhibit the production of nitric oxide and reduce IL-1β and TNF-α. The current work demonstrates the chemodiversity of Brazilian semiarid species and contributes to amplifying knowledge about the biological potential of the *Mandevilla* genus.

## 1. Introduction

*Mandevilla* Lindl. is one of the largest neotropical genera of the Apocynaceae family, this group comprises approximately 170 species [1,2,3]. First registered in 2017, *M. dardanoi* M.F. Sales, Kin.-Gouv. & A.O. Simões is an endemic species from the Brazilian semiarid region, occurring in the states of Pernambuco and Paraíba [4], there are no studies regarding their chemical composition or pharmacological activities [4]. Rich in flavonoids, steroids, and pregnane glycosides *Mandevilla* species have been used by folk medicine in therapy for snakebites, wound healing, and to treat skin infections and inflammation [5,6,7,8,9,10].

Composed of a steroidal scaffold, the pregnane (C_21_) and seco-pregnane-type glycosides mainly occur in Apocynaceae, Malpighiaceae, Ranunculaceae, and Zygophyllaceae families [11]. This class of compounds has demonstrated remarkable biological activities, including anticancer, antinociceptive, anti-inflammatory, antiviral, and antibacterial properties [12,13,14,15,16]. Considering the medicinal potential of *Mandevilla* species, the current study aimed to isolate novel bioactive pregnane derivatives from the roots of *M. dardanoi*. Five new pregnane glycosides, dardanols A–E, were isolated and characterized by spectroscopic and spectrometric analysis. The cytotoxicity and anti-inflammatory activity of dardanols A, B, C, and E were also assessed. All of the compounds tested were active in the models applied.

## 2. Results and Discussion

### 2.1. Structural Assignment

Compound **1** was isolated as a white powder with a positive optical rotation of [*α*]^25^_D_ +14 (c 0.1, pyridine). Its molecular formula was determined to be C_57_H_90_O_20_ by the sodium adduct HRESIMS ion at *m*/*z* 1117.5921 [M + Na]^+^ (calcd. for C_57_H_90_NaO_20_, 1117,5917, Δ = 0.3 ppm), indicating 13 indices of hydrogen deficiency (IHD) (Figure S1).

The ^1^H and ^13^C NMR data (Table 1 and Table 2 and supporting information) showed characteristic signals (chemical shifts given in ppm) of pregnane glycosides already isolated from the *Mandevilla* genus. The hydrogen signals at *δ*_H_ 5.75 (d, *J =* 2.5 Hz, H-16) corresponding to an acetal group hydrogen, *δ*_H_ 5.41 (m, H-6), an olefinic hydrogen, *δ*_H_ 4.58 (t, *J =* 2.4 Hz, H-20) and 3.81 (m, H-3), oxymethinic hydrogens, 1.06 (s, CH_3_-18) and 0. 94 (s, CH_3_-19), methyl hydrogens, associated with their respective carbons at *δ*_C_ 104.2 (C-16), 122.1 (C-6), 78.9 (C-20), 77.5 (C-3), 16.9 (C-18) and 19.7 (C-19), are in agreement with the values described for the aglycone illustrol, a seco-norpregnane derivative, isolated from *M. illustris* and reported only once [17]. The five anomeric hydrogen signals at 4.76 (dd, *J =* 9.5, 2.0 Hz, H-1′), 5.47 (dd, *J =* 4.4, 2.2 Hz, H-1″), *δ*_H_ 5.15 (dd, *J =* 9.6, 1.6 Hz, H-1‴), *δ*_H_ 5.08 (dd, *J =* 9.6, 1.7 Hz, H-1⁗) and 4.71 (dd, *J =* 9.7, 2.0 Hz, H′′′′′), as well as the presence of the methyl hydrogen signals at 1.49 (3H, d, *J =* 6.4 Hz, C-6′), 1.33 (6H, d, *J =* 6.2 Hz, C-6″ and C-6‴), 1.39 (3H, d, *J =* 6.2 Hz, C-6⁗) and 1.29 (3H, d, *J =* 6.4 Hz, C-6′′′′′), and the hydrogens of the methoxy groups at *δ*_H_ 3.33 (s, OCH_3_′), *δ*_H_ 3. 39 (s, OCH_3_″), *δ*_H_ 3.60 (s, OCH_3_‴), *δ*_H_ 3.46 (s, OCH_3_⁗), and *δ*_H_ 3.38 (s, OCH_3_′′′′′) indicated the existence of five osidic units in the structure of 1. This evidence was corroborated by the presence of the signals in the ^13^C NMR spectrum corresponding to five anomeric carbons at *δ*_C_ 102. 9, 100.9, 100.8, 99.0, and 97.3, five methyl carbons at *δ*_C_ 19.0, *δ*_C_ 18.9, *δ*_C_ 18.4, *δ*_C_ 17.5, and *δ*_C_ 17.2, and five methoxy carbons at *δ*_C_ 59.3, *δ*_C_ 58.9, *δ*_C_ 56.8, *δ*_C_ 56.7, and *δ*_C_ 56.5.

In the HSQC spectrum, the anomeric hydrogen at *δ*_H_ 4.76 (dd, *J =* 9.5, 2.0 Hz) correlated with the carbon signal at *δ*_C_ 99.0. In the HMBC spectrum, this same hydrogen showed a long-distance correlation with the carbon at *δ*_C_ 77.6 (C-3), thus being assigned to H-1′. Conversely, the hydrogen signal at *δ*_H_ 3.81 (H-3) correlated with the carbon at *δ*_C_ 99.0, confirming the bonding of the first glycosyl unit to the aglycone portion. Furthermore, this assignment was corroborated through cross-correlations of *δ*_H_ 3.81 (H-3) and *δ*_H_ 5.41 (H-6) with *δ*_C_ 39.6 (C-4). In the COSY spectrum, a correlation of the signal at *δ*_H_ 4.76 (H-1) with *δ*_H_ 2.22 (H-2′) and of that with *δ*_H_ 3.41(H-3′) was observed. In the HSQC spectrum, H-2′ and H-3′ correlated with the carbons at *δ*_C_ 28.7 (C-2′) and *δ*_C_ 81.3, respectively. These data compared with the literature allowed us to assign the osidic unit I as being diginopyransideo [18]. The H-1′ coupling constants at 9.7 and 2.0 Hz are compatible with β-glycosidic bonding. According to the literature, a chemical shift of C-2 of the osidic units smaller than 34 ppm corresponds to an L configuration and larger than 35 ppm to a D configuration [19]. This effect is associated with the orientation of the ether group in the axial present in C-4, which causes a gamma shielding effect at C-2′. Thus, with the chemical shift of C-2′ being equal to *δ*_C_ 28.7, it was possible to assign the configuration of the osidic unit I as β-L-diginopyranosyl.

In the HSQC spectrum, the anomeric hydrogen at *δ*_H_ 5.47 (dd, *J =* 4.4 and 2.2 Hz) correlates with the carbon at *δ*_C_ 97.3. In the HMBC spectrum, the same signal correlated with the carbon at *δ*_C_ 71.0 (C-4′) was assigned to H-1″, confirming the union of the sugars through 1–4 bonds. In the COSY spectrum, a correlation of the hydrogen H-1″ was observed with the signal at *δ*_H_ 2.18 and of this with the signal at *δ*_H_ 3.96, which were assigned to H-2″ and H-3″. In the HSQC spectrum, H-2″ and H-3″ correlate with the carbons at *δ*_C_ 33.8 and *δ*_C_ 76.3, respectively. Thus, by comparing the other data that are presented in Table 1 with the data present in the literature, it is possible to assign the osidic unit II as being the sarmentopyranoside [11]. The coupling constants at 4.4 and 2.2 Hz are compatible with α-glycosidic bonding and the C-2″ value at *δ*_C_ 33.8 with the L configuration for acidic unit II, which was assigned as β-L-sarmentopyranosyl. 

In the HSQC spectrum, the anomeric hydrogen at *δ*_H_ 5.15 (dd, *J =* 9.6 and 1.6, H-1‴) correlated with the carbon at *δ*_C_ 100.8. In the HMBC spectrum, a correlation of the signal at *δ*_H_ 3.73 (H-4″) with the carbon at *δ*_C_ 100.8 was observed, confirming the binding of osidic unit II to III via 1–4 bonds. Similar to assignments performed previously, the COSY and HSQC spectra were analyzed together to perform the ^1^H and ^13^C assignments of osidic unit III (Table 1 and Table 2). By comparing these data with the literature, it is possible to assign osidic unit III as a cymaropyranoside [11]. The coupling constants at 9.6 and 1.6 Hz are compatible with β-glycosidic bonding, and the chemical shift value of C-2‴ at 37.0 allowed us to assign the configuration of osidic unit III as β-D-cymaropyranosyl.

For osidic unit IV, the signal of the anomeric hydrogen at *δ*_H_ 5.08 (dd, *J =* 9.6 and 1.7 Hz, H-1⁗) was observed to correlate in the HSQC spectrum with the carbon at *δ*_C_ 100.9. In the HMBC spectrum, a correlation of the signal at *δ*_H_ 3.48 (H-4‴) with the carbon at *δ*_C_ 100.9 was observed, which was assigned to C-1⁗, confirming the union of osidic units III and IV. In the COSY spectrum, a correlation of *δ*_H_ 5.08 (H-1⁗) with *δ*_H_ 2.35 (H-2⁗) and of this with *δ*_H_ 4.01 (H-3⁗) was observed. In the analysis of the HSQC spectrum, it was possible to assign the respective carbons of the IV osidic units (Table 2). By comparing the data in Table 1 and Table 2 with the literature, it was possible to mark osidic unit IV as oleandropyranose [11]. The coupling constant at 9.6 and 1.7 Hz was compatible with β-glycosidic bonding, and the value of C-2⁗ at *δ*_C_ 38.1 allowed the assignment of the configuration of osidic unit IV as β-D-oleandropyranosyl.

**Figure 1:**
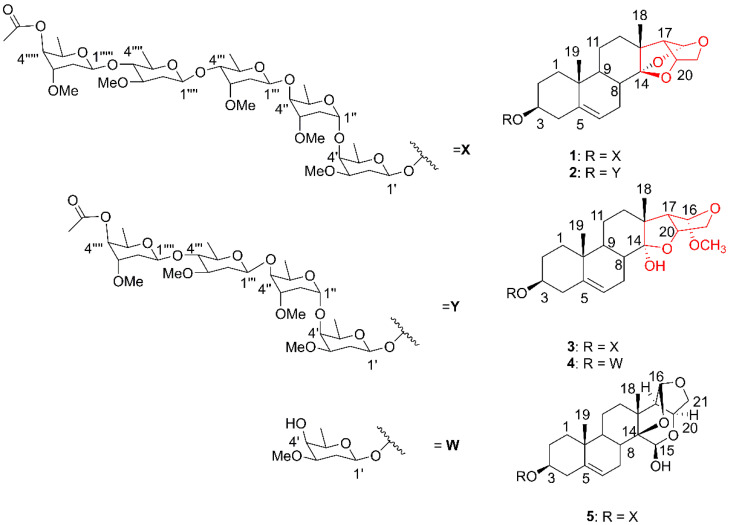
Structures of pregnane glycosides (**1**–**5**) isolated from *M. dardanoi*.

**Figure 2:**
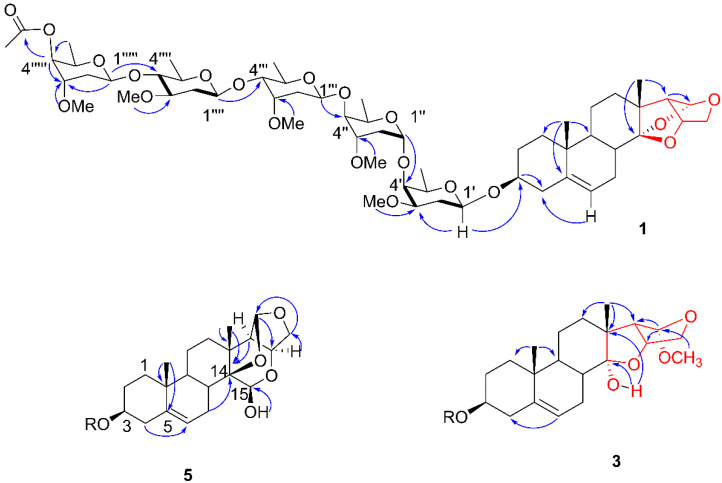
HMBC and COSY correlations of compounds **1**, **3** and **5**: 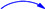 HMBC and 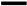 COSY.

Finally, anomeric hydrogen was also observed at *δ*_H_ 4.71 (dd, *J =* 9.7 and 2.0 Hz, H-1′′′′′) correlating in the HSQC spectrum with the carbon at *δ*_C_ 102.9. In HMBC, a correlation of the signal at *δ*_H_ 3.48 (H-4⁗) with the carbon at *δ*_C_ 102 was observed. 9 was observed, confirming the bonding between osidic units IV and V (Figure 1). A correlation of *δ*_H_ 5.15 (H-1′′′′′) with *δ*_H_ 2.25 (H-2′′′′′) was observed in COSY, whose corresponding carbon was signaled by the correlation observed in the HSQC spectrum with the carbon at *δ*_C_ 34.2 (C-2′′′′′). The other data (Table 1 and Table 2) compared with the literature allowed us to assign the glycosidic unit V as simentopyranoside. The coupling constant at 9.7 and 2.0 Hz was compatible with β-glycosidic bonding, and the value of C-2′′′′′ at *δ*_C_ 34.2 allowed the assignment of the configuration of the osidic unit V as β-L-sarmentopyranosyl. The signal at *δ*_H_ 5.38 (d, *J =* 2.9 Hz) was assigned to H-4′′′′′, whose corresponding carbon was marked at *δ*_C_ 68.5 by HSQC. In HMBC, a correlation of the signal at *δ*_H_ 1.29 (d, *J =* 6.4 Hz, 3H-6′′′′′) with *δ*_C_ 68.5 (C-4′′′′′) and of *δ*_H_ 5.38 (H-4′′′′′) with the carbon at *δ*_C_ 171.2 was observed, confirming the position of the acetyl group in osidic unit V at C-4′′′′′ (Figure 2).

After extensive NMR analysis, the structure of **1** was determined to be illustrol-3-O-β-L-diginopyranosyl-(1-4)-α-L-sarmentopyranosyl-(1-4)-β-D-cymaropyranosy-(1-4)-β-D-oleandropyranosyl-(1-4)-β-L-4-acetoxylsarmentopyranosyl, a new natural product named dardanol A.

Compound **2** was isolated as a white powder with a positive optical rotation of [α]^25^_D_ +18 (c 0.1, pyridine). Its molecular formula was determined to be C_50_H_78_O_17_ by HRESIMS, with *m*/*z* 973.5086 [M + Na]^+^ (calcd for C_50_H_78_NaO_17_, 973.5131, Δ = 4.7 ppm), indicating 12 hydrogen deficiency indices. The ^1^H and ^13^C NMR data were similar to compound **1** being assigned to illustrol-type aglycone (Table 1 and Table 2). When compared to compound **1**, a difference of 144 Da was observed in the HRMS spectrum of compound **2**, attributed to the absence of a nonterminal osidic unit. This difference can also be visualized by the presence of the set of signals in the ^1^H NMR spectrum, where four anomeric hydrogen signals are visualized at *δ*_H_ 4.71 (dd, *J =* 9.7, 2.0 Hz), 4.78 (dd, *J* = 9.5, 2.0), 5.15 (dd, *J =* 9.6, 1.6 Hz), and 5.40 (dd, *J =* 4.4, 2.2 Hz), as well as the presence of methyl hydrogen signals at *δ*_H_ 1.51 (3H, d, *J =* 6.4 Hz, C-6′), 1.34 (3H, d, *J =* 6.2 Hz, C-6″), 1.39 (3H, d, *J =* Hz, C-6‴) and 1.28 (3H, d, *J =* 6.2 Hz, C-6⁗), and of the hydrogens of the methoxy groups at *δ*_H_ 3.33 (s, OCH_3_′), *δ*_H_ 3.39 (s, OCH_3_″), *δ*_H_ 3.46 (s, OCH_3_‴) and *δ*_H_ 3.38 (s, OCH_3_⁗), indicated the existence of four osidic units in compound **2**.

The ^1^H and ^13^C NMR data of the osidic units also resembled compound 1, allowing us to establish 2 as illus-trol-3-O-β-L-diginopyranosyl-(1-4)-α-L-sarmentopyranosyl-(1-4)-β-D-cymaropyranosy-(1-4)-β-L-4 acetoxylsarmentopyranosyl, a new natural product named dardanol B.

Compound **3** was isolated as a white powder with a positive optical rotation of [*α*]^25^_D_ +13 (c 0.1, pyridine). Its molecular formula was determined to be C_58_H_94_O_21_ by HRESIMS, with *m*/*z* 1109.6252 [M − H_2_O + H]^+^ (calcd for C_58_H_93_O_20_, 1109.6254, Δ = 1.0 ppm), indicating 12 hydrogen deficiency indices. The NMR data resembled that of the aglycone illustrol; however, some differences were observed for the carbons at *δ*_C_ 38.9, 46 and 62.5, assigned to C-8, C-13 and C-17, respectively, with C-8 and C-17 being deprotected and C-13 protected when compared to the chemical shifts of these carbons in compound **1**. These data were associated with one lower hydrogen deficiency index compared with compound **1**, suggesting the opening of the C-14-O-C-16 epoxide. The signal at *δ*_H_ 6.88 (s), uncorrelated in the HSQC spectrum, was assigned to the OH located at C-14. This signal showed correlations in the HMBC spectrum with the signals at *δ*_C_ 46.0 and 110.1 that were assigned to C-13 and C-14, respectively. Additionally, in the HMBC spectrum, we observed correlations of the signal at *δ*_H_ 1.40 (3H-18) with the carbons at *δ*_C_ 110.1 (C-14) and in the HMBC spectrum at *δ*_C_ 46.0 (C-13) and with *δ*_C_ 62.5, which was assigned to C-17. The signal at *δ*_H_ 5.17, whose corresponding carbon in the HSQC spectrum was *δ*_C_ 105.8, was assigned to C-16. The signal at *δ*_H_ 5.17 correlated with *δ*_C_ 46.0 (C-13) and with *δ*_C_ 62.5 (C-17). A correlation of this signal with *δ*_C_ 71.4, assigned to C-21, was also observed. A correlation was also observed with the signal at *δ*_C_ 54.5 that was assigned to methoxy bound at position 16. The coupling constant of H-17 (d, *J =* 8.0) and a singlet for H-16 demonstrated near 90-degree angulation between these hydrogens, and thus, the aglycone was defined as shown in Figure 1. As far as we have searched, no records were found for this type of aglycone. The NMR data (Table 1 and Table 2), together with literature data, comparisons with compound **1** data and high-resolution mass spectrometry confirmed the presence of five 1–4 bonded osidic units identical to compound **1** and inserted in C-3 (Table 2).

To reject the possibility that the aglycone had been formed in the process of separating the compounds, a direct infusion on the ESIMS/MS was performed with the crude ethanolic extract. The presence of the ion at *m*/*z* 1144.44 [M + NH_4_]^+^ was identified, corresponding to compound **3** (Figure 1). Thus, compound **3** was identified as seco-illustrol-3-O-β-L-diginopyranosyl-(1-4)-α-L-sarmentopyranosyl-(1-4)-β-D-cymaropyranosy-(1-4)-β-D-oleandropyranosyl-(1-4)-β-L-4-acetoxylsarmentopyranosyl, a new natural pro-duct named dardanol C.

Compound **4** was isolated as a white powder with a positive optical rotation of [*α*]^25^_D_ +17 (c 0.1, pyridine). Its molecular formula was determined to be C_28_H_44_O_8_ by HRESIMS, *m*/*z* 531,2923 [M + Na]^+^ (calcd for C_28_H_44_NaO_8_, 531,2928, Δ = 4.2 ppm), indicating 7 hydrogen deficiency indices. The NMR data for the aglycone of this compound were similar to compound **3**, and it could be concluded that it was of the same type as OH at C-14 and OCH_3_ at C-16. In the ^13^C NMR spectrum, it was possible to observe six carbons at *δ*_C_ 99.0, 33.2, 79.4, 67.4, 71.4 and 20.4 in addition to a signal for methoxyl at *δ*_C_ 55.3 compared with the literature, and the other osidic units of compounds **1**–**3** were marked as diginopyranose. The H-1′ coupling constant at 11.6 and 4.0 Hz was compatible with β-glycosidic bonding, and the C-2′ value at *δ*_C_ 33.5 allowed the configuration of the osidic unit to be assigned as L-diginopyranosyl. The molecular mass of this compound was also found in the analysis of the crude extract by ESIMS/MS, which again rejected the possibility of artifacts. Thus, compound **4** was identified as seco-illustrol-3-O-β-L-diginopyranosyl, a new natural product named dardanol D.

Compound **5** was isolated as a white powder with a positive optical rotation of [*α*]^25^_D_ −19 (c 0.1, pyridine). Its molecular formula was determined to be C_58_H_92_O_21_ by HRESIMS, with *m*/*z* 1147.6037 [M + Na]^+^ (calcd for C_57_H_90_NaO_20_, 1147.6023, Δ = 4.8 ppm indicating 13 indices of hydrogen deficiency). The ^1^H and ^13^C NMR data showed signals characteristic of pregnane glycosides. The hydrogen signals at *δ*_H_ 5.00 (d, *J =* 11.5 Hz), 5.89 (d, *J =* 4.4 Hz) and 4.55 (dd, *J =* 6.2, 3.3 Hz) together with carbons at *δ*C 93.5, 109.7 and 74.7 are in agreement with the values described for the velutinol aglycon, reported only in *M. velutinus* [20]. In the RMN ^1^H spectrum, it was possible to observe five signals for anomeric hydrogens at *δ*_H_ 4.74 (dd, *J =* 9.5, 2.0 Hz), 5.50 (m), 5.08 (dd, *J =* 9.6, 1.6 Hz), 5.15 (dd, *J =* 9.6, 1.7 Hz) and 4.71 (dd, *J =* 9.7, 2.0 Hz). These data, compared with compound **1** (Table 1 and Table 2), allowed us to assign the same glycosidic units, with the same sequence of inter-unit bonds and the same configuration. Thus, compound **5** was determined as velutionol-3-*O*-*β*-L-diginopyranosyl-(1-4)-*α*-L-sarmentopyranosyl-(1-4)-*β*-D-cymaropyranosy-(1-4)-*β*-D-oleandropyranosyl-(1-4)-*β*-L-4-acetoxylsarmentopyranosyl, named dardanol E.

### 2.2. Biological Activity

**Figure 3:**
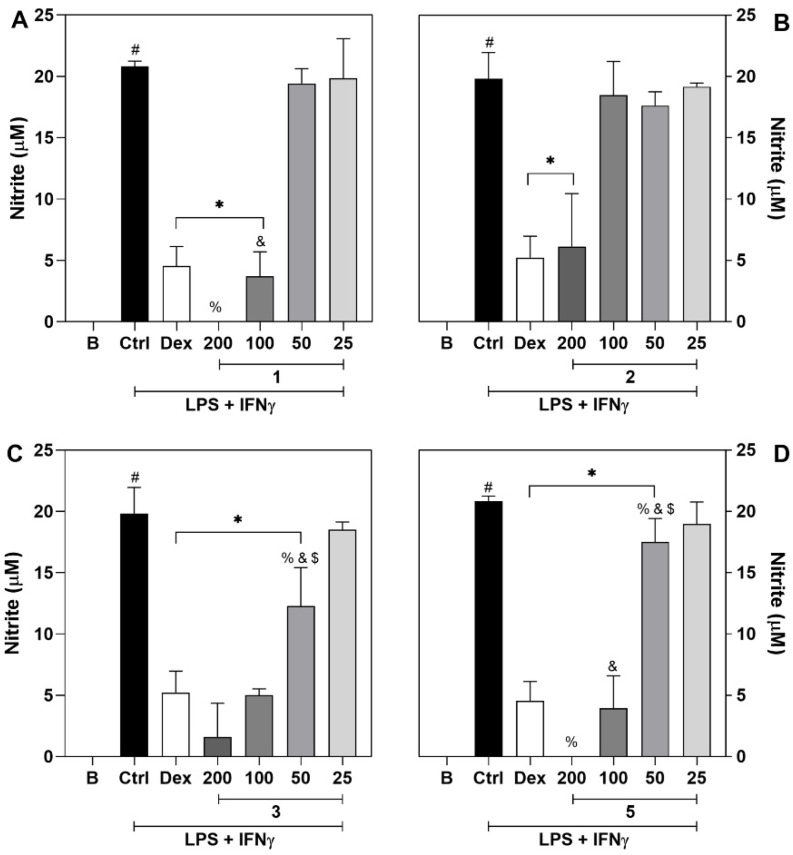
Inhibitory effect of **1**, **2**, **3** and **5** on the production of nitric oxide by stimulated J774 macrophages. Panels (**A**–**D**) show nitrite levels determined by the Griess method, of compounds **1** to **5**, respectively Different concentrations of **1**, **2**, **3** and **5** (25 to200 μM) or dexamethasone (Dex; 20 μM, reference drug) were added to J774 macrophages cultures in the presence of LPS (500 ng/mL) + IFN-γ (5 ng/mL). Nitrite quantifications were performed 24 h after treatments. The control group (Ctrl) represents vehicle treated cells stimulated with LPS + IFN-γ. The basal group (B) shows data from untreated and unstimulated cells. # Different from the unstimulated (B) group (*p* < 0.05). * Different from the control (Ctrl) group (*p* < 0.01). % Different from the Dex group (*p* < 0.05). & Difference from the 200 µM group (*p* < 0.05). $ Difference from the 100 µM group (*p* < 0.05).

To assess the anti-inflammatory potential of compounds, J774 macrophages were used as an in vitro model. Macrophages play a key role in inflammation and immune regulation processes, contributing to tissue homeostasis [21,22]. They are tissue-resident or infiltrated immune cells activated upon stimulation of a great number of pro-inflammatory mediators, such as chemokines, cytokines, and nitric oxide (NO) [21]. Here, the nitric oxide production by macrophages stimulated with Lipopolysaccharides (LPS) and Interferon gamma (IFN-γ) was assessed. Stimulated macrophage that received vehicle as treatment (control group) show an increase in NO levels in comparison to non-stimulated macrophages (basal group, *p* < 0.01; Figure 3A–D). The treatment with all the test compounds inhibited the nitric oxide production at a concentration of 200 μM in comparison to vehicle-treated stimulated cells (Figure 3A–D). Interestingly, the compounds presented different profiles of inhibition. Compound **2** reduced the amount of NO on the supernatant of stimulated cells only at the concentration of 200 μM (Figure 3B; *p* < 0.01), meanwhile compound **1** exhibited a dose-dependent effect at 200 and 100 μM (Figure 3A; *p* < 0.01). Plus, compounds **3** and **5** were able to lower the production of NO at the range of 200 μM to 50 μM (Figure 1D and Figure 3C, respectively; *p* < 0.01), in a dose-dependent manner. Dexamethasone (20 μM), the gold-standard drug, was also able to reduce the levels of NO when compared to untreated cells, as expected (Figure 3A–D; *p* < 0.001). Remarkably, at the concentration of 200 μM compounds **5** and **1** showed greater efficacy in comparison to dexamethasone (*p* < 0.05), while **2** and **3** exhibited a similar efficacy to that of dexamethasone (*p* < 0.05). Importantly, cell toxicity assays (Figure S86; Supplementary Material) show that there was no reduction in cellular viability. These data corroborate the interpretation of the NO assay results, as the decrease of cellular viability could reduce the production of inflammatory mediators, and it could be wrongly acknowledged as anti-inflammatory activity. NO has a ubiquitous role in the maintenance of homeostasis, but upon inflammatory stimuli, it will have mainly a pro-inflammatory role. During the inflammatory response, it will act as an oxidant agent or a signaling mediator, by activating cascades that lead to the production of more inflammatory mediators [23]. Therefore, the ability to reduce the production of this mediator during inflammation is an interesting feature for anti-inflammatory compounds.

**Figure 4:**
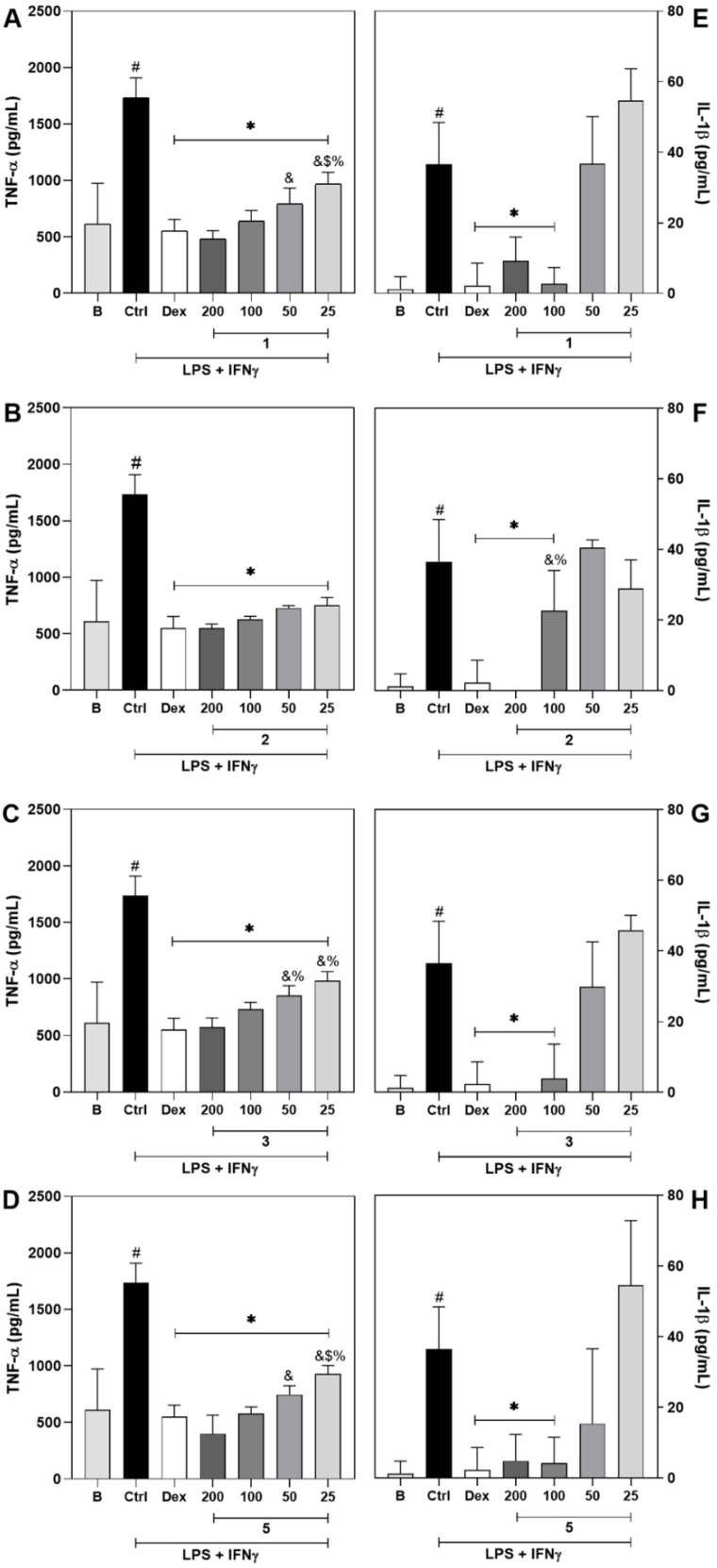
Compounds **1**, **2**, **3** and **5** exhibit a modulatory effect on the production of TNF-α and IL-1β in stimulated J774 macrophages. Panels (**A**–**D**) show the effect of treatment with **1**, **2**, **3** or **5** respectively, on TNF-α levels, measured by ELISA. Panels (**E**–**H**) show the effect of treatment with **1**, **2**, **3** or **5** respectively, on IL-1β levels, measured by ELISA. Different concentrations of **1**, **2**, **3** and **5** (25–200 μM) or dexamethasone (Dex; 20 μM, reference drug) were added to J774 macrophage cultures stimulated with LPS + IFN-γ. The control group (CTRL) represents untreated cells stimulated with LPS + IFN-γ. The basal group (B) shows data from untreated and unstimulated cells. TNF-α and IL-1β levels were determined 4 h and 24 h after treatments, respectively. # Different from the unstimulated (B) group (*p* < 0.05). * Different from the control (Ctrl) group (*p* < 0.01). % Different from the Dex group (*p* < 0.05). & Difference from the 200 µM group (*p* < 0.05). $ Difference from the 100 µM group (*p* < 0.05).

The modulatory effect of **1**, **2**, **3** and **5** on the pro-inflammatory cytokine production by stimulated macrophages was further assessed. The untreated cells stimulated with LPS and IFN-γ (control group) show an increase in Tumor necrosis factor alpha (TNF-α) and Interleukin 1 beta (IL-1β) levels in comparison to non-stimulated macrophage (basal group, *p* < 0.01; Figure 4A–H).

Treatment with all tested concentrations (25–200 µM) of the tested compounds resulted in inhibition of TNF-α production (Figure 4A–D, *p* < 0.01). Within the tested range, most of the molecules showed a dose-dependent effect, except for compound **2** which did not show any difference in the magnitude of its effect among the tested concentrations. Dexamethasone, the gold standard drug, also inhibited the production of TNF-α. Importantly, the effect of compounds **1**, **2**, **3** and **5** (Figure 4A; *p* < 0.01) was comparable to that of dexamethasone.

The production of IL-1β was also modulated by the tested compounds (Figure 4E–H). Compounds **1**, **3** and **5** and **1** on the other hand, display a similar profile. These compounds decrease the amount of IL-1β at concentrations of 200 and 100 µM (Figure 4E–H, *p* < 0.01) in a similar magnitude to that of dexamethasone but did not show any dose-dependency. Similar but not the same, compound **2** inhibited the production of IL-1β at concentrations of 200 and 100 µM (Figure 4F, *p* < 0.01), and showed a dose-dependent profile with an effect similar to dexamethasone.

Together with NO, TNF-α and IL-1β are important mediators of the inflammatory response. TNF-α is a pro-inflammatory cytokine produced primarily by monocytes/macrophages and it plays a key role in the modulation of immune responses and induction of inflammation. Upon activation of its receptor, a cascade of events leading to the production of more pro-inflammatory mediators is initiated [24]. Moreover, TNF-α is a target to treat several inflammatory diseases, such as rheumatoid arthritis, inflammatory bowel diseases, ankylosing spondylitis, and psoriasis [25]. Similarly, IL-1β is also recognized as an important cytokine for inflammatory events. It is primarily a pro-inflammatory cytokine capable of inducing its own production, in a positive feedback loop that amplifies the inflammatory signaling [26]. Plus, the enhanced secretion of IL-1β has been associated with the pathogenesis of autoinflammatory diseases, metabolic syndromes, acute inflammation, chronic inflammation, and malignancy [27]. 

Therefore, the ability of **1**, **2**, **3** and **5** to reduce the production/release of IL-1β and TNF-α reinforces the potential anti-inflammatory activity previously displayed by reducing the amount of NO released by stimulated macrophages. Interestingly, velutinol A, an aglycone steroid compound with a structure similar to the tested compounds, was reported to inhibit kinin B1 receptor-mediated inflammatory responses in vivo [28]. Kinin B1 receptor is an inducible receptor that has been implicated in the process of stimulation and release of IL-1β and TNF-α from macrophages [29].

Data presented here demonstrate that compounds **1**, **2**, **3** and **5** inhibit the production/release of pro-inflammatory mediators, which is an important attribute of anti-inflammatory compounds. Moreover, the tested compounds show a similar or even greater efficacy in comparison to that of dexamethasone, considered the gold-standard drug in the tests. Therefore, these results evidence the anti-inflammatory potential of these compounds. 

## 3. Materials and Methods

### 3.1. General Information

Sephadex LH-20 gel (Merck, Kenilworth, NJ, USA) and commercial octadecylsilane functionalized silica cartridges ISOLUTE^®^ C_18_ (EC) 500 mg/6 mL were used in the pre-purification of the compounds.

The analytical-scale high-performance liquid chromatographic (HPLC) analyses were performed on a Shimadzu Prominence chromatograph, (flow rate of 600 μL-min^−1^) and injections of 20 μL, using a reversed-phase analytical column (YMC, 250 × 4.6 mm and particle size of 5µC_18_). The preparative HPLC analysis was used on Shimadzu Proeminence equipment and reverse phase column (YMC, 250 × 21.2mm and particle size of 5µC_18_). The solvents used were acetonitrile (HPLC grade, Tedia^®^, Cincinnati, OH, USA) and ultrapure water obtained with a Milli-Q (Millipore^®^).

1D and 2D NMR experiments were performed using Bruker Avance III HD (400 and 100 MHz for ^1^H and ^13^C, respectively) and Varian NMR (500 and 125 MHz for ^1^H and ^13^C, respectively) spectrometers. The residual peaks of the deuterated solvents were taken as reference points and chemical shifts were given in ppm. Mass spectrometry analyses were performed on an HRMS microTOFII ESI-TOF.

A HPLC Shimadzu (Kyoto, Japan) coupled with an Amazon X (Bruker Daltonics, Billerica, MA, USA) with an electrospray ion (ESI) source, was used to perform ESI-MSn. The analysis parameters were as follows: capillary 4.5 kV, ESI (positive mode for samples from the chloroform phase and negative mode for samples from the ethyl acetate phase), final plate offset 500 V, 40 psi nebulizer, dry gas (N_2_) with a flow rate of 8 mL/min and a temperature of 200 °C. Collision-induced dissociation (CID) fragmentation was achieved in the Amazon X in auto-MS/MS mode using the enhanced resolution mode. The mass spectra (*m*/*z* 50–1300) were recorded every 2 s. Moreover, these samples were injected again into an HPLC system coupled to a micrOTOF II mass spectrometer (Bruker Daltonics, Billerica, MA, USA) for high resolution electrospray ionization mass spectrometry (HRESIMS) analyses using the same method as previously reported [30].

### 3.2. Plant Material

The roots of *M*. *dardanoi* were collected at Serra do Jatobá (07°29′00″ S, 36°39′54″ O), located in Serra Branca, Paraíba, Brazil. The botanical material was identified by Prof. Dr. José Iranildo Miranda de Melo, from the Department of Biology, Centre of Biological Sciences and Health of the Paraíba State University (UEPB), and later deposited in the Herbarium Manuel de Arruda Câmara (HACAM-UEPB), where a voucher was produced, number 1663. Access registration in the National System for the Management of Genetic Heritage and Associated Traditional Knowledge (SISGEN) was obtained under code A5B0BFC.

### 3.3. Extraction and Isolation

The roots of *M*. *dardanoi* were dried in a circulating air oven at 45 °C for 92 h and then ground in a knife mill, obtaining 1.78 kg of powder. This material was macerated with 95% ethanol for 72 h in five repetitions and the extracted solution was concentrated in a rotary evaporator (40 °C), resulting in 303 g of the crude ethanolic extract (BSE-Md). Subsequently, 290 g of the BSE-Md was solubilized in MeOH/H_2_O (7:3, *v*/*v*). This solution was subjected to partitioning with solvents of an increasing degree of polarity (using 2 L of each solvent: hexane, chloroform, ethyl acetate, and n-butanol). The partitioning allowed obtaining the following phases: hexane (18.2 g), chloroform (12 g), ethyl acetate (4.2 g), and n-butanol (15 g).

For fractionation of the chloroform phase, 3 g were submitted to Sephadex LH-20 gel permeation chromatography, with isocratic elution (MeOH). This method allowed the isolation of 10 fractions (Md-S1 to Md-S10), which were analyzed by CCD. The fractions Md-S1-Md-S5 were subjected to reversed-phase chromatography (C18), with gradient elution using MeOH/H2O (7:3 *v*/*v*), 20 fractions were obtained (Md-C18.1-Md-C18.20). Of these, fractions Md-C18.1 to Md-C18.6 (MeOH/H2O 8:2 *v*/*v*) were pooled, yielding 360 mg, subsequently, this fraction was subjected to preparative HPLC.

In the preparative HPLC analysis mobile phase was composed by binary mixture of water and acetonitrile (70:30, *v*/*v*), through the isocratic elution mode, with a flow rate of 8.0 mL/min, for 80 min. The wavelength used was 205 nm, obtaining in the end 23 fractions. The fractions Md-235-21, Md-235-15, Md-235-16, Md-235-04 and Md-235-18, after undergoing structural elucidation techniques, provided compounds **1** (3.2 mg, *t*_R_ = 53.81 min), **2** (1.8 mg, *t*_R_ = 37.80 min), **3** (2.2 mg, *t*_R_ = 39.48 min), **4** (1.2 mg, *t*_R_ = 12.18 min) and **5** (1.6 mg, *t*_R_ = 46.95 min), respectively.

Nuclear magnetic resonance (NMR) spectra were obtained using spectrometer Bruker 400 MHz (^1^H) and 100 MHz (^13^C) and Varian NMR (500 and 125 MHz for ^1^H and ^13^C, respectively) at the Center of Characterization and Analysis of the Federal University of Paraíba. Deuterated solvent (pyridine-*d*_5_ (C_5_D_5_N) was used in the solubilization of the samples for NMR e chemical shifts (*δ*) were recorded in ppm (parts per million) and coupling constants (*J*) in Hz.

### 3.4. Cytotoxicity to Mammalian Cells

To determine the cytotoxicity of **1**, **2**, **3** and **5**, murine macrophage-like J774 cells were plated into 96-well plates at a cell density of 2 × 10^5^ cells/well in Dulbecco’s modified Eagle medium (DMEM; Life Technologies, GIBCO-BRL, Gaithersburg, MD) supplemented with 10% fetal bovine serum (FBS; GIBCO, Invitrogen, Dun Laoghaire, Ireland) and 50 µg/mL of gentamycin (Novafarma, Anápolis, GO, Brazil), and incubated for 24 h at 37 °C and 5% CO_2_, as previously described [31]. The cells were then stimulated with LPS (500 ng/mL, Sigma Chemical Co., St. Louis, MO, USA) and IFN-γ (5 ng/mL, Sigma). The compounds **1**, **2**, **3** or **5** were added to the medium at five concentrations ranging from 25 to 200 µM in triplicate and incubated for 72 h. After, 20 µL/well of Alamar Blue (Invitrogen, Carlsbad, CA, USA) was added to the plates for 6h. Colorimetric readings were performed at 570 and 600 nm. Gentian violet (Synth, São Paulo, Brazil) at 10 μM was used as the positive control.

### 3.5. Assessment of Cytokine and Nitric Oxide Production by Macrophages

For cytokine and nitric oxide evaluations, J774 cells were seeded in 96-well tissue culture plates at 2 × 10^5^ cells/well in DMEM medium supplemented with 10% of FBS and 50 µg/mL of gentamycin for 2 h at 37 °C and 5% CO_2_, as described previously [32]. Cells were then stimulated with LPS (500 ng/mL) and IFN-γ (5 ng/mL) in the presence of 1, 2, 3 or 5 at different concentrations (25 to 200 µM), medium (control group), or dexamethasone (20 µM, gold-standard drug), and incubated at 37 °C. Cell-free supernatants were collected 4 h after the incubation for TNF-α quantification, or 24 h after the incubation for IL-1β and nitrite quantification. Cytokine concentrations in supernatants from J774 cultures were determined by enzyme-linked immunosorbent assay (ELISA), using the DuoSet kit from R&D Systems (Minneapolis, MN), according to the manufacturer’s instructions. The results were expressed in picograms/mL of IL-1β. Quantification of nitrite as an indicator of nitric oxide production was performed using the Griess method [33].

### 3.6. Statistical Analysis

Data are presented as mean ± standard deviation (SD) of 3 replicates. Comparisons between groups were made using one-way ANOVA with Tukey post-hoc test. Analyses were performed using Prism 8 Computer Software (GraphPad, San Diego, CA, USA), with a statistical significance of *p* < 0.05.

### 3.7. Characterization

**Dardanol A** (**1**): white powder; [α]^25^_D_ + 14 (c 0.1, pyridine); ^1^H and ^13^C NMR data, see Table 1 and Table 2; positive-ion HRESIMS *m*/*z* 1117.5918 [M + Na]^+^ (calcd for C_57_H_90_NaO_20_, 1117.5917).

**Dardanol B** (**2**): white powder; [α]^25^_D_ + 18 (c 0.1, pyridine); ^1^H and ^13^C NMR data, see Table 1 and Table 2; positive-ion HRESIMS *m*/*z* 973.5086 [M + Na]^+^ (calcd for C_50_H_78_NaO_17_, 973.5131).

**Dardanol C** (**3**): white powder; [α]^25^_D_ + 13 (c 0.1, pyridine); ^1^H and ^13^C NMR data, see Table 1 and Table 2; positive-ion HRESIMS *m*/*z* 1109.6252 [M − H_2_O + H]^+^ (calcd for C_58_H_93_O_20_, 1109.6254).

**Dardanol D** (**4**): white powder; [α]^25^_D_ + 17 (c 0.1, pyridine); ^1^H and ^13^C NMR data, see Table 1 and Table 2; positive-ion HRESIMS *m*/*z* 531,2923 [M + Na]^+^ (calcd for C_28_H_44_NaO_8_, 531.2928).

**Dardanol E** (**5**): white powder; [α]^25^_D_ − 19 (c 0.1, pyridine); ^1^H and ^13^C NMR data, see Table 1 and Table 2; positive-ion HRESIMS *m*/*z* 1147.6037 [M + Na]^+^ (calcd for C_57_H_90_NaO_20_, 1147.6023).

## 4. Conclusions

Five new pregnane steroidal glycosides (dardanols A–E) were isolated from the ethanolic extract of *Mandevilla dardanoi* by modern chromatographic techniques and characterized by comprehensive spectroscopic data. Among them, compounds **3** and **4** contained a novel seco-pregnane-type aglycone. Dardanols A, B, C and E showed anti-inflammatory potential by inhibiting the production of nitric oxide and reducing the pro-inflammatory cytokines IL-1β an

d TNF-α in stimulated macrophages. These findings enrich the knowledge of the chemodiversity and biological potential of Caatinga species.

## Figures and Tables

**Table 1:** ^1^H NMR spectroscopic data of the sugar moieties of compounds **1**−**5** (pyridine-*d*_5_).

**Position**	1 ^a^	2 ^a^	3 ^b^	4 ^b^	5 ^b^
1	1.75; 1.04 (m)	1.74; 1.04 (m)	1.76, 1.05 (m)	1.76; 1.05 (m)	1.76 (m); 1.04 (m)
2	2.04; 1.67 (m)	2.04; 1.68 (m)	2.04, 1.68 (m)	2.08; 1.73 (m)	1,90; 1.65 (m)
3	3.81 (m)	3.83 (m)	3.84(m)	3.80 (m)	3.81 (m)
4	2.56 (ddd, 13.4, 4.6, 2.2) 2.38 (m)	2.56 (m) 2.38 (m)	2.59 (m) 2.38 (m)	2.36 (ddd, 13.1, 4.8, 2.2); 2.60 (dd, 13.1, 11.2)	2.46 (m) 2.36 (m)
5	-	-	-	-	-
6	5.41 (m)	5.42 (m)	5.50 (br s)	5.51 (m)	5.39 (d, 2.84)
7	2.50; 2.28 (m)	2.50; 2.28 (m)	2.64 (m)	2.67 (m)	2.56 (m); 2.30 (m)
8	2.02 (m)	2.02 (m)	2.04 (m)	1.81 (m)	2.05 (m)
9	1.55 (m)	1.57 (m)	1.44 (m)	1.46 (m)	1.55
10	-	-	-	-	-
11	1.45 (m)	1.45 (m)	1.53, 1.30 (m)	1.53, 1.38 (m)	1.65 (m)
12	1.54; 1.44 (m)	1.52; 1.46 (m)	1.95; 1.45 (m)	1.99, 1.45 (m)	2.05 (m); 2.28 (m)
13	-	-	-	-	-
14	-	-	-	-	-
15	-	-	-	-	5.00 (d, 11.5)
16	5.75 (d, 2.5)	5.76 (d, 2.5)	5.17 (s)	5.18 (s)	5.89 (d, 4.4)
17	2.52 (t, 2.7)	2.52 (t, 2.7)	3.23 (d, 8.0)	3.25 (d, 8.0)	2.46 (dd, 4.4, 6.0)
18	1.06 (s)	1.06 (s)	1.40 (s)	1.42 (s)	1.02 (s)
19	0.94 (s)	0.94 (s)	0.95 (s)	0.98 (s)	1.00 (s)
20	4.58 (t, 2.4)	4.58 (m)	5.08 (m)	5.10 (ddd, 8.0, 5.2, 1.2)	4.55 (dd, 3.3, 6.2)
21	4.20 (d, 9.9) 3.92 (dd, 9.9, 2.5)	4.21 (d, 9.9) 3.93 (dd, 9.9, 2.5)	4.12 (br d, 9.2) 4.03 (m)	4.23 (dd, 10.1, 1.7) 4.05 (dd, 10.1, 5.3)	4.38 (d, 9.7) 3.81 (dd, 9.84, 3.48)
MeO-16	-	-	3.34 (s)	3.34 (s,)	-
OH-14	-	-	6.88 (s)	6.90 (s)	-
**Osidic units**					
**A**					
1′	4.76 (dd, 9.5, 2.0)	4.78 (dd, 9.5, 2.0)	4.75 (dd, 9.3, 2.2)	4.81 (dd, 11.6, 4.0)	4.74 (dd, 9.5, 2.0)
2′	2.20, 2.13 (m)	2.20, 2.15 (m)	2.16 (m)	2.32 (m); 2.16 (m)	2.20, 2.10
3′	3.41(m)	3.41(m)	3.37 (m)	3.43 (ddd, 12.0, 4.6, 2.9)	3.41
4′	3.94 (m)	3.96 (m)	3.97 (br s)	3.90 (m)	3.94
**5′**	3.56 (qd, 6.4)	3.57 (qd, 6.4)	3.50 (m)	3,56 (dq, 6.5, 1.2)	3.56 (qd, 6.4)
6′	1.49 (d, 6.4)	1.51 (d, 6.4)	1.29 (d, 6.3)	1.55 (d, 6.5)	1.49 (d, 6.4)
MeO	3.33 (s)	3.33 (s)	3.32 (s)	3,40 (s)	3.35 (s)
OH-4′	-	-	-	5.91 (m)	-
**B**					
1″	5.47 (dd, 4.4, 2.2)	5.40 (dd, 4.4, 2.2)	5.47 (dd, 4.7, 2.6)	-	5.50 (m)
2″	2.21, 2.12 (m)	2.20, 2.12 (m)	2.15 (m)	-	2.18, 2.10
3″	3.96 (m)	3.99 (m)	3.97 (m)	-	3.96
4″	3.73 (m)	3.75 (m)	3.73 (m)	-	3.73 (m)
5″	4.56 (dd, 6.6, 1.3)	4.56 (m)	4.56 (dq, 6.1, 1.7)	-	4.56 (dd, 6.6, 1.3)
6″	1.33 (d, 6.2)	1.34 (d, 6.2)	1.32 (d, 6.1)	-	1.33 (d, 6.2)
MeO	3.39 (s)	3.39 (s)	3.39 (s)	-	3.39 (s)
**C**					
1‴	5.15 (dd, 9.6, 1.6)	5.15 (dd, 9.6, 1.7)	5.15 (dd, 9.5, 1.9)	-	5.08 (dd, 9.6, 1.6)
2‴	2.30, 1.90	2.38, 1.86 (m)	2.34, 1.87 (m)	-	2.30, 1.90
3‴	4.07 (m)	4.03 (m)	4.05 (m)	-	4.08 (m)
4‴	3.48 (m)	3.52 (m)	3.45 (m)	-	3.48 (m)
5‴	4.16 (m)	4.19 (m)	4.16 (m)	-	4.16 (m)
6‴	1.33 (d, 6.2)	1.39 (d, 6.2)	1.33 (d, 6.1)	-	1.33 (d, 6.2)
MeO	3.60 (s)	3.46 (s)	3.60 (s)	-	3.61 (s)
**D**				-	
1⁗	5.08 (dd, 9.6, 1.7)	4.71 (dd, 9.7, 2.0)	5.09 (dd, 9.7, 2.2)	-	5.15 (dd, 9.6, 1.7)
2⁗	2.38, 1.80 (m)	2.20, 2.05 (m)	2.31, 1.78 (m)	-	2.35, 1.80
3⁗	4.01 (m)	3.50 (m)	3.99 (m)	-	4.01 (m)
4⁗	3.48 (m)	5.38 (d, 2.9)	3.45 (m)	-	3.48 (m)
5⁗	4.17 (m)	3.67 (dq, 6.4, 0.9)	4.16 (m)	-	4.15 (m)
6⁗	1.39 (d, 6.2)	1.28 (d, 6.4)	1.38 (d, 6.2)	-	1.39 (d, 6.2)
MeO	3.46 (s)	3.38 (s)	3.46 (s)	-	3.49 (s)
**E**					
1′′′′′	4.71 (dd, 9.7, 2.0)	-	4,71 (dd, 9.6, 2.1)	-	4.71 (dd, 9.7, 2.0)
2′′′′′	2.21, 2.05 (m)	-	2,17, 2.04 (m)	-	2.25, 2.10
3′′′′′	3.52 (m)	-	3.48 (m)	-	3.52 (m)
4′′′′′	5.38 (d, 2.9)	-	5.37 (d, 3.0)	-	5.38 (d, 2.9)
5′′′′′	3.69 (dq, 6.4, 0.9)	-	3.68 (dq, 6.5, 1.2)	-	3.70 (m)
6′′′′′	1.29 (d, 6,4)	-	1.50 (d, 6.5)	-	1.30 (d, 6.4)
MeO	3.38 (s)	-	3.32 (s)	-	3.41 (s)
Ac					
4⁗	-	2.01 (s)	-	-	-
4′′′′′	2.02 (s)	-	2.01 (s)	-	2.02 (s)

^a^ Measured at 500 MHz. ^b^ Measured at 400 MHz.

**Table 2:** ^13^C NMR spectroscopic data of compounds **1**−**5** (pyridine-*d*_5_).

Position	1 ^a^	2 ^a^	3 ^b^	4 ^b^	5 ^b^
1	37.1	36.8	37.7	37.7	37.11
2	30.7	30.7	30.5	30.7	30.5
3	77.5	77.6	77.6	77.6	77.4
4	39.8	39.8	39.6	39.7	39.6
5	140.7	140.8	140.2	140.2	140.39
6	122.1	122.2	122.6	122.6	122.28
7	26.0	26.0	26.0	26.0	27.0
8	32.1	32.1	38.9	39.0	34.4
9	46.6	46.7	46.6	46.6	46.5
10	37.4	37.4	37.4	37.4	38.5
11	21.1	21.1	21.9	21.9	19.3
12	30.9	31.0	31.1	31.1	27.2
13	49.1	49.1	46.0	46.0	44.2
14	109.0	109.1	110.1	110.2	87.8
15	-	-	-	-	93.48
16	104.2	104.2	105.8	105.9	109.7
17	56.6	56.7	62.5	62.5	52.9
18	16.9	17.0	20.7	20.7	21.9
19	19.7	19.7	19.7	19.7	19.5
20	78.9	79.0	80.3	80.3	74.7
21	73.9	73.9	71.4	71.4	78.8
MeO-16	-	-	54.4	54.5	-
OH-14	-	-	-	-	-
**Osidic units**					
**A**					
1′	99.0	99.0	98.9	99.0	98.9
2′	28.7	28.8	28.6	33.5	28.70
3′	81.4	81.4	81.3	79.5	81.34
4′	71.0	71.0	70.9	67.4	71.02
**5′**	71.5	71.5	71.4	71.7	71.51
6′	17.5	17.5	17.4	18.0	17.4
MeO	56.5	56.5	56.5	55.6	56.6
**B**					
1″	97.3	97.3	97.2	-	97.3
2″	33.8	33.9	33.7	-	33.83
3″	76.3	76.4	76.2	-	76.30
4″	78.6	78.6	78.5	-	78.6
5″	63.5	63.6	63.4	-	63.52
**6**″	18.4	18.4	18.3	-	18.30
MeO	56.8	56.8	56.7	-	56.7
**C**					
1‴	100.8	-	100.7	-	100.83
2‴	37.0	-	36.9	-	37.03
3‴	78.4	-	78.3	-	78.30
4‴	83.7	-	83.6	-	83.70
5‴	69.4	-	69.5	-	69.5
6‴	18.9	-	18.8	-	18.8
MeO	59.3	-	59.2	-	59.30
**D**				-	
1⁗	100.9	100.8	100.8	-	100.8
2⁗	38.1	38.1	37.0	-	38.1
3⁗	78.1	78.2	78.0	-	78.15
4⁗	83.6	83.8	83.5	-	83.59
5⁗	69.6	69.5	69.3	-	69.58
6⁗	19.0	19.0	18.9	-	18.99
MeO	58.9	58.9	58.8	-	58.92
**E**					
1′′′′′	102.9	103.0	102.8	-	102.90
2′′′′′	34.2	34.2	34.1	-	34.20
3′′′′′	77.4	77.4	77.3	-	77.35
4′′′′′	68.5	68.5	68.4	-	68.48
5′′′′′	71.0	71.2	70.0	-	70.1
6′′′′′	17.2	17.2	17.1	-	17.25
MeO	56.7	56.7	56.5	-	56.65
Ac					
4′′′′′ a	171.2	171.2	171.1	-	171.1
4′′′′′ b	21.0	21.2	21.0	-	21.1

^a^ Measured at 125 MHz. ^b^ Measured at 100 MHz.

## Data Availability

Not applicable.

## Supplementary Materials

The following supporting information can be downloaded at: https://www.mdpi.com/article/10.3390/molecules27185992/s1, Figure S1: ESI-HRMS spectrum of compound **1**; Figure S2: ^1^H NMR spectrum (500 MHz, pyridine-d_5_) of compound **1**; Figures S3–S6: Expansion of ^1^H NMR spectrum (500 MHz, pyridine-d_5_) of compound **1**; Figure S7: APT NMR spectrum (125 MHz, pyridine-d_5_) of compound **1**; Figures S8–S10: Expansion APT NMR spectrum (125 MHz, pyridine-d_5_) of compound **1**; Figure S11: HSQC spectrum(500 and 125 MHz, pyridine-d_5_) of compound **1**; Figures S12–S15: Expansion of HSQC spectrum(500 and 125 MHz, pyridine-d_5_) of compound **1**; Figure S16: HMBC spectrum(500 and 125 MHz, pyridine-d_5_) of compound **1**; Figures S17–S19: Expansion of HMBC spectrum(500 and 125 MHz, pyridine-d_5_) of compound **1**; Figure S20: COSY spectrum (500 MHz, pyridine-d_5_)of compound **1**; Figure S21: NOESY spectrum (500 MHz, pyridine-d_5_) of compound **1**; Figure S22: TOCSY spectrum (500 MHz, pyridine-d_5_) of compound **1**; Figure S23: ESI-HRMS spectrum of compound **2**; Figure S24: ^1^H NMR spectrum (500 MHz, pyridine-d_5_) of compound **2**; Figures S25–S27: Expansion of ^1^H NMR spectrum (500 MHz, pyridine-d_5_) of compound **2**; Figure S28: Dept 135 NMR spectrum (500 MHz, pyridine-d_5_)of compound **2**; Figures S29–S30: Expansion of Dept 135 NMR spectrum (125 MHz, pyridine-d_5_) of compound **2**; Figure S31: HSQC spectrum(500 and 125 MHz, pyridine-d_5,_) of compound **2**; Figures S32–S33: Expansion of HSQC spectrum(500 and 125 MHz, pyridine-d_5_) of compound **2**; Figure S34: HMBC spectrum(500 and 125 MHz, pyridine-d_5_) of compound **2**; Figure S35–S36: Expansion of HMBC spectrum(500 and 125 MHz, pyridine-d5) of compound **2**; Figure S37: COSY spectrum (500 MHz, pyridine-d_5_) of compound **2**; Figure S38: ESI-HRMS spectrum of compound **3**; Figure S39: ^1^H NMR spectrum (400 MHz, pyridine-d_5_) of compound **3;** Figures S40–S42: Expansion of ^1^H NMR spectrum (400 MHz, pyridine-d_5_) of compound **3**; Figure S43: APT NMR spectrum (400 MHz, pyridine-d_5_) of compound **3**; Figure S44–S45: Expansion of APT NMR spectrum (400 MHz, pyridine-d5) of compound **3**; Figure S46: HSQC spectrum(400 MHz, pyridine-d5) of compound **3**; Figures S47–S50: Expansion of HSQC spectrum(400 and 100 MHz, pyridine-d_5_) of compound **3**; Figure S51: HMBC spectrum(400 and 100 MHz, pyridine-d_5_) of compound **3**; Figure S52: Expansion of HMBC spectrum(400 and 100 MHz, pyridine-d_5_) of compound **3**; Figure S53: HMBC spectrum(400 and 100 MHz, pyridine-d_5_) of compound **3**; Figure S54: COSY NMR spectrum (400 MHz, pyridine-d_5_) of compound **3**; Figure S55: ESI-HRMS spectrum of compound **4**; Figure S56: ^1^H NMR spectrum (400 MHz, pyridine-d_5_) of compound **4**; Figure S57: Expansion of ^1^H NMR spectrum (400 MHz, pyridine-d_5_) of compound **4**; Figures S58–S59: ^1^H NMR spectrum (400 MHz, pyridine-d_5_) of compound **4**; Figure S60: ^13^C NMR spectrum (100 MHz, pyridine-d_5_) of compound **4**; Figure S61: DEPT 135 spectrum (100 MHz, pyridine-d_5_) of compound **4**; Figure S62: Expansion of DEPT 135 spectrum (100 MHz, pyridine-d_5_) of compound **4**; Figure S63: HSQC spectrum (400 and 100 MHz, pyridine-d_5_) of compound **4**; Figures S64–S65: Expansion of HSQC spectrum (400 and 100 MHz, pyridine-d_5_) of compound **4**; Figure S66: HMBC spectrum (400 and 100 MHz, pyridine-d_5_) of compound **4**; Figure S67: Expansion of HMBC spectrum (400 and 100 MHz, pyridine-d_5_) of compound **4**; Figure S68: HMBC spectrum (400 and 100 MHz, pyridine-d_5_) of compound **4**; Figure S69: COSY spectrum (400 MHz, pyridine-d5) of compound **4**; Figure S70: ESI-HRMS spectrum of compound **5**; Figure S71: ^1^H NMR spectrum (400 MHz, pyridine-d_5_) of compound **5**; Figures S72–S73: Expansion of ^1^H NMR spectrum (400 MHz, pyridine-d_5_) of compound **5**; Figure S74: ^13^C NMR spectrum (100 MHz, pyridine-d_5_) of compound **5**; Figures S75–S76: Expansion of ^13^C NMR spectrum (100 MHz, pyridine-d_5_)of compound **5**; Figure S77: DEPT 135 spectrum of compound **5**; Figure S78: DEPT 135 spectrum (100 MHz, pyridine-d_5_)of compound **5**; Figures S79–S81: HSQC spectrum (400 and 100 MHz, pyridine-d_5_) of compound **5**; Figure S82: HMBC spectrum (400 and 100 MHz, pyridine-d_5_) of compound **5**; Figures S83–S84: Expansion of HMBC spectrum (400 and 100 MHz, pyridine-d_5_) of compound **5**; Figure S85: COSY spectrum (400 MHz, pyridine-d_5_) of compound **5**; Figure S86: Effect of 1, 2, 3 and 5 on cell viability of stimulated J774 macrophages.

## Sample Availability

Samples of the compounds are not available from the authors.
