# Supplementary material for: New Pregnane Glycosides from Mandevilla dardanoi and Their Anti-Inflammatory Activity
Source: Molecules. 2022 Sep 14;27(18):5992. doi: 10.3390/molecules27185992 (PMC9501082; doi:10.3390/molecules27185992)

Supplementary Material for:

New Pregnane Glycosides from *Mandevilla dardanoi* and Their Anti-inflammatory Activity

Francisca S. V. Lins [1], Thalisson A. de Souza [1], Luiza C. F. Opretzka [2], Joanda P. R. e Silva [1], Laiane C. O. Pereira [1], Lucas S. Abreu [2], Anderson A. V. Pinheiro [1], George Luís Dias dos Santos [1], Yuri M. do Nascimento [1], José I. M. de Melo [3], Raimundo Braz-Filho [4], Cristiane Flora Villarreal [2], Marcelo S. da Silva [1] and Josean F. Tavares [1,*]

[1] Postgraduate Program in Natural and Synthetic Bioactive Products, Federal University of Paraíba, João Pessoa, Brazil

[2] School of Pharmacy, Federal University of Bahia, Salvador, Brazil

[3] Department of Chemistry, Institute of Chemistry, Fluminense Federal University, Niterói, Brazil

[4] Department of Biological Sciences, State University of Paraíba, 58429-500 Campina Grande-PB, Brazil

[5] Department of Chemistry, Institute of Chemistry, Federal Rural University of Rio de Janeiro, Seropédica, Brazil

*Corresponding author: Josean Fechine Tavares; Instituto de Pesquisa em Fármacos e Medicamentos - Programa de Pós-Graduação em Produtos Naturais e Sintéticos Bioativos, Universidade Federal da Paraíba, 58051-900 João Pessoa-PB, Brazil. Tel.: + 55 83 988027809; E-mail address: josean@ltf.ufpb.br; ORCID ID: 6009412640611523.

Figure S1 ESI-HRMS spectrum of compound **1**
Figure S2 ^{1}H NMR spectrum (500 MHz, pyridine-d_5) of compound **1**.
Figure S3 Expansion of ^{1}H NMR spectrum (500 MHz, pyridine-d_5) of compound **1**.
Figure S4 Expansion of ^{1}H NMR spectrum (500 MHz, pyridine-d_5) of compound **1**.
Figure S5 Expansion of ^{1}H NMR spectrum (500 MHz, pyridine-d_5) of compound **1**.
Figure S6 Expansion of ^{1}H NMR spectrum (500 MHz, pyridine-d_5) of compound **1**.
Figure S7 APT NMR spectrum (125 MHz, pyridine-d_5) of compound **1**
Figure S8 Expansion APT NMR spectrum (125 MHz, pyridine-d_5) of compound **1**
Figure S9 Expansion of APT NMR spectrum (125 MHz, pyridine-d_5) of compound **1**
Figure S10 Expansion of APT NMR spectrum (125 MHz, pyridine-d_5) of compound **1**
Figure S11 HSQC spectrum(500 and 125 MHz, pyridine-d_5) of compound **1**.
Figure S12 Expansion of HSQC spectrum(500 and 125 MHz, pyridine-d_5) of compound 1.
Figure S13 Expansion of HSQC spectrum(500 and 125 MHz, pyridine-d_5) of compound 1.
Figure S14 Expansion of HSQC spectrum(500 and 125 MHz, pyridine-d_5) of compound 1.
Figure S15 Expansion of HSQC spectrum(500 and 125 MHz, pyridine-d_5) of compound 1.
Figure S16 HMBC spectrum(500 and 125 MHz, pyridine-d_5) of compound **1**.
Figure S17 Expansion of HMBC spectrum(500 and 125 MHz, pyridine-d_5) of compound **1**.
Figure S18 Expansion of HMBC spectrum(500 and 125 MHz, pyridine-d_5) of compound **1**.
Figure S19 Expansion of HMBC spectrum(500 and 125 MHz, pyridine-d_5) of compound **1**.
Figure S20 COSY spectrum (500 MHz, pyridine-d_5)of compound **1**.
Figure S21 NOESY spectrum (500 MHz, pyridine-d_5) of compound **1**.
Figure S22 TOCSY spectrum (500 MHz, pyridine-d_5) of compound **1**.
Figure S23 ESI-HRMS spectrum of compound **2**
Figure S24 ^{1}H NMR spectrum (500 MHz, pyridine-d_5) of compound **2**
Figure S25 Expansion of ^{1}H NMR spectrum (500 MHz, pyridine-d_5) of compound **2.**
Figure S26 Expansion of ^{1}H NMR spectrum (500 MHz, pyridine-d_5)of compound **2.**
Figure S27 Expansion of ^{1}H NMR spectrum (500 MHz, pyridine-d_5)of compound **2.**
Figure S28 Dept 135 NMR spectrum (500 MHz, pyridine-d_5)of compound **2.**
Figure S29 Expansion of Dept 135 NMR spectrum (125 MHz, pyridine-d_5) of compound **2.**
Figure S30 Expansion of Dept 135 NMR spectrum (125 MHz, pyridine-d_5) of compound **2.**
Figure S31 HSQC spectrum(500 and 125 MHz, pyridine-$d_{5,}$) of compound 2
Figure S32 Expansion of HSQC spectrum(500 and 125 MHz, pyridine-d_5) of compound 2
Figure S33 Expansion of HSQC spectrum(500 and 125 MHz, pyridine-d5) of compound 2
Figure S34 HMBC spectrum(500 and 125 MHz, pyridine-d_5) of compound 2
Figure S35 Expansion of HMBC spectrum(500 and 125 MHz, pyridine-d5) of compound 2
Figure S36 Expansion of HMBC spectrum(500 and 125 MHz, pyridine-d5) of compound 2
Figure S37 COSY spectrum (500 MHz, pyridine-d_5) of compound 2.
Figure S38 ESI-HRMS spectrum of compound **3**
Figure S39 ^{1}H NMR spectrum (400 MHz, pyridine-d_5) of compound **3**
Figure S40 Expansion of ^{1}H NMR spectrum (400 MHz, pyridine-d_5) of compound **3**
Figure S41 Expansion of ^{1}H NMR spectrum (400 MHz, pyridine-d_5) of compound **3**
Figure S42 Expansion of ^{1}H NMR spectrum (400 MHz, pyridine-d_5) of compound **3**
Figure S43 APT NMR spectrum (400 MHz, pyridine-d_5) of compound **3**
Figure S44 Expansion of APT NMR spectrum (400 MHz, pyridine-d5) of compound **3**

Figure S45 Expansion of APT NMR spectrum (400 MHz, pyridine-d5) of compound **3**
Figure S46 HSQC spectrum(400 MHz, pyridine-d5) of compound **3**
Figure S47 Expansion of HSQC spectrum(400 and 100 MHz, pyridine-d_5) of compound **3**
Figure S48 Expansion of HSQC spectrum(400 and 100 MHz, pyridine-d_5) of compound **3**
Figure S49 Expansion of HSQC spectrum(400 and 100 MHz, pyridine-d_5) of compound **3**
Figure S50 Expansion of HSQC spectrum(400 and 100 MHz, pyridine-d_5) of compound **3**
Figure S51 HMBC spectrum(400 and 100 MHz, pyridine-d_5) of compound **3**
Figure S52 Expansion of HMBC spectrum(400 and 100 MHz, pyridine-d_5) of compound **3**
Figure S53 HMBC spectrum(400 and 100 MHz, pyridine-d_5) of compound **3**
Figure S54 COSY NMR spectrum (400 MHz, pyridine-d_5) of compound **3**
Figure S55 ESI-HRMS spectrum of compound **4**
Figure S56 ^{1}H NMR spectrum (400 MHz, pyridine-d_5) of compound **4**
Figure S57 Expansion of ^{1}H NMR spectrum (400 MHz, pyridine-d_5) of compound **4**
Figure S58 ^{1}H NMR spectrum (400 MHz, pyridine-d_5) of compound **4**
Figure S59 ^{1}H NMR spectrum (400 MHz, pyridine-d_5) of compound **4**
Figure S60 ^{13}C NMR spectrum (100 MHz, pyridine-d_5) of compound **4**
Figure S61 DEPT 135 spectrum (100 MHz, pyridine-d_5) of compound **4**
Figure S62 Expansion of DEPT 135 spectrum (100 MHz, pyridine-d_5) of compound **4**
Figure S63 HSQC spectrum (400 and 100 MHz, pyridine-d_5) of compound **4**
Figure S64 Expansion of HSQC spectrum (400 and 100 MHz, pyridine-d_5) of compound **4**
Figure S65 Expansion of HSQC spectrum (400 and 100 MHz, pyridine-d_5) of compound **4**
Figure S66 HMBC spectrum (400 and 100 MHz, pyridine-d_5) of compound **4**
Figure S67 Expansion of HMBC spectrum (400 and 100 MHz, pyridine-d_5) of compound **4**
Figure S68 HMBC spectrum (400 and 100 MHz, pyridine-d_5) of compound **4**
Figure S69 COSY spectrum (400 MHz, pyridine-d5) of compound **4**
Figure S70 ESI-HRMS spectrum of compound **5**
Figure S71 ^{1}H NMR spectrum (400 MHz, pyridine-d_5) of compound **5**
Figure S72 Expansion of ^{1}H NMR spectrum (400 MHz, pyridine-d_5) of compound **5**
Figure S73 Expansion of ^{1}H NMR spectrum (400 MHz, pyridine-d_5) of compound **5**
Figure S74 ^{13}C NMR spectrum (100 MHz, pyridine-d_5) of compound **5**
Figure S75 Expansion of ^{13}C NMR spectrum (100 MHz, pyridine-d_5)of compound **5**
Figure S76 Expansion of ^{13}C NMR spectrum (100 MHz, pyridine-d_5)of compound **5**
Figure S77 DEPT 135 *spectrum of compound* **5**
Figure S78 DEPT 135 *spectrum (100 MHz, pyridine-d_5)of compound* **5**
Figure S79 HSQC spectrum (400 and 100 MHz, pyridine-d_5) of compound **5**
Figure S80 HSQC spectrum (400 and 100 MHz, pyridine-d_5) of compound **5**
Figure S81 HSQC spectrum o(400 and 100 MHz, pyridine-d_5) of compound **5**
Figure S82 HMBC spectrum (400 and 100 MHz, pyridine-d_5) of compound **5**
Figure S83 Expansion of HMBC spectrum (400 and 100 MHz, pyridine-d_5) of compound **5**
Figure S84 Expansion of HMBC spectrum (400 and 100 MHz, pyridine-d_5) of compound **5**
Figure S85 COSY spectrum (400 MHz, pyridine-d_5) of compound **5**
Figure S86 Effect of 1, 2, 3 and 5 on cell viability of stimulated J774 macrophages

Figure S1: ESI-HRMS spectrum of compound ***1***

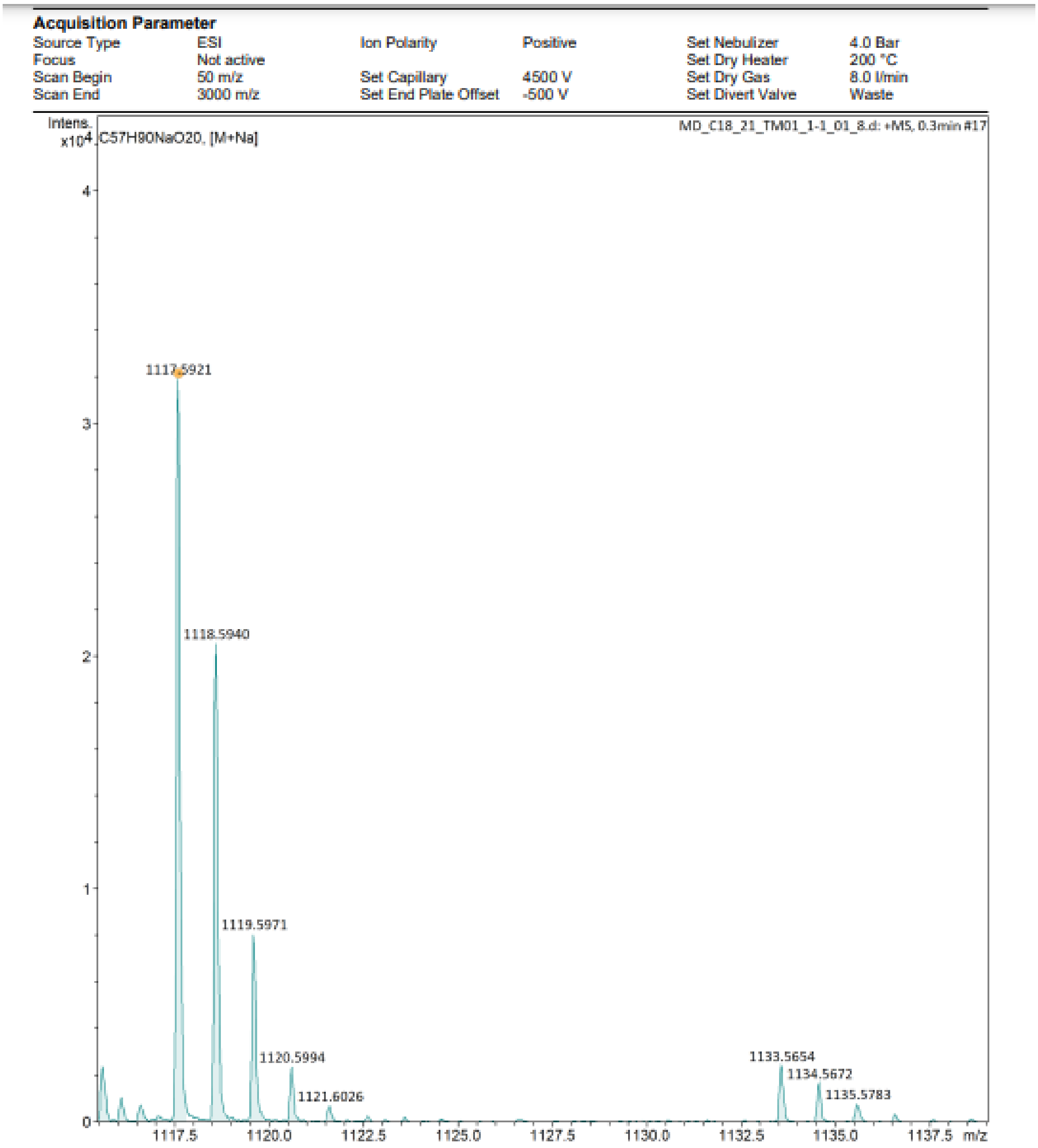

Figure S2: ^{1}H NMR spectrum (pyridine-d$_5$, 500 MHz) of compound ***1***

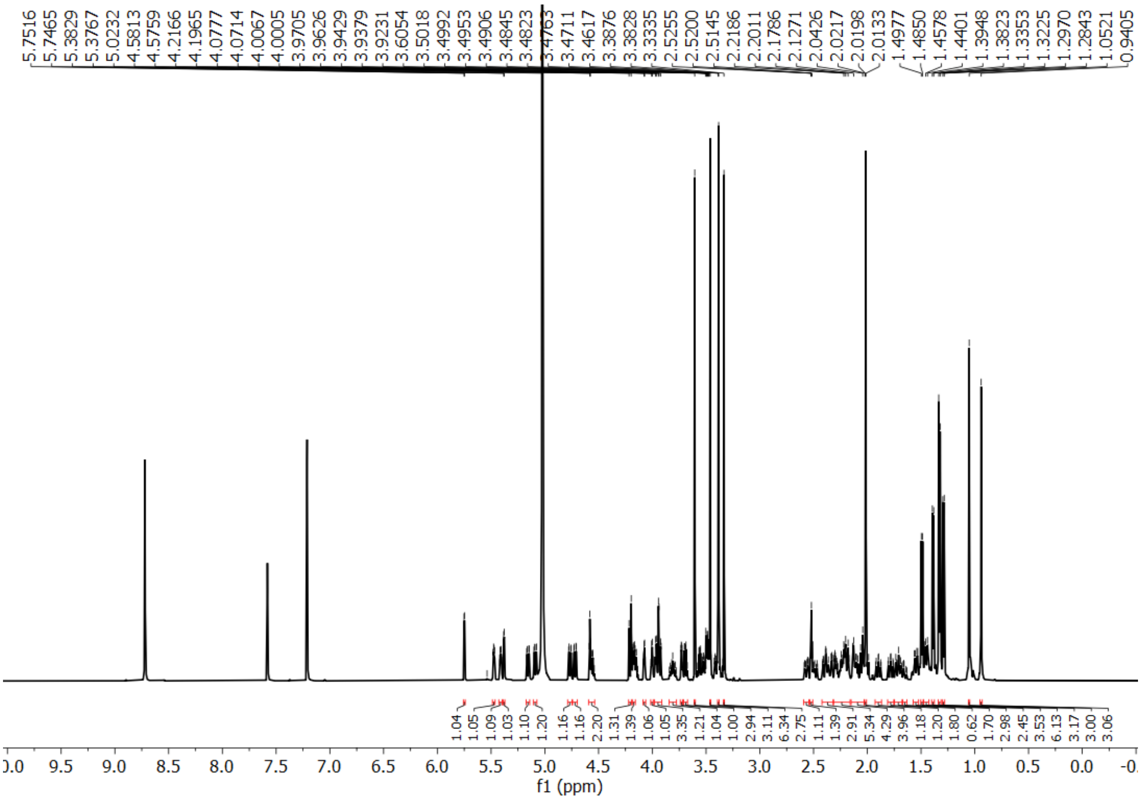

Figure S3: Expansion of ^{1}H NMR spectrum (500 MHz, pyridine-d$_5$) of compound ***1***.

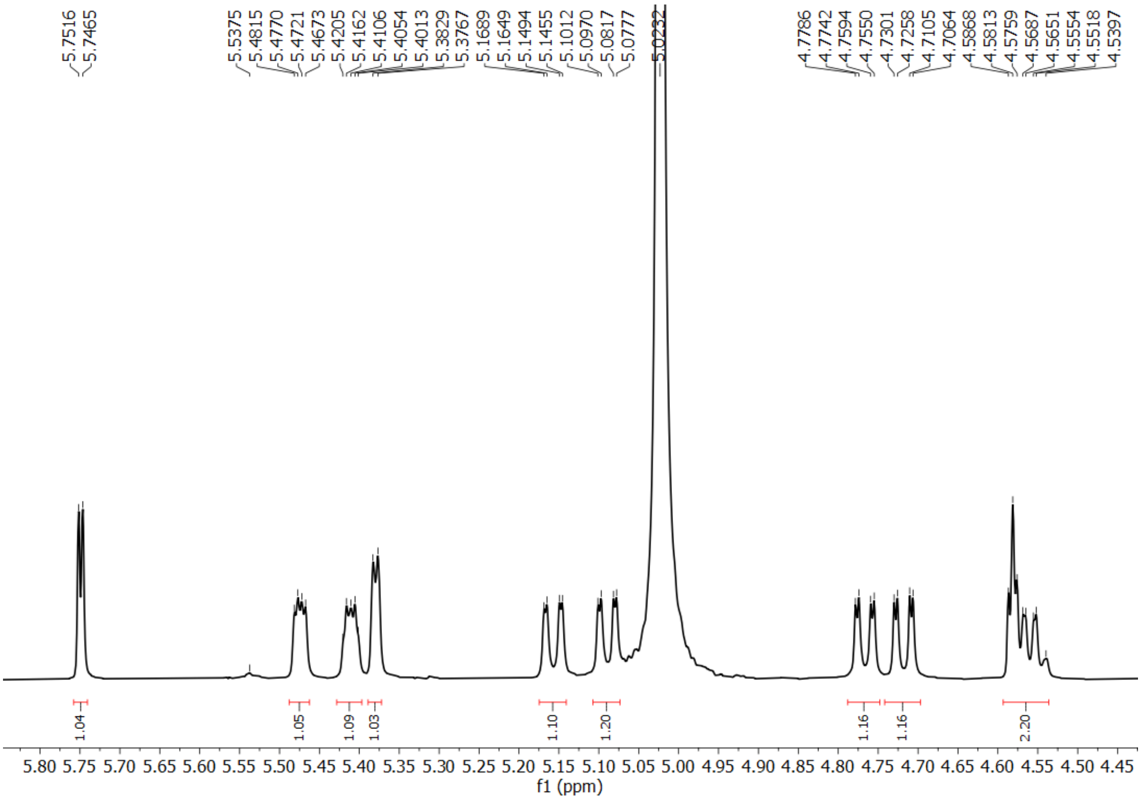

Figure S4: Expansion of ^{1}H NMR spectrum (500 MHz, pyridine-d_5) of compound ***1***.

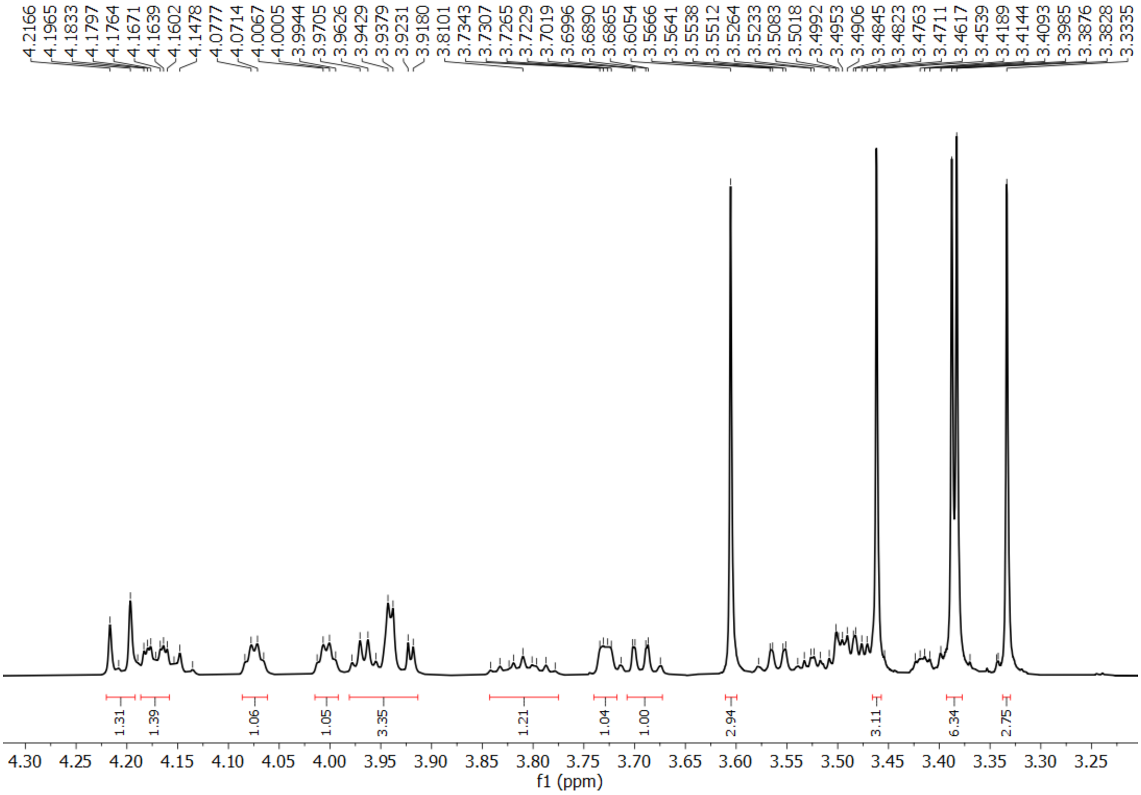

Figure S5: Expansion of ^{1}H NMR spectrum (500 MHz, pyridine-d_5) of compound ***1***.

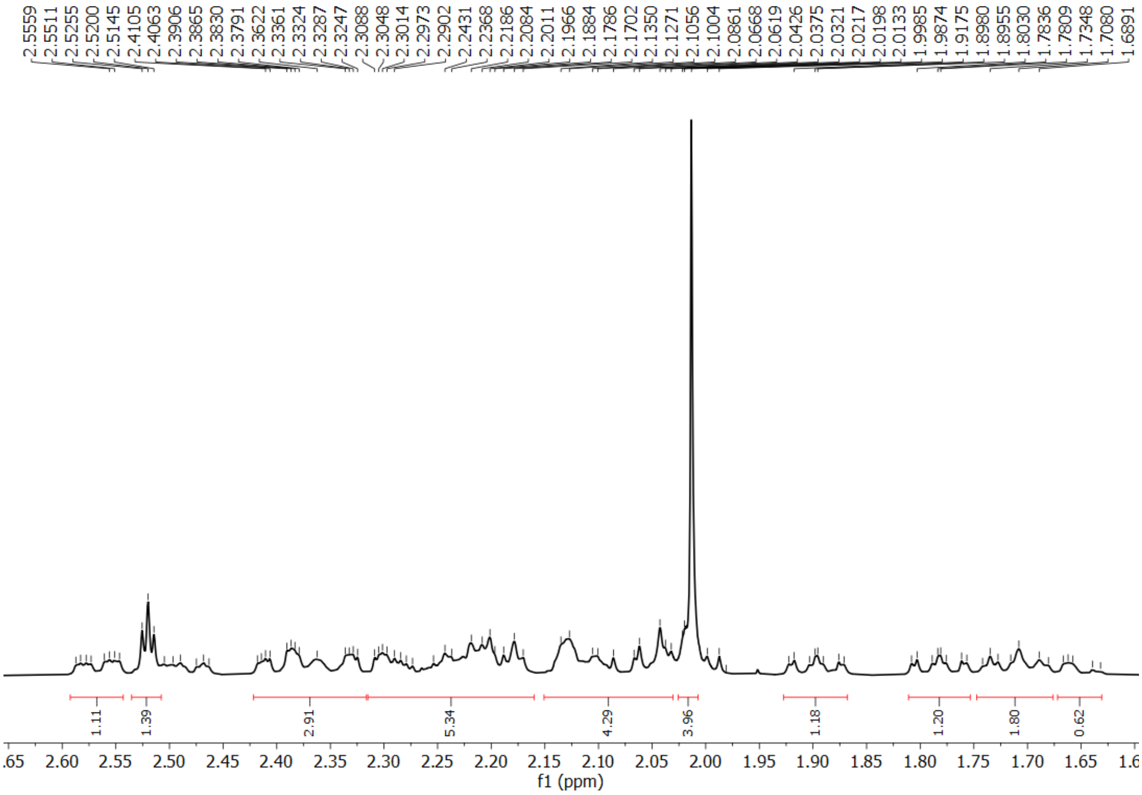

Figure S6: Expansion of ^{1}H NMR spectrum (500 MHz, pyridine-d_5) of compound ***1***.

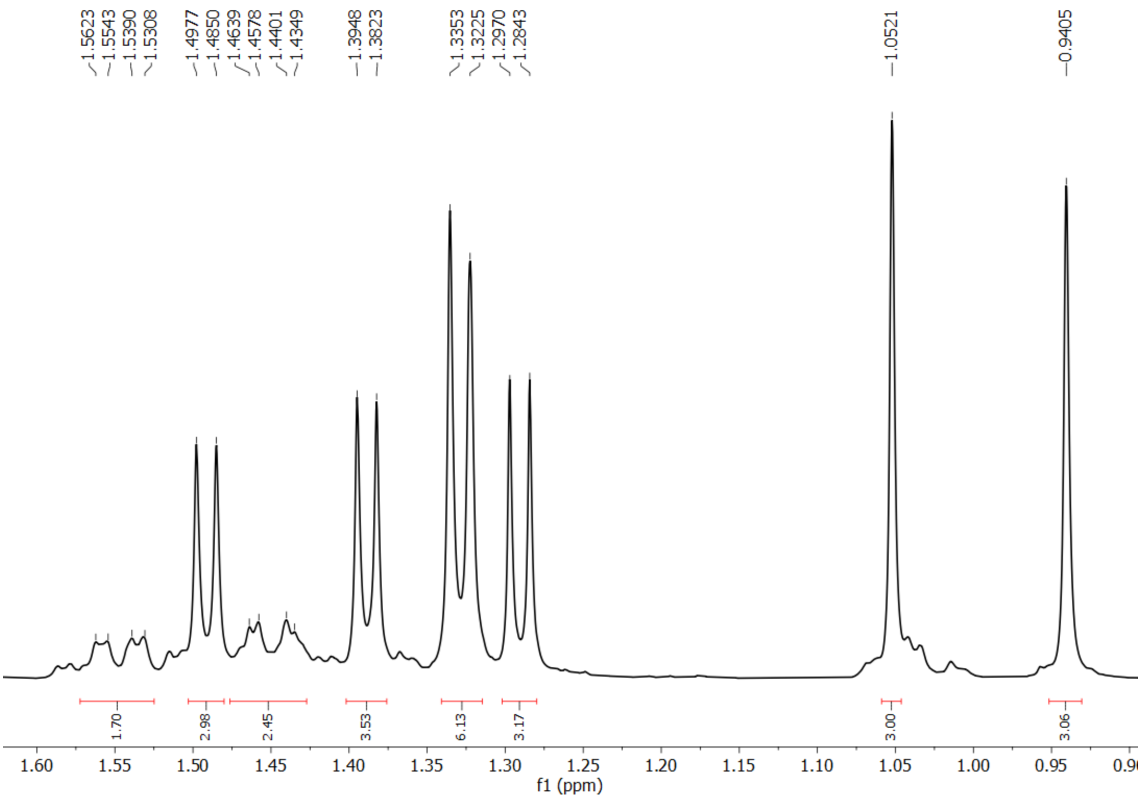

Figure S7: APT NMR spectrum (pyridine-d_5, 125 MHz) of compound ***1***.

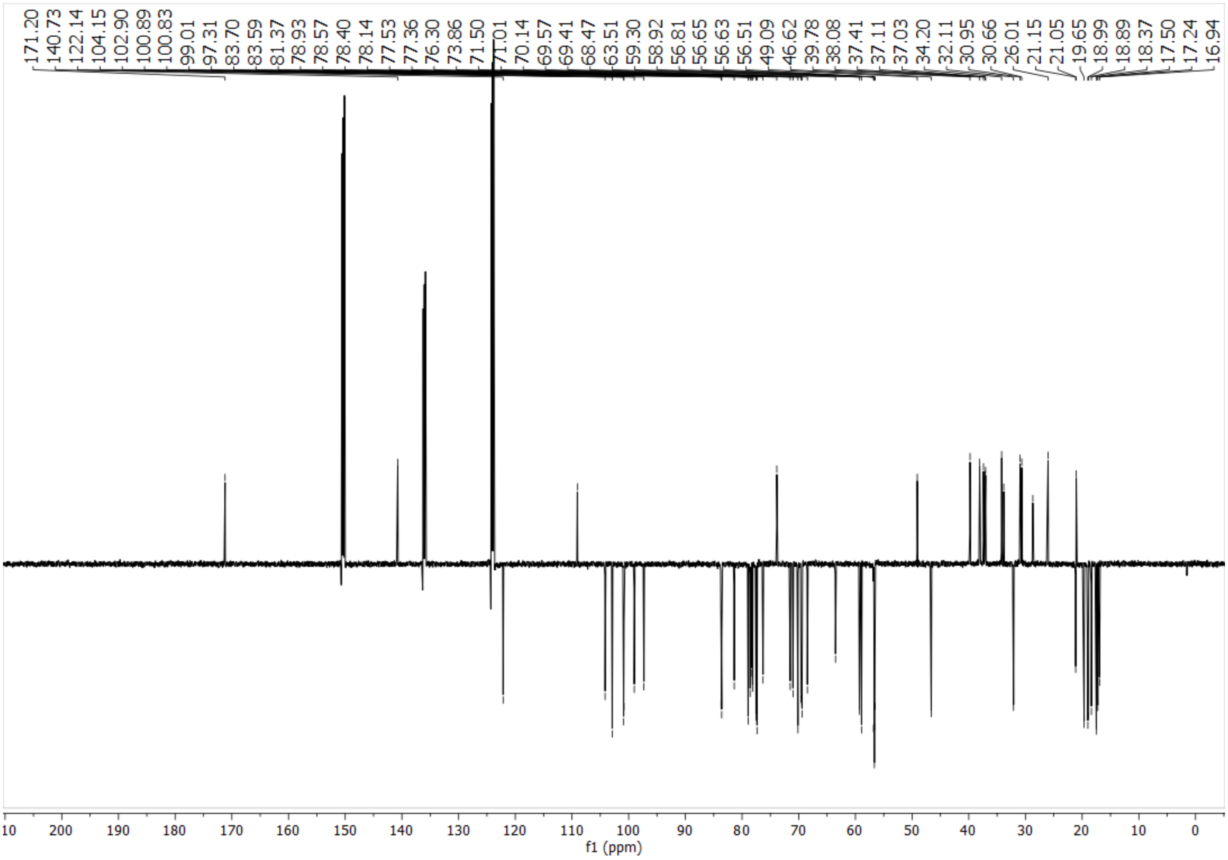

Figure S8: Expansion of APT NMR spectrum (pyridine-d5, 125 MHz) of compound ***1***.

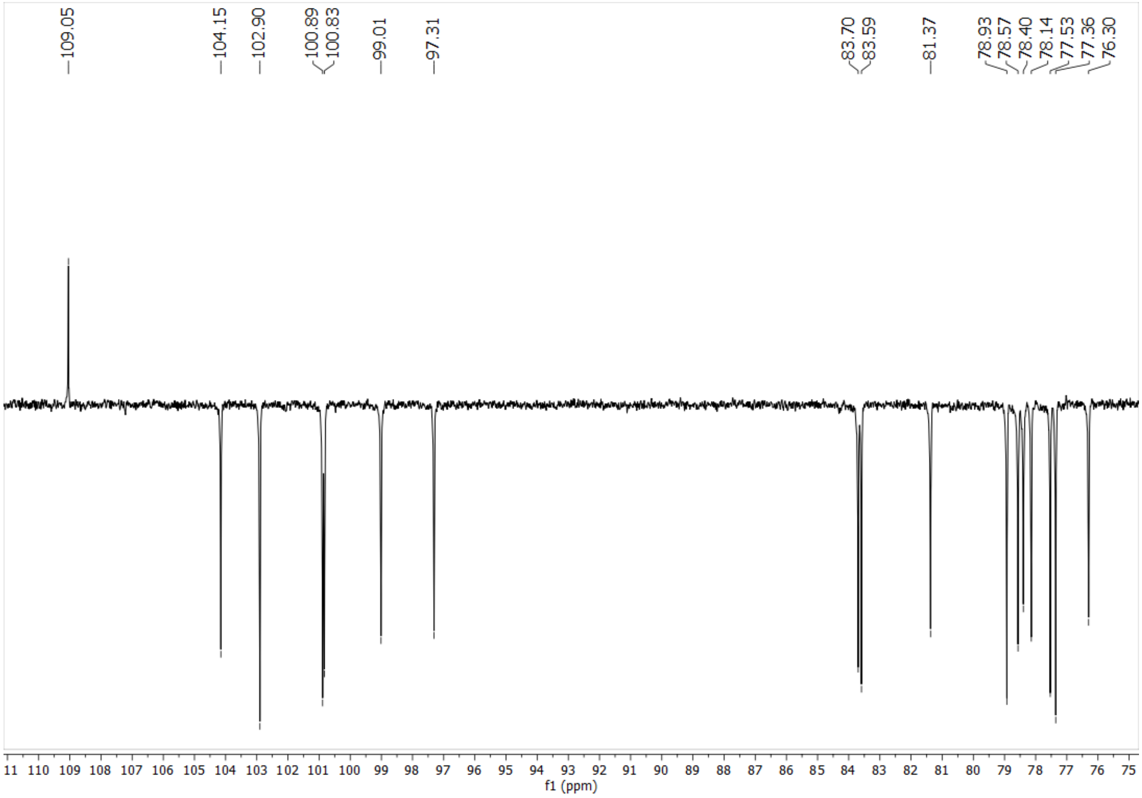

Figure S9: Expansion of APT NMR spectrum (pyridine-d5, 125 MHz) of compound ***1***.

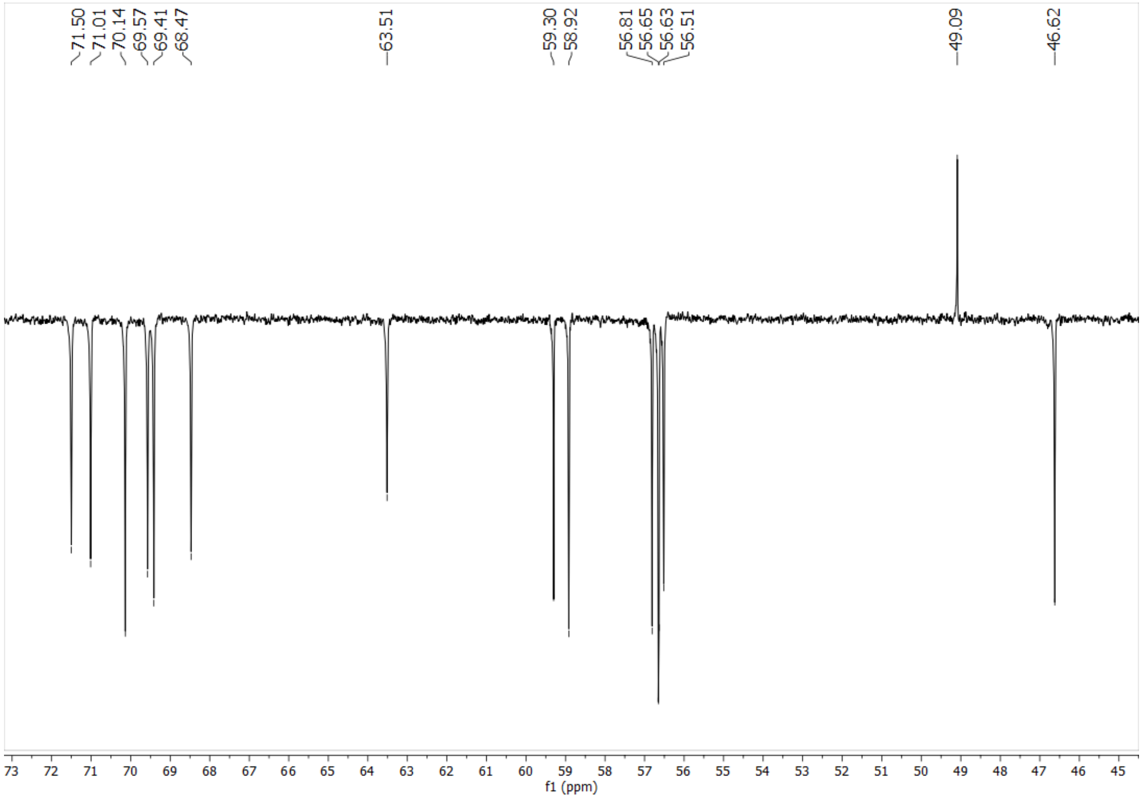

Figure S10: Expansion of APT NMR spectrum (pyridine-d5, 125 MHz) of compound ***1***.

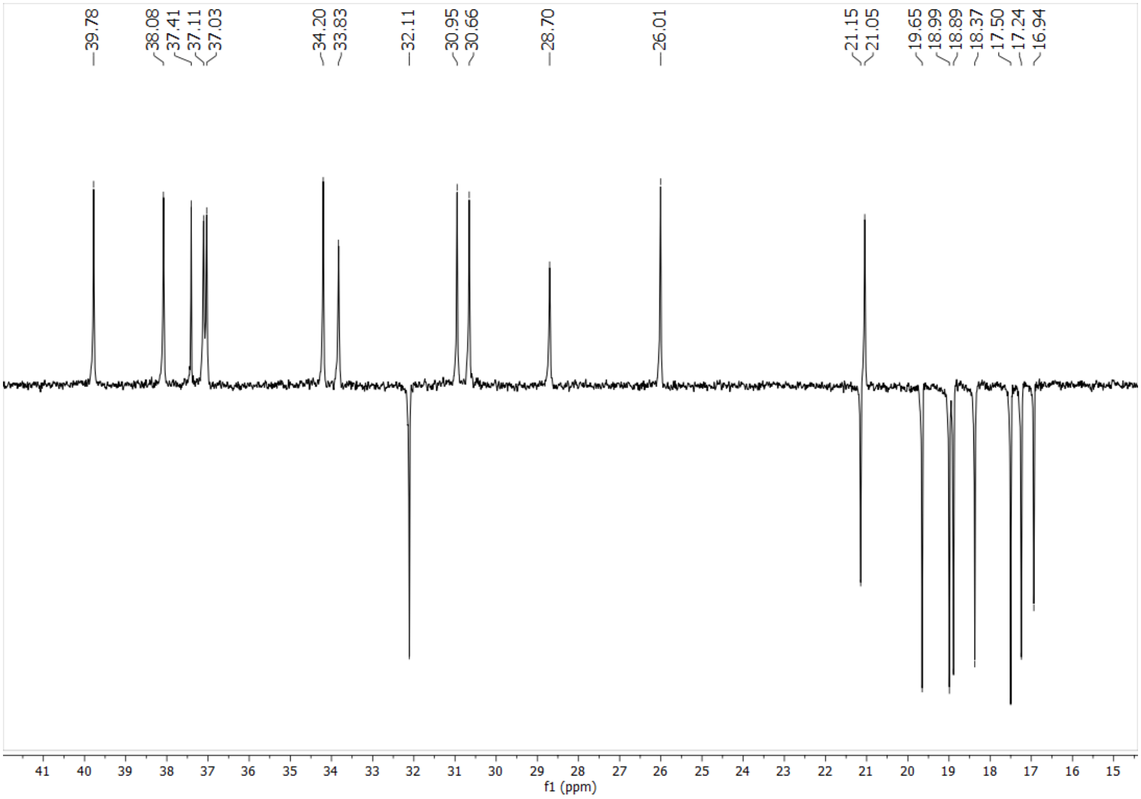

Figure S11: HSQC spectrum(500 and 125 MHz, pyridine-d5) of compound ***1***.

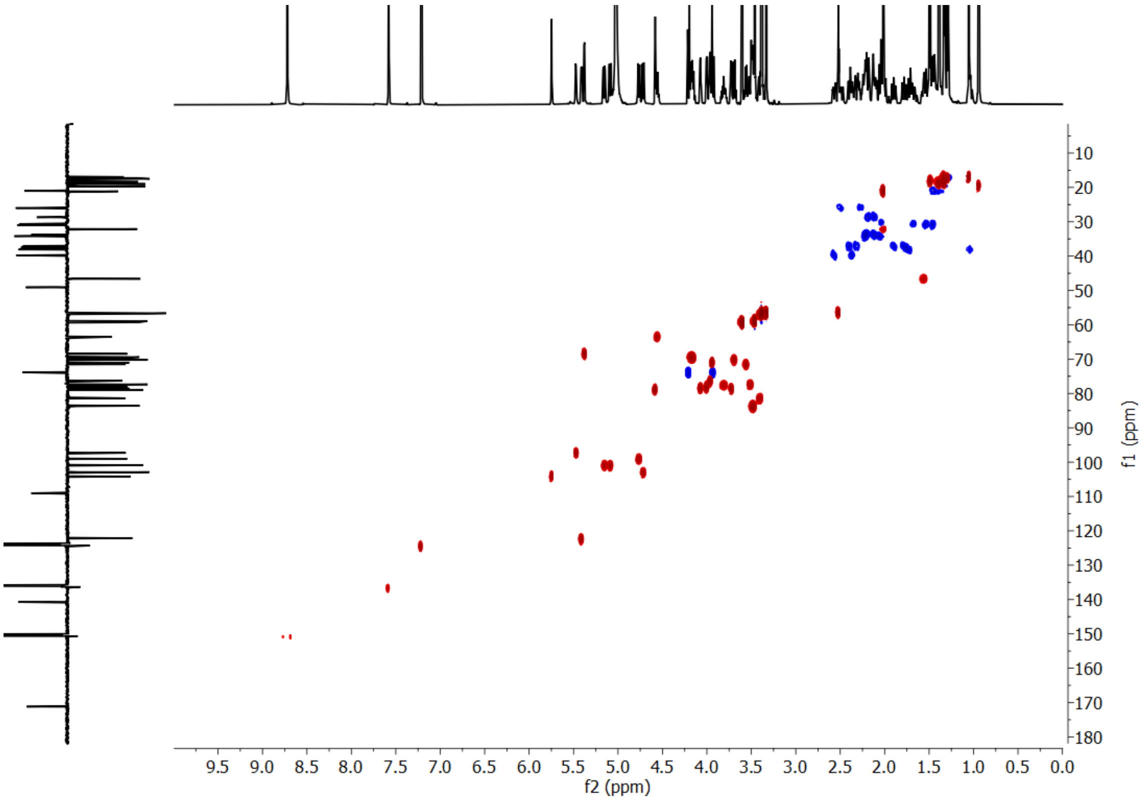

Figure S12: Expansion of HSQC spectrum(500 and 125 MHz, pyridine-d_5) of compound *1*.

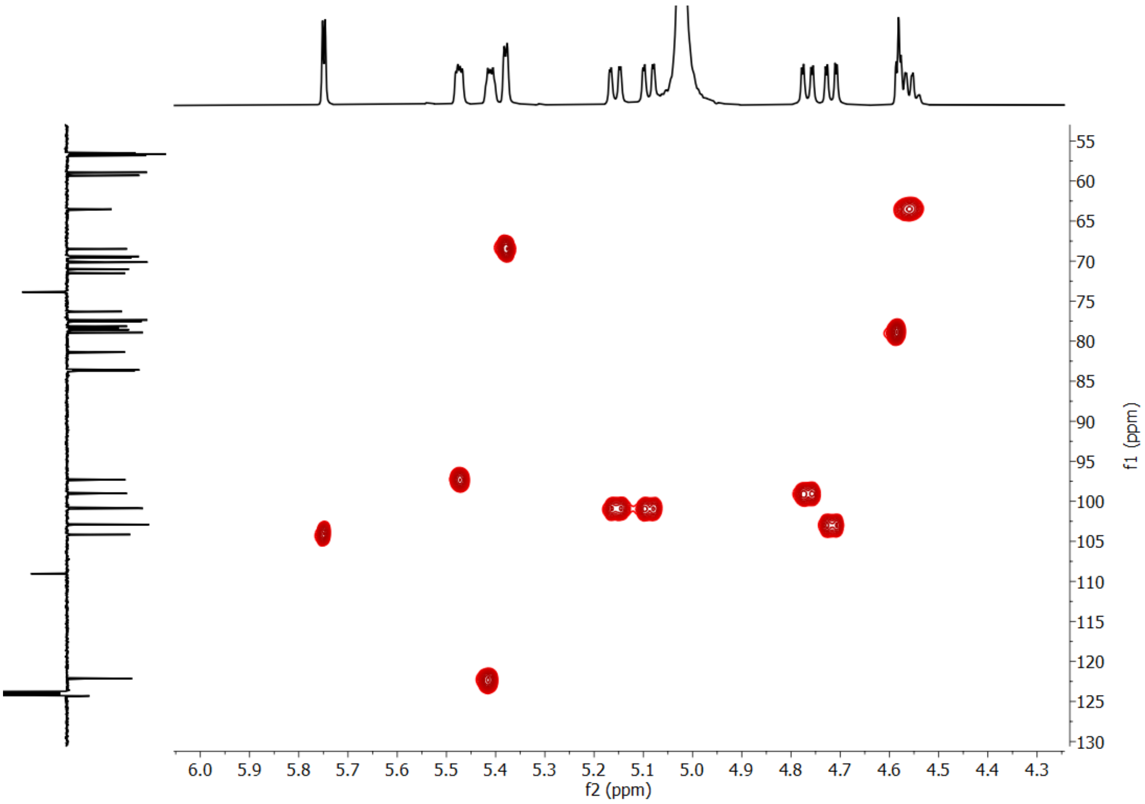

Figure S13: Expansion of HSQC spectrum(500 and 125 MHz, pyridine-d_5) of compound *1*.

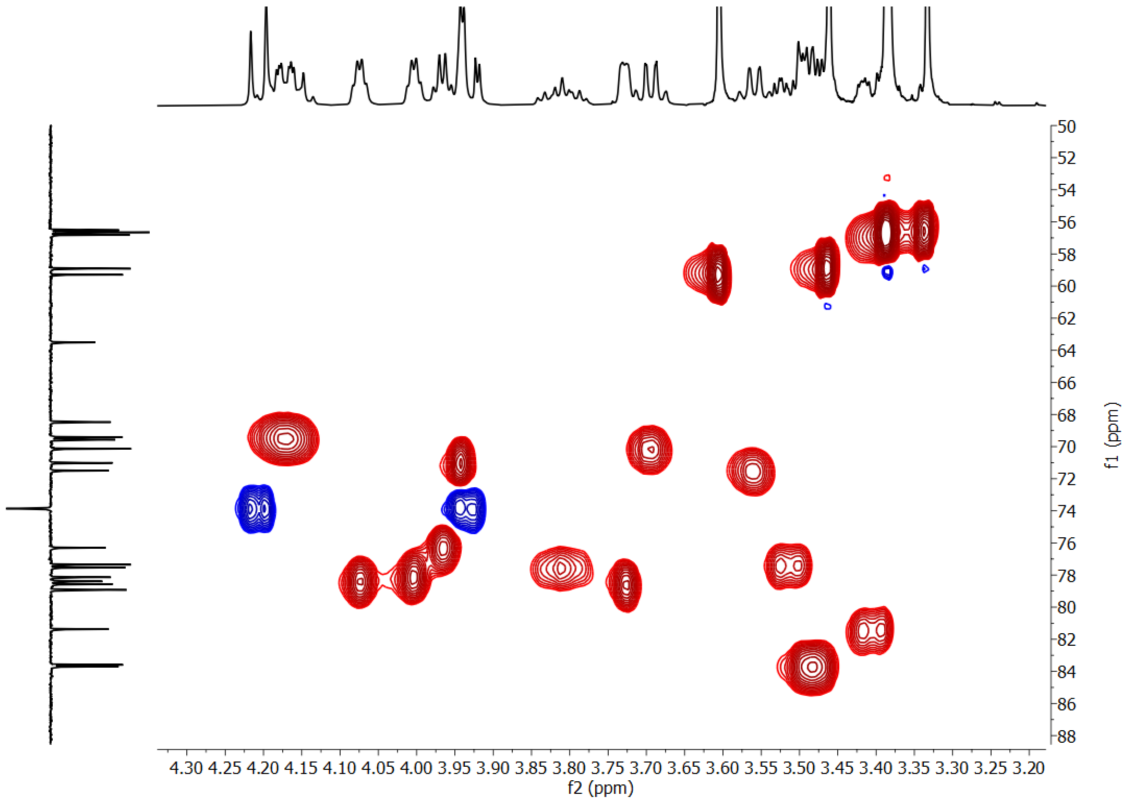

Figure S14: Expansion of HSQC spectrum(500 and 125 MHz, pyridine-d5) of compound *1*.

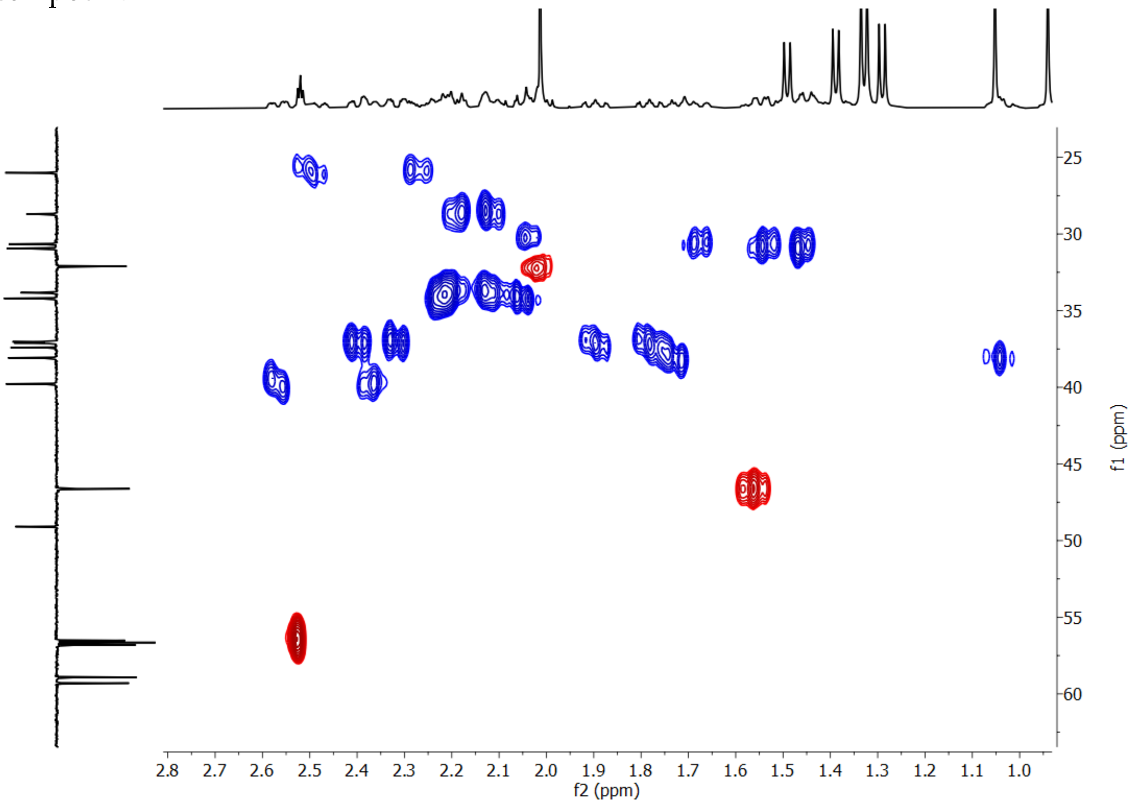

Figure S15: Expansion of HSQC spectrum(500 and 125 MHz, pyridine-d5) of compound *1*.

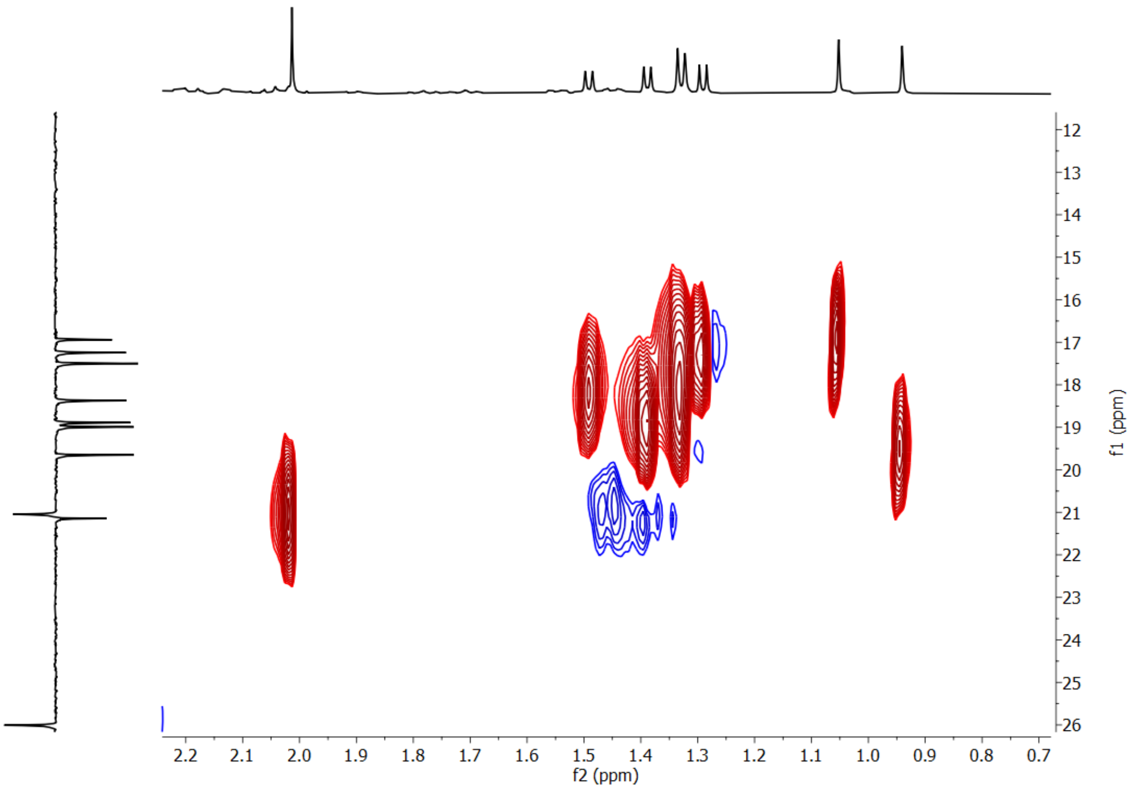

Figure S16: Expansion of HMBC spectrum(500 and 125 MHz, pyridine-d5) of compound *1*.

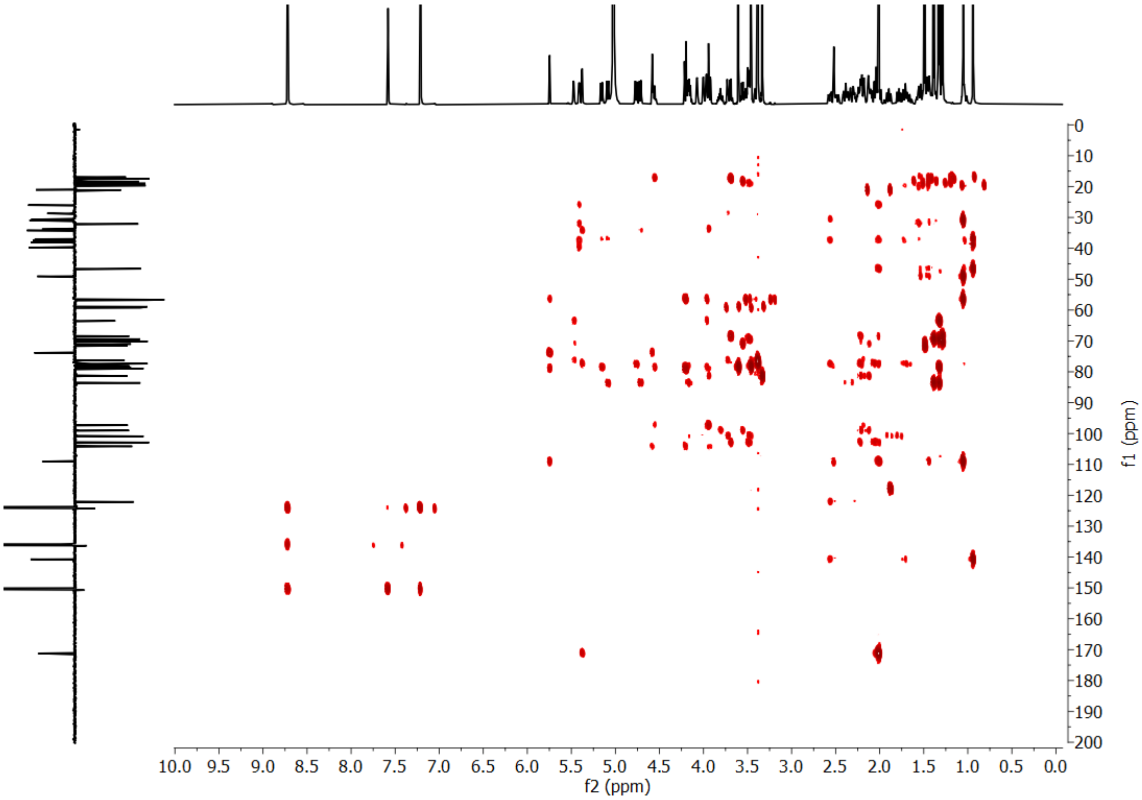

Figure S17: Expansion of HMBC spectrum(500 and 125 MHz, pyridine-d5) of compound *1*.

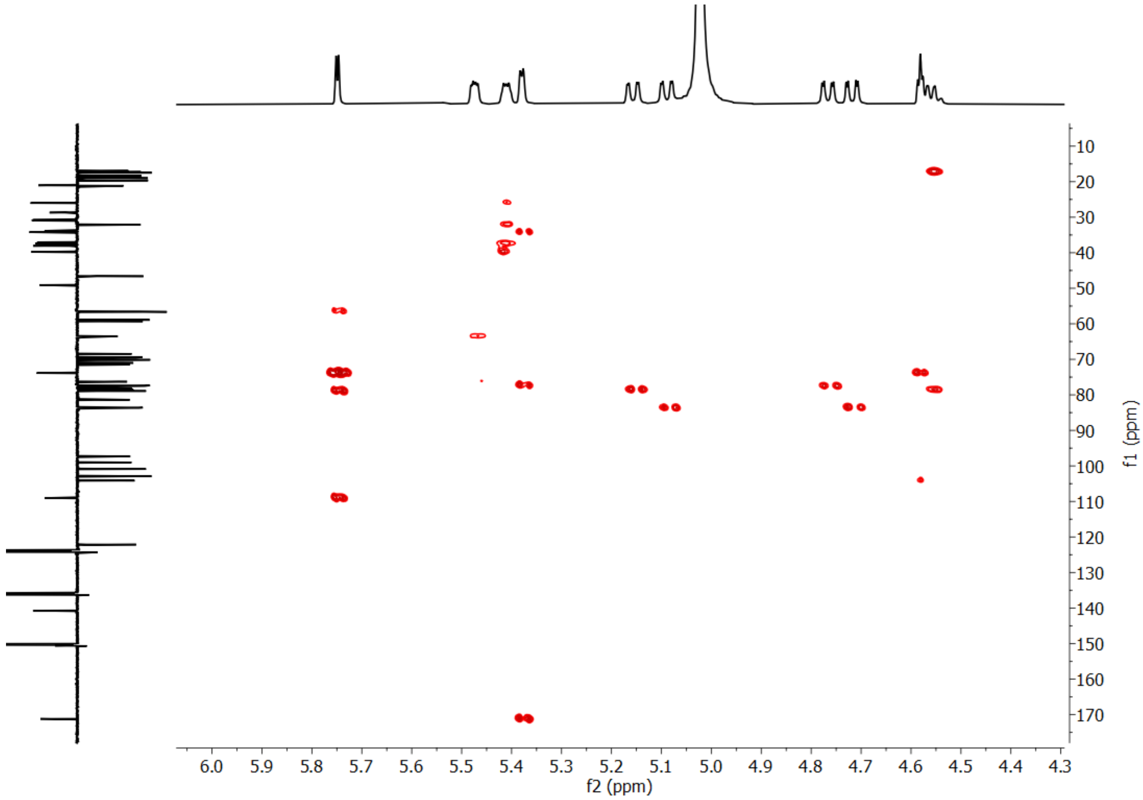

Figure S18: Expansion of HMBC spectrum(500 and 125 MHz, pyridine-d_5) of compound ***1***.

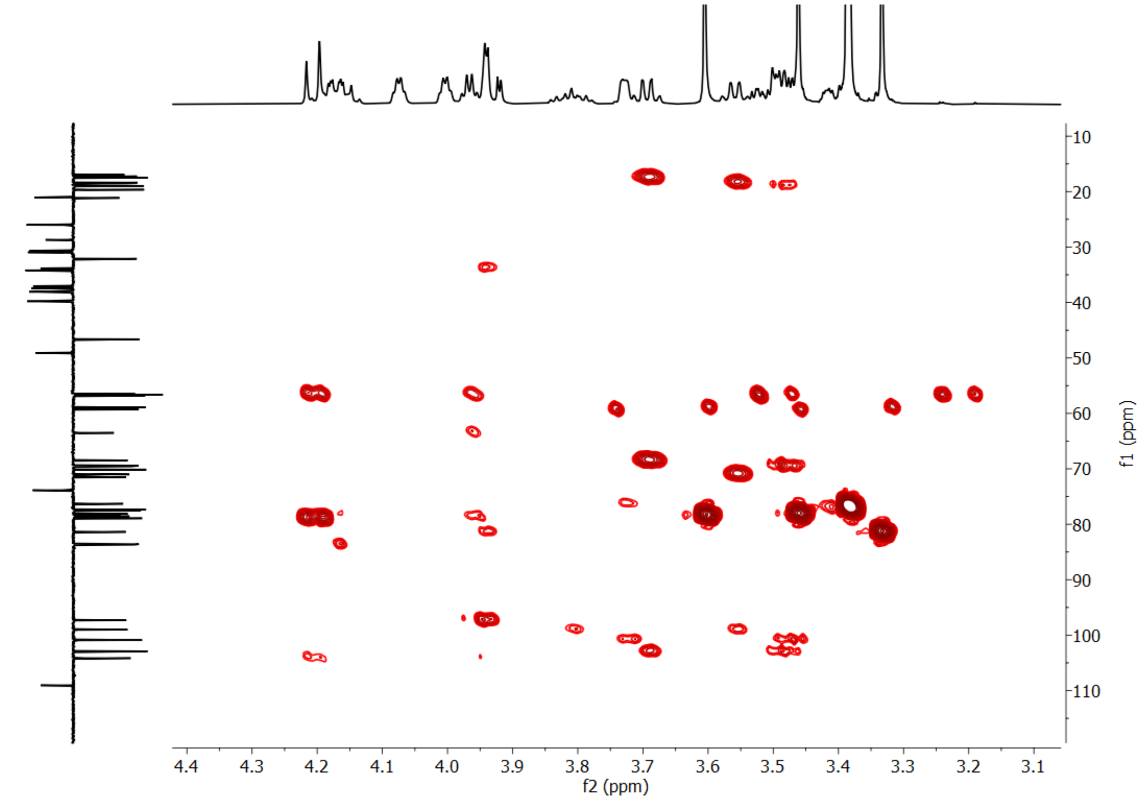

Figure S19: Expansion of HMBC spectrum(500 and 125 MHz, pyridine-d_5) of compound ***1***.

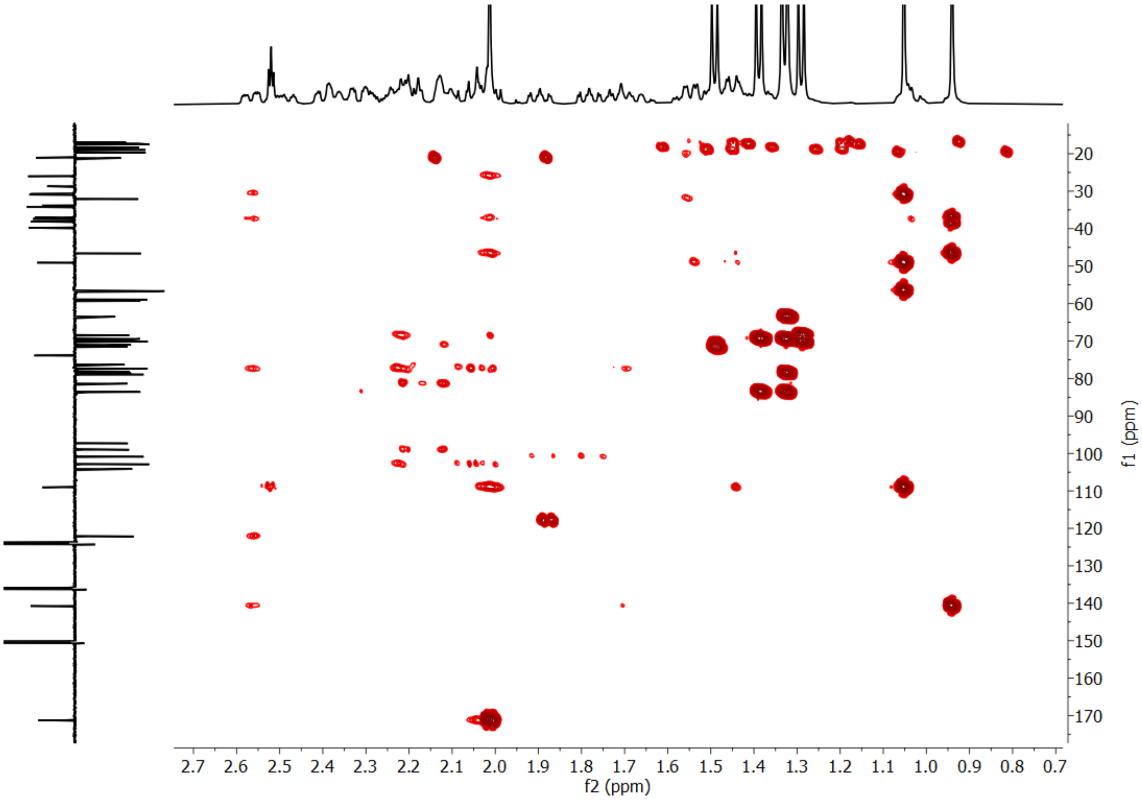

Figure S20: COSY spectrum (pyridine-d5, 500 MHz)of compound ***1***.

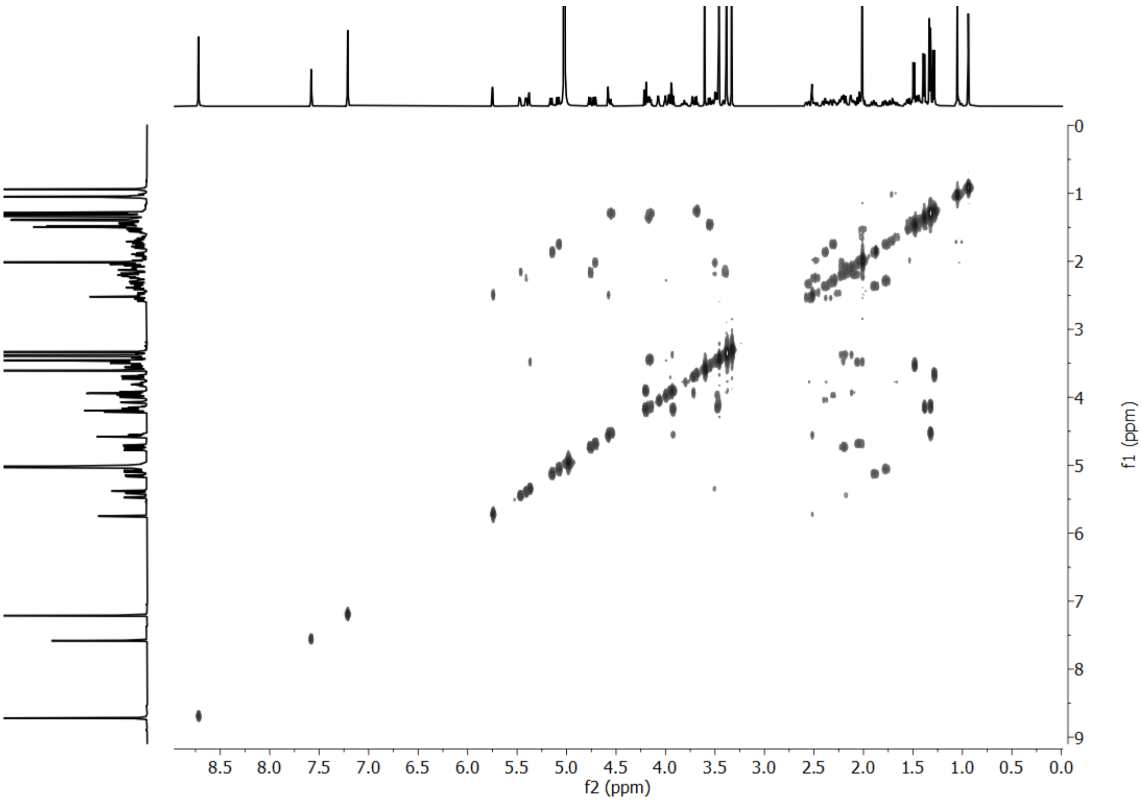

Figure S21: NOESY spectrum (pyridine-d5, 500 MHz) of compound ***1*.**

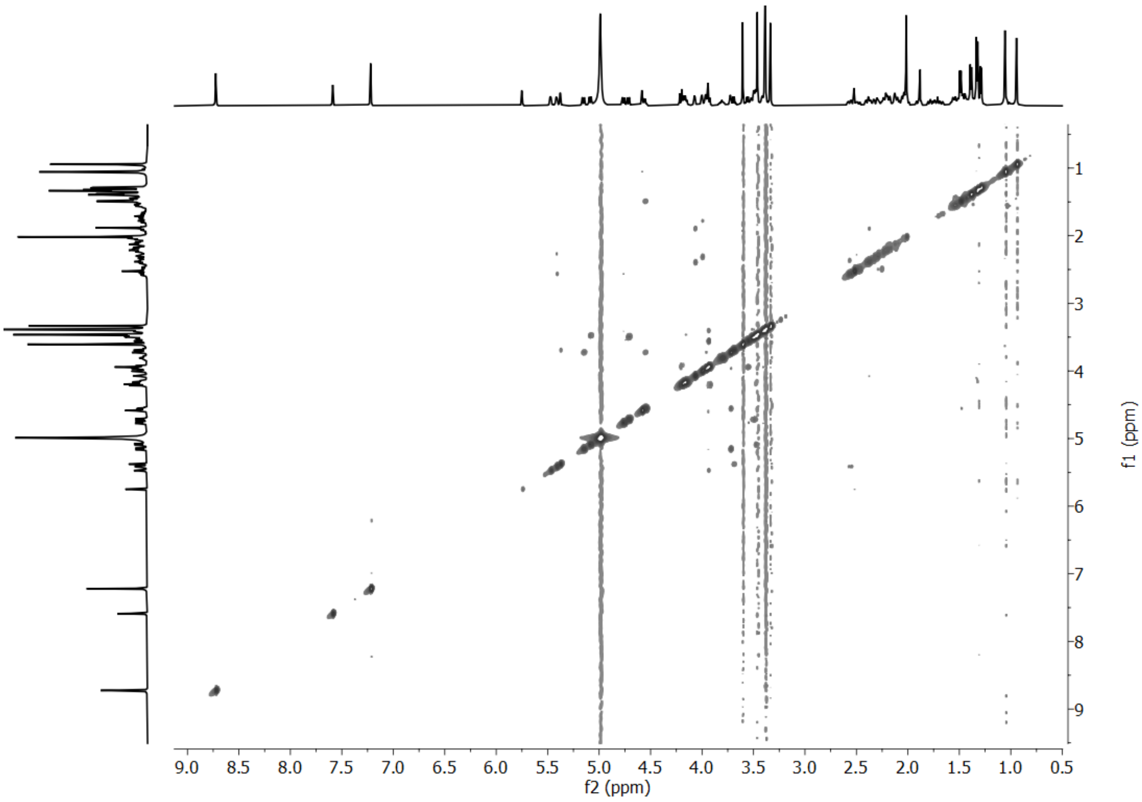

Figure S22: TOCSY spectrum (pyridine-d5, 500 MHz) of compound ***1.***

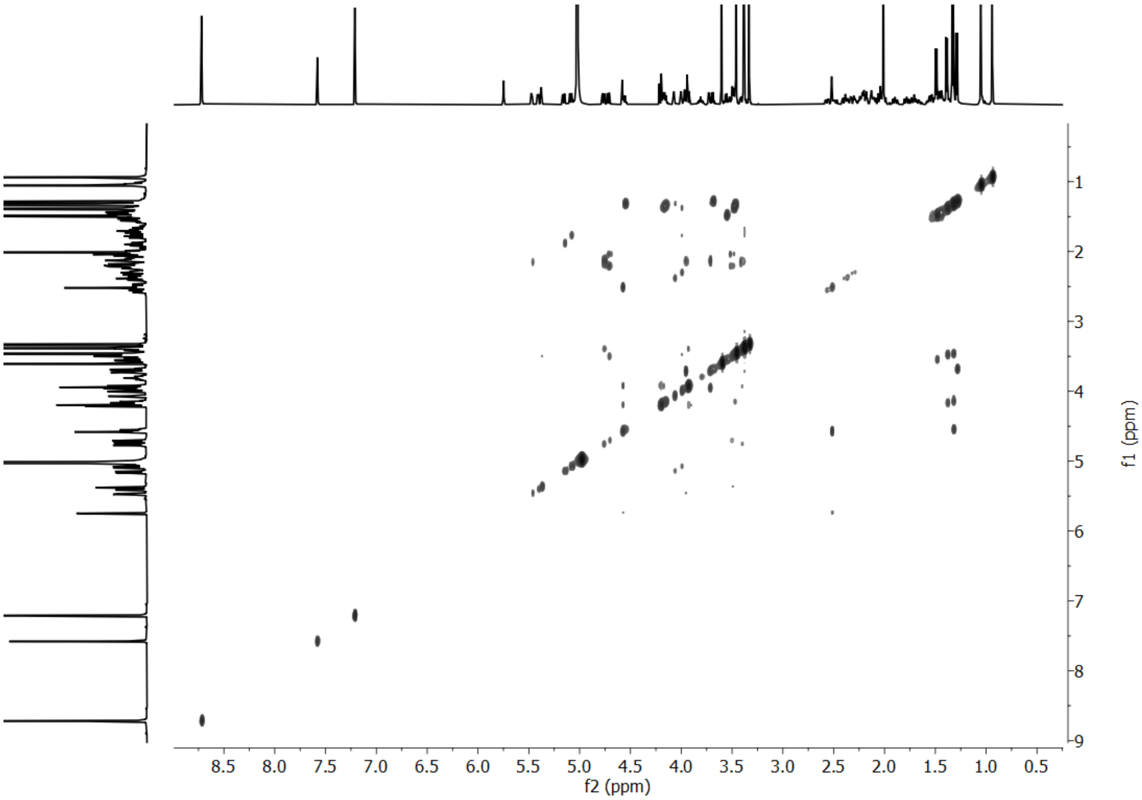

Figure S23: ESI-HRMS spectrum of compound **2**

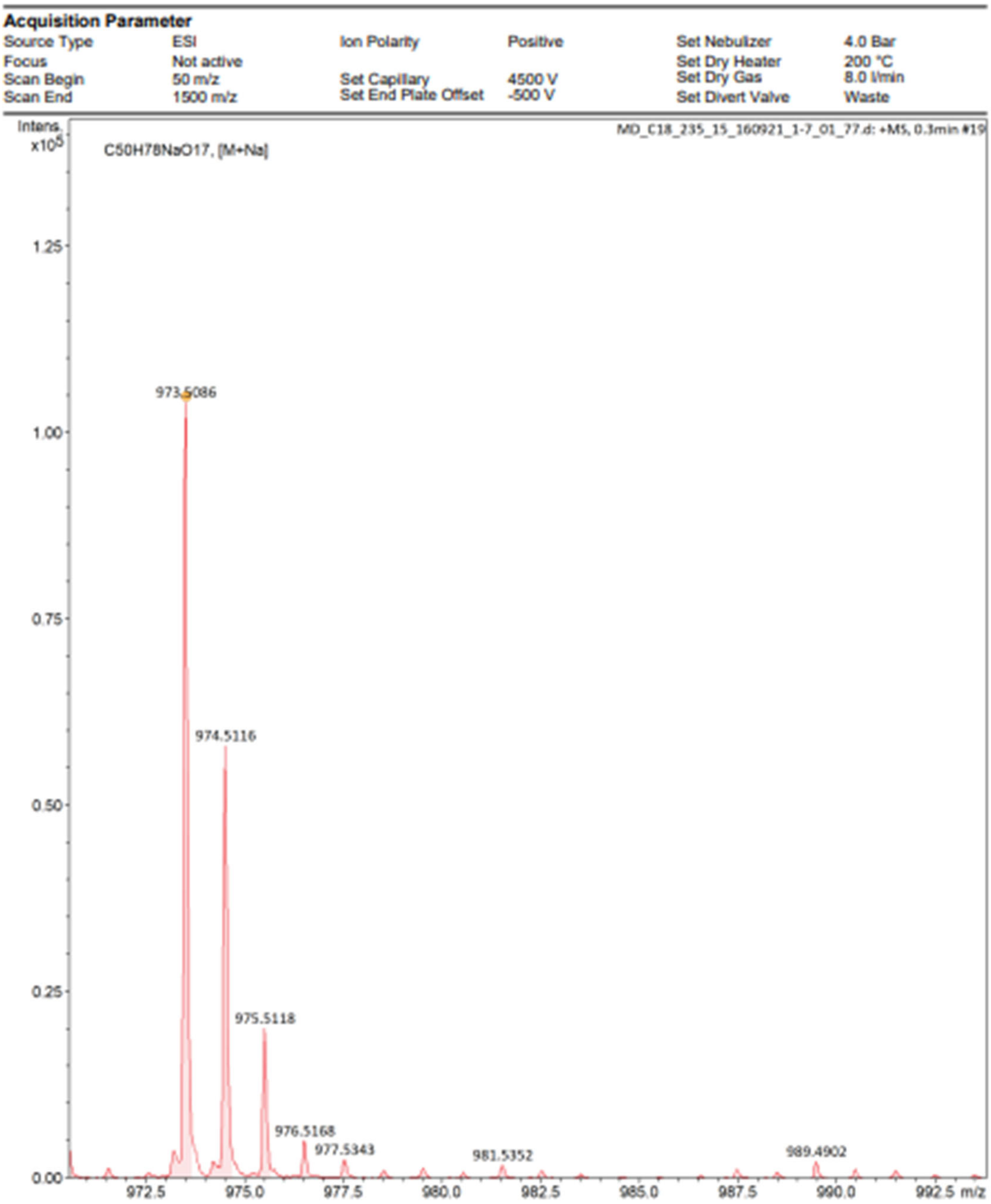

Figure S24: ^{1}H NMR spectrum (pyridine-d_5, 500 MHz) of compound **2.**

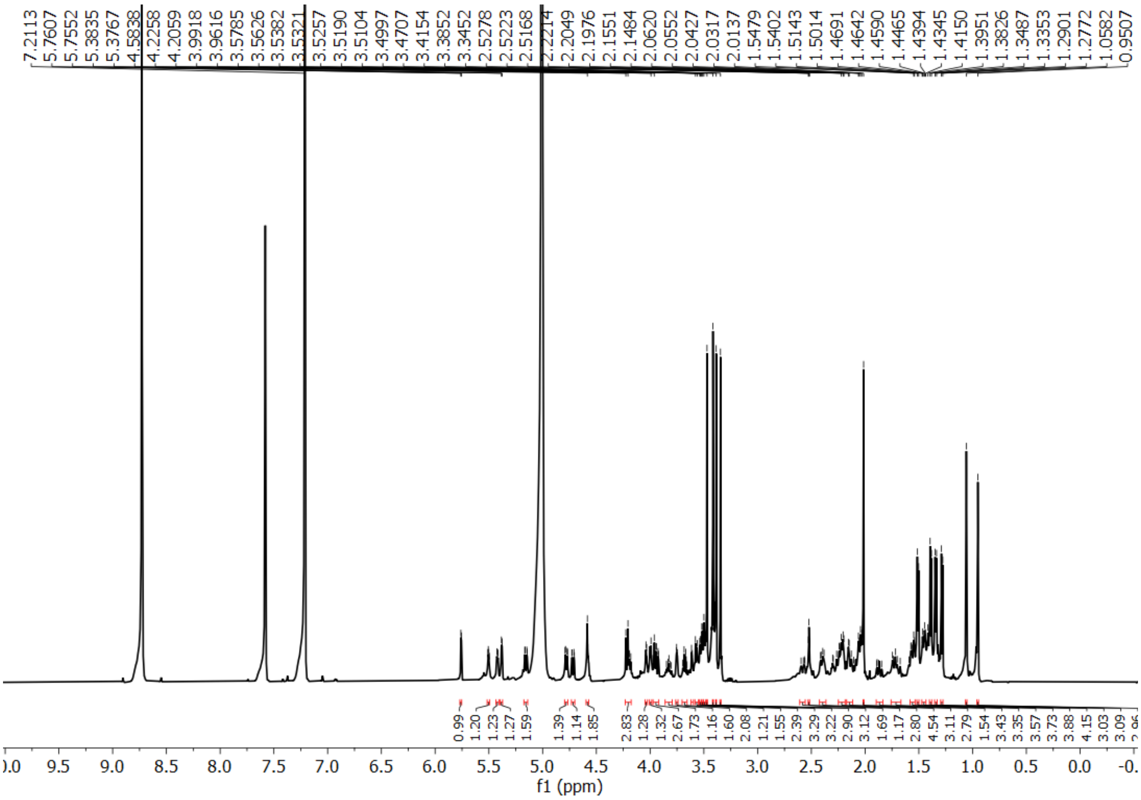

Figure S25: Expansion of ^{1}H NMR spectrum (pyridine-d_5, 500 MHz) of compound **2.**

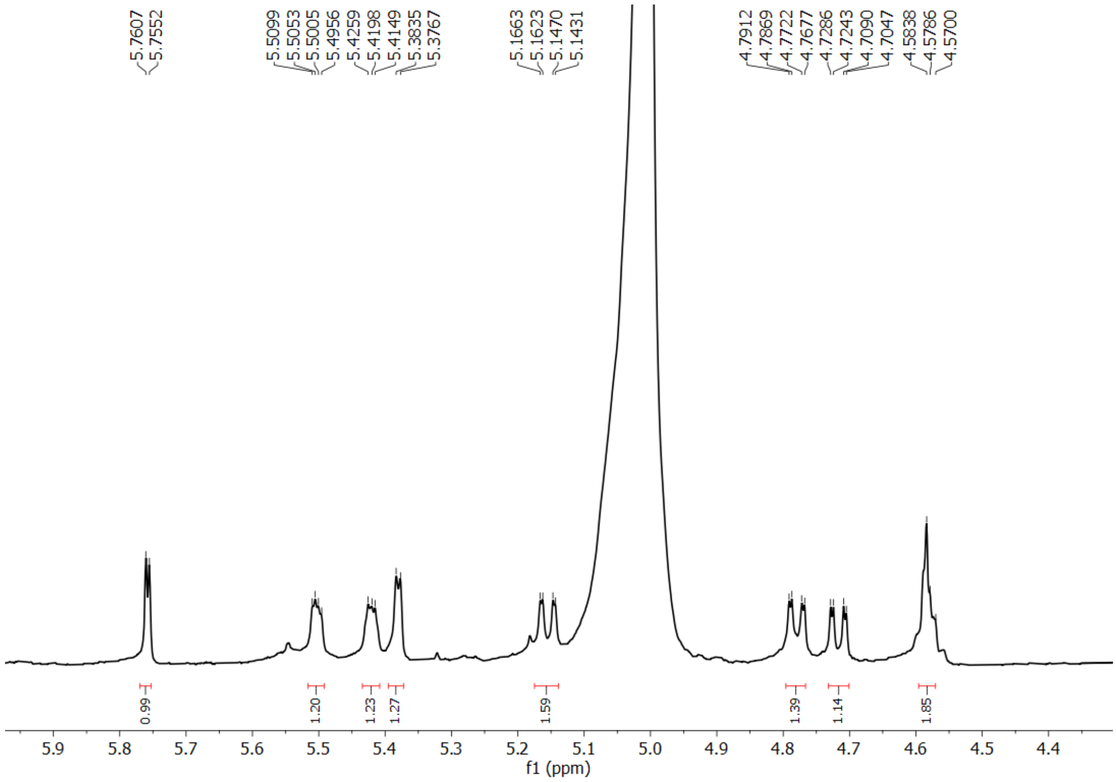

Figure S26: Expansion of ^{1}H NMR spectrum (pyridine-d5, 500 MHz) of compound **2.**

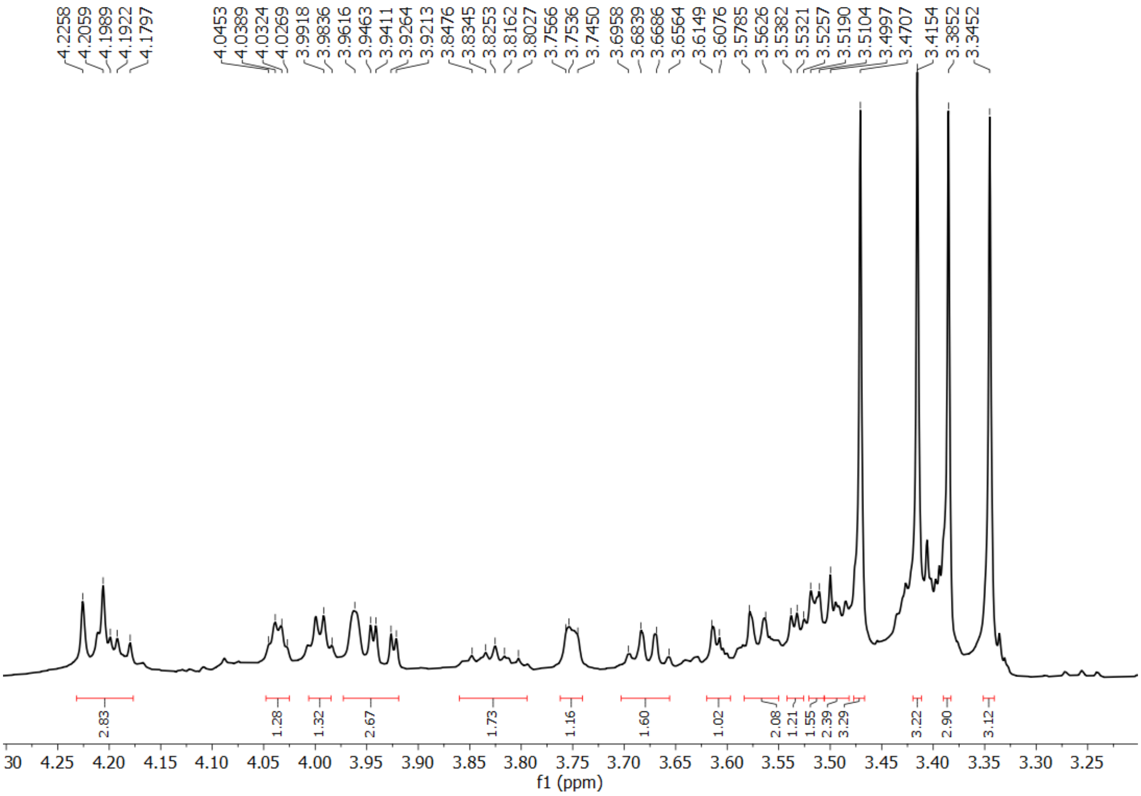

Figure S27: Expansion of ^{1}H NMR spectrum (pyridine-d5, 500 MHz) of compound **2.**

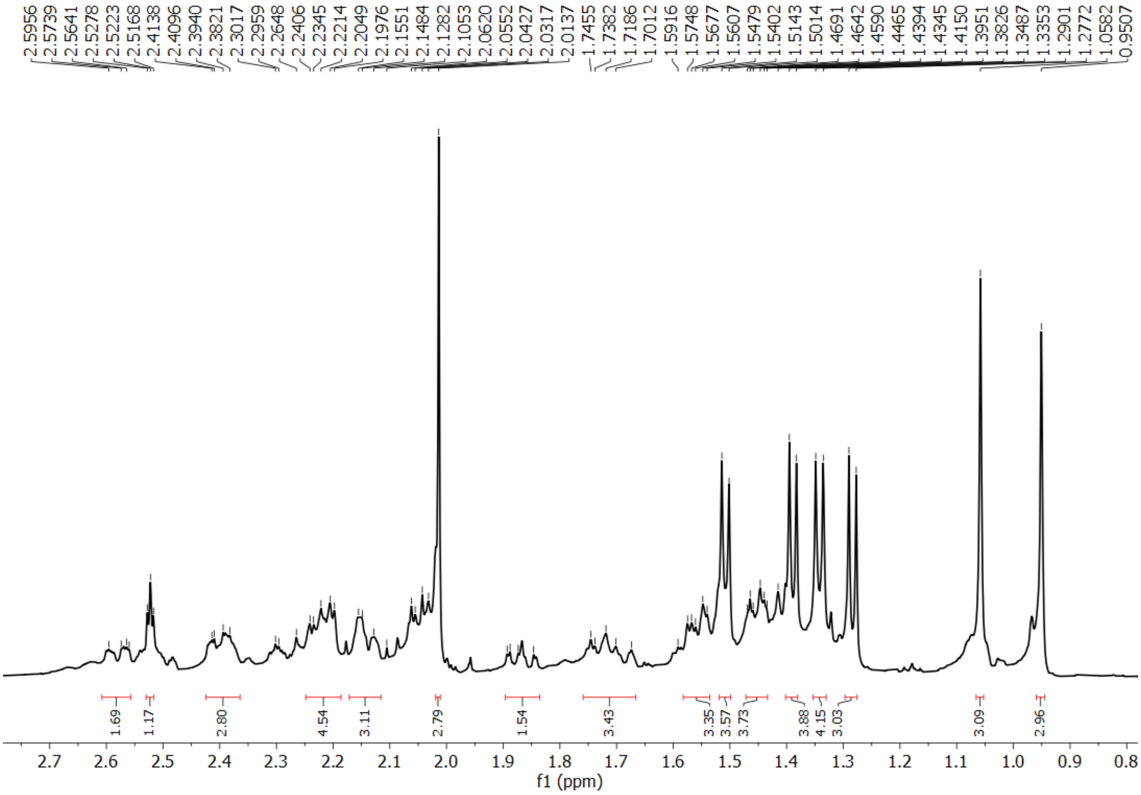

Figure S28: Dept 135 NMR spectrum (pyridine-d5, 500 MHz) of compound **2.**

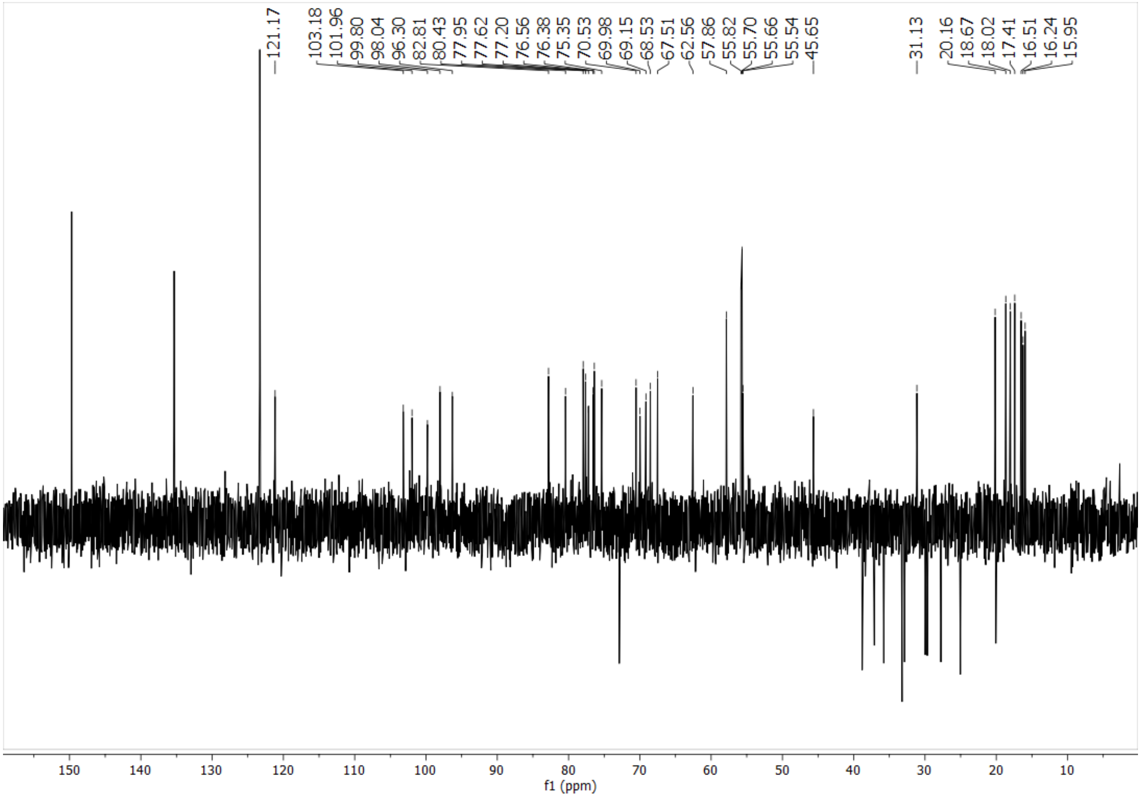

Figure S29: Expansion of Dept 135 NMR spectrum (pyridine-d5, 500 MHz) of compound **2.**

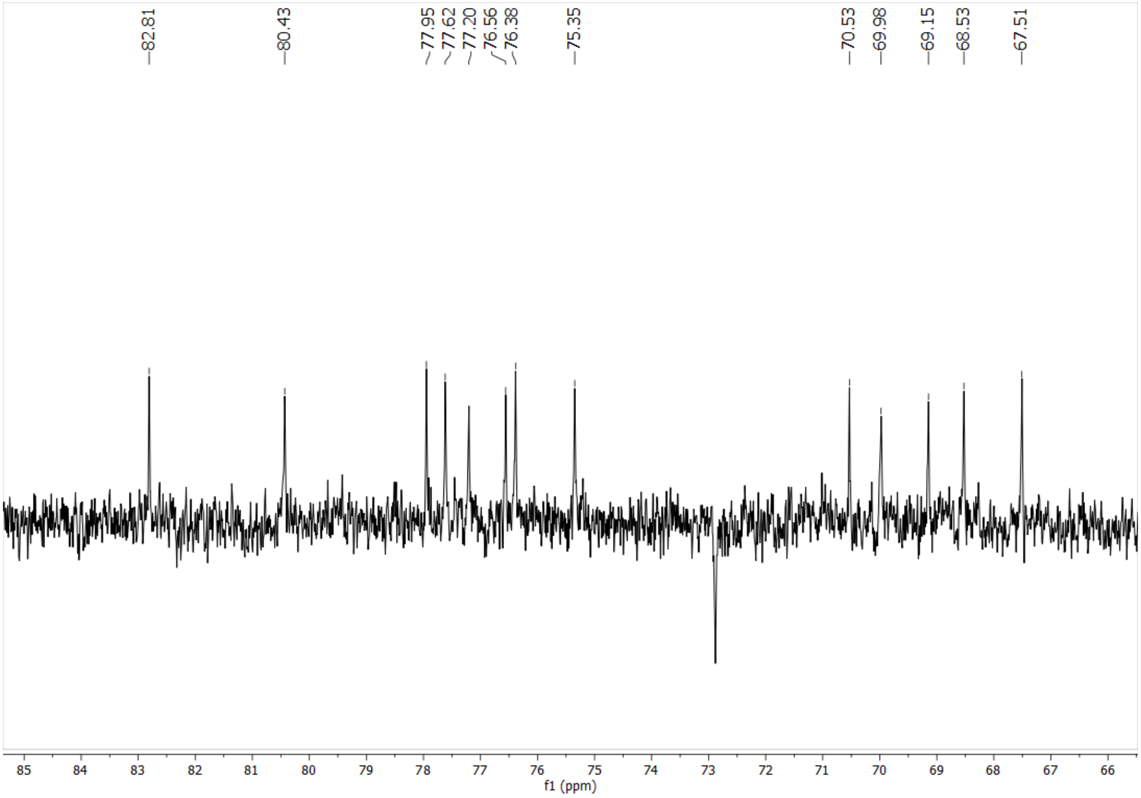

Figure S30: Expansion of Dept 135 NMR spectrum (pyridine-d5, 500 MHz) of compound **2.**

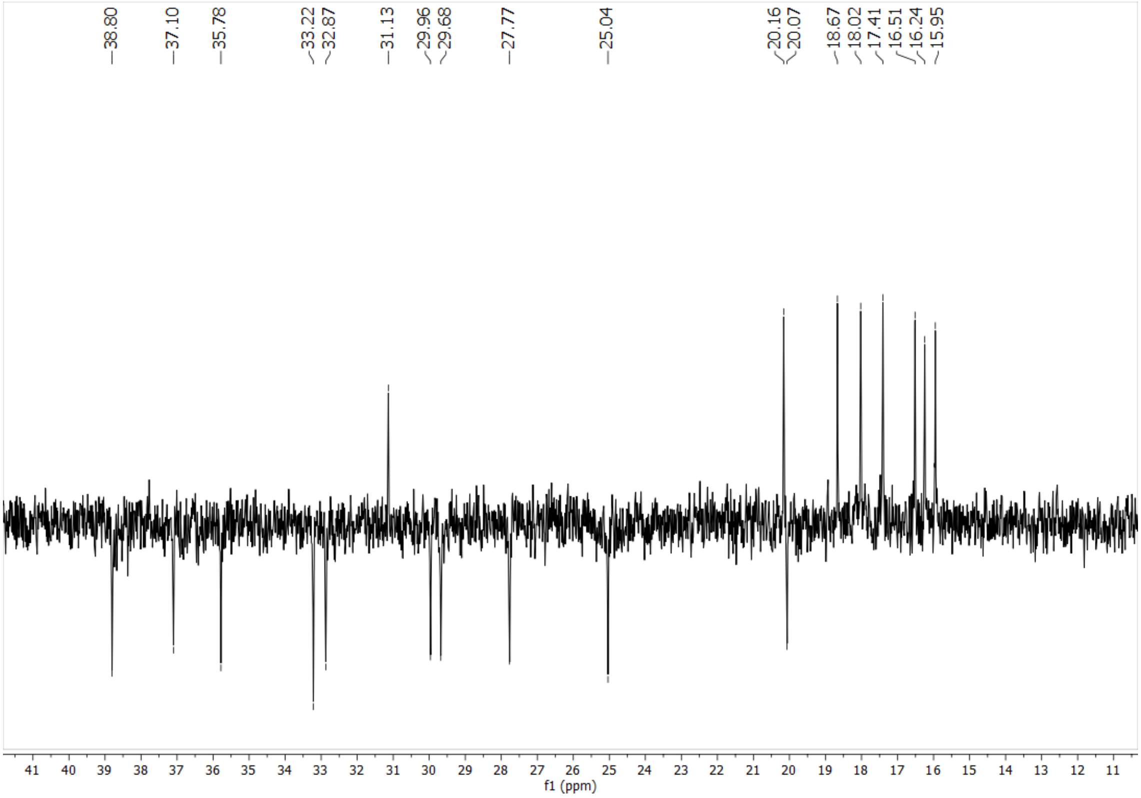

Figure S31: HSQC spectrum(500 and 125 MHz, pyridine-d5) of compound **2**.

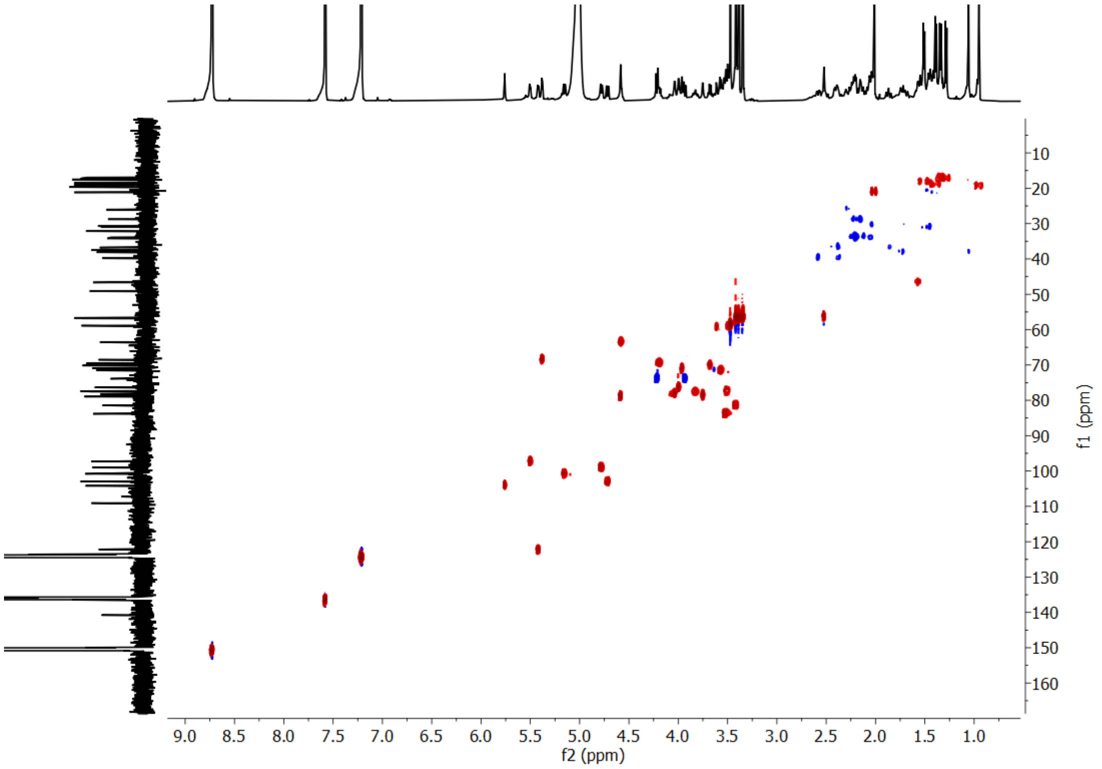

Figure S32: Expansion of HSQC spectrum(500 and 125 MHz, pyridine-d_5) of compound 2.

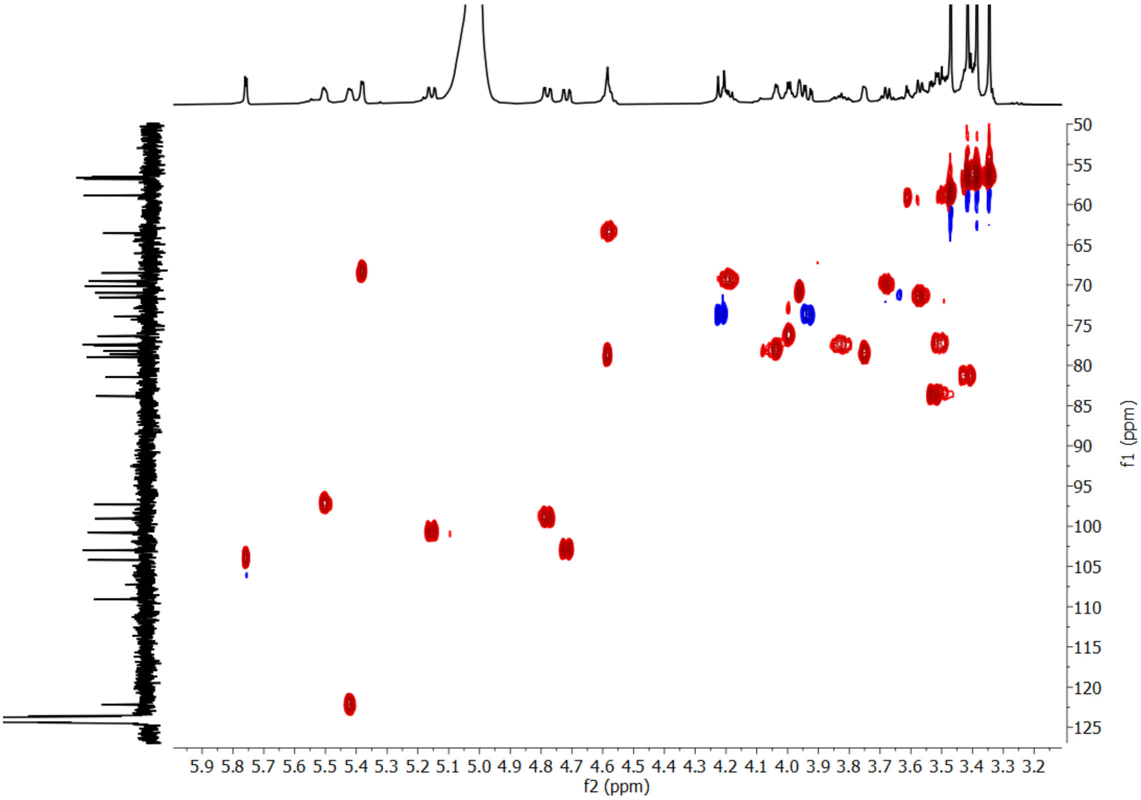

Figure S33: Expansion of HSQC spectrum(500 and 125 MHz, pyridine-d_5) of compound 2.

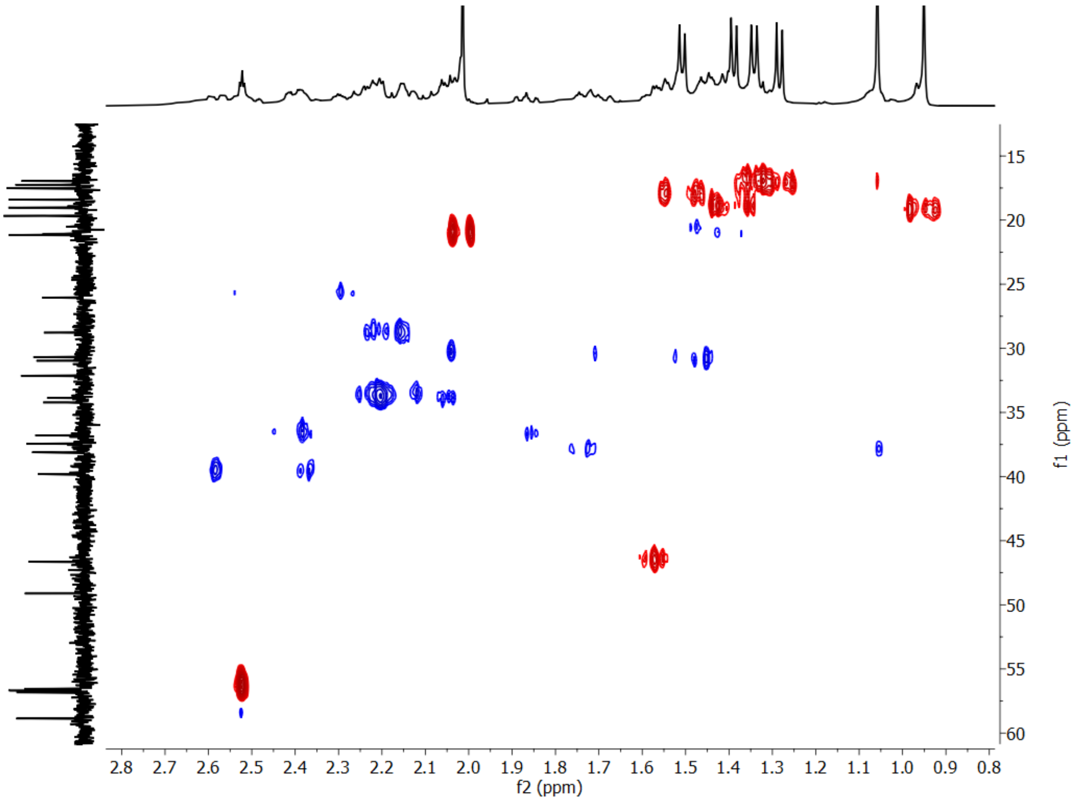

Figure S34: HMBC spectrum(500 and 125 MHz, pyridine-d_5) of compound **2**.

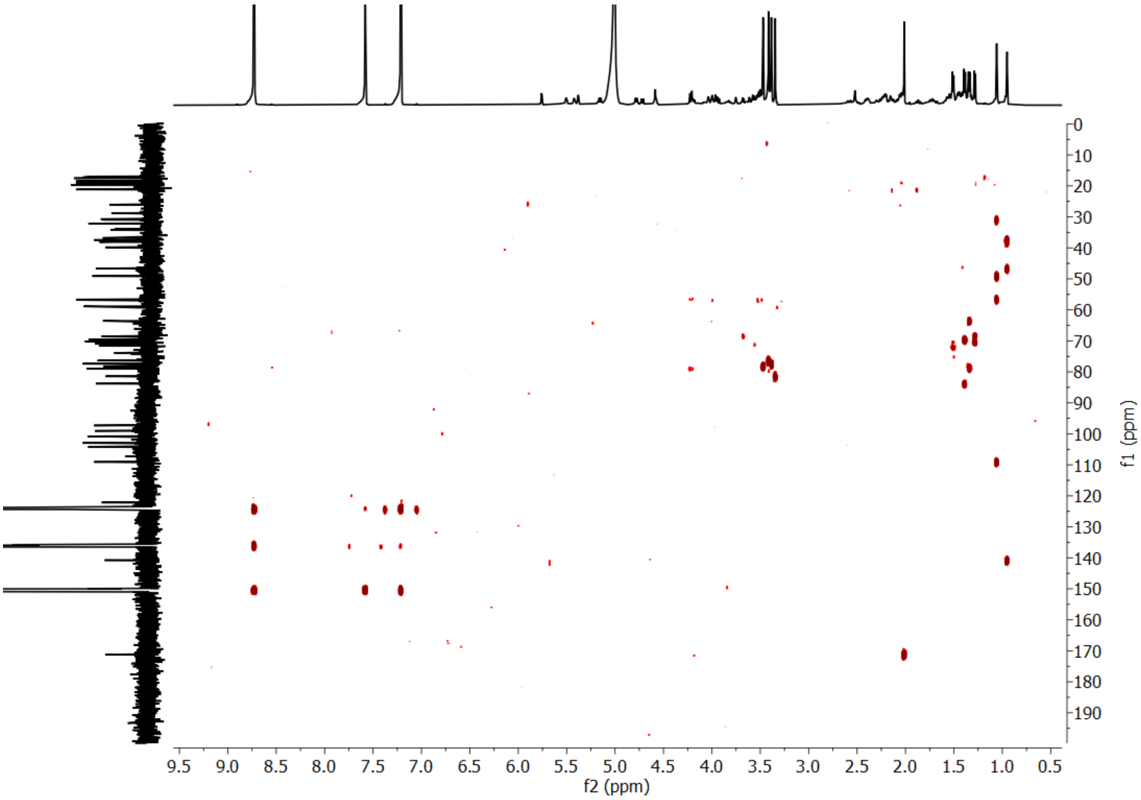

Figure S35: Expansion of HMBC spectrum(500 and 125 MHz, pyridine-d_5) of compound **2**.

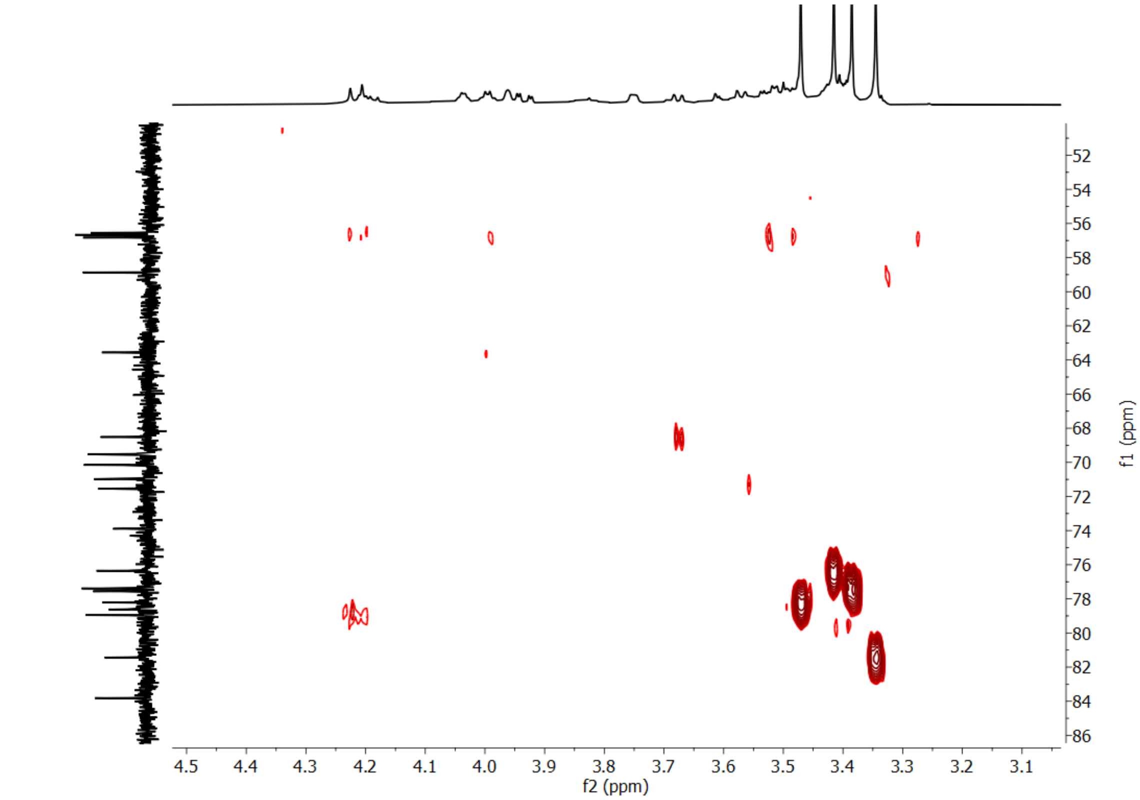

Figure S36: Expansion of HMBC spectrum(500 and 125 MHz, pyridine-d5) of compound **2**.

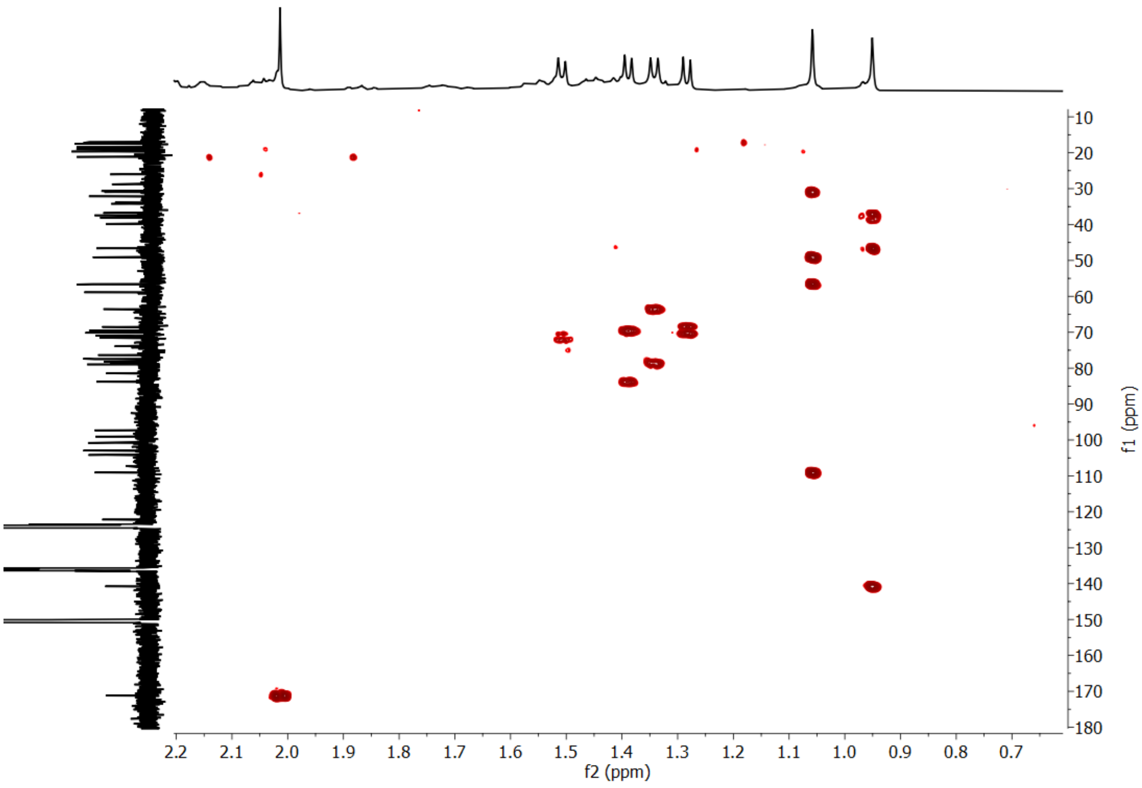

Figure S37: COSY spectrum (500 and 125 MHz, pyridine-d5) of compound **2**.

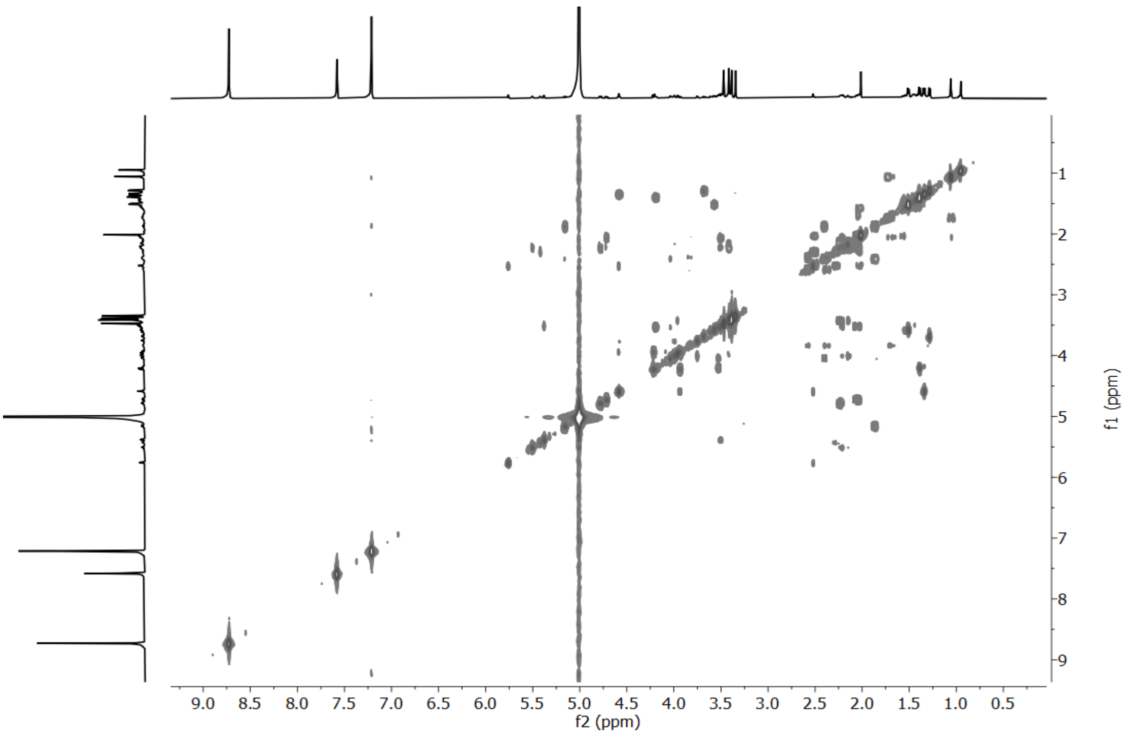

Figure S38: ESI-HRMS spectrum of compound *3*

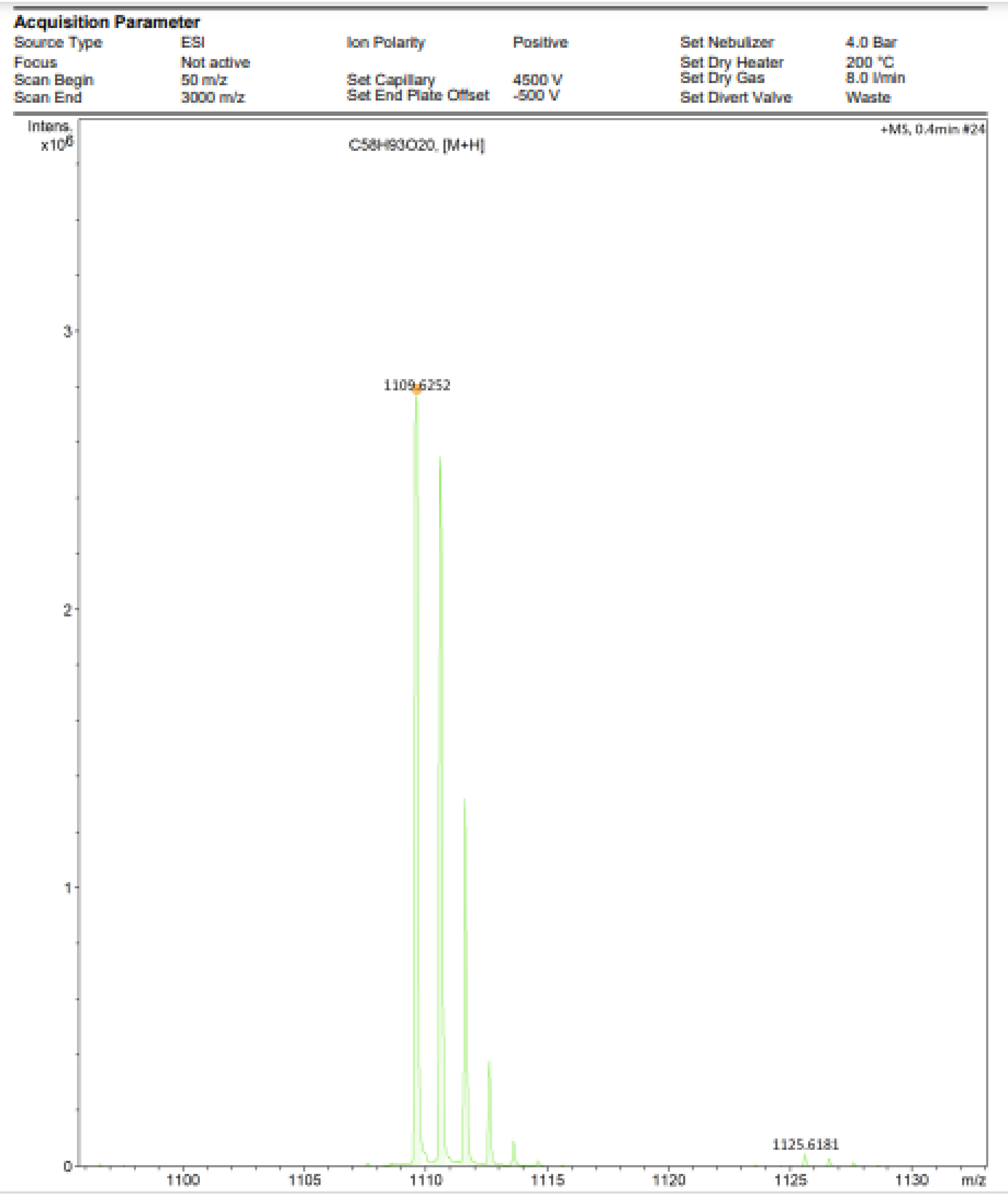

Figure S39: ^{1}H NMR spectrum (400 MHz, pyridine-d5) of compound ***3*.**

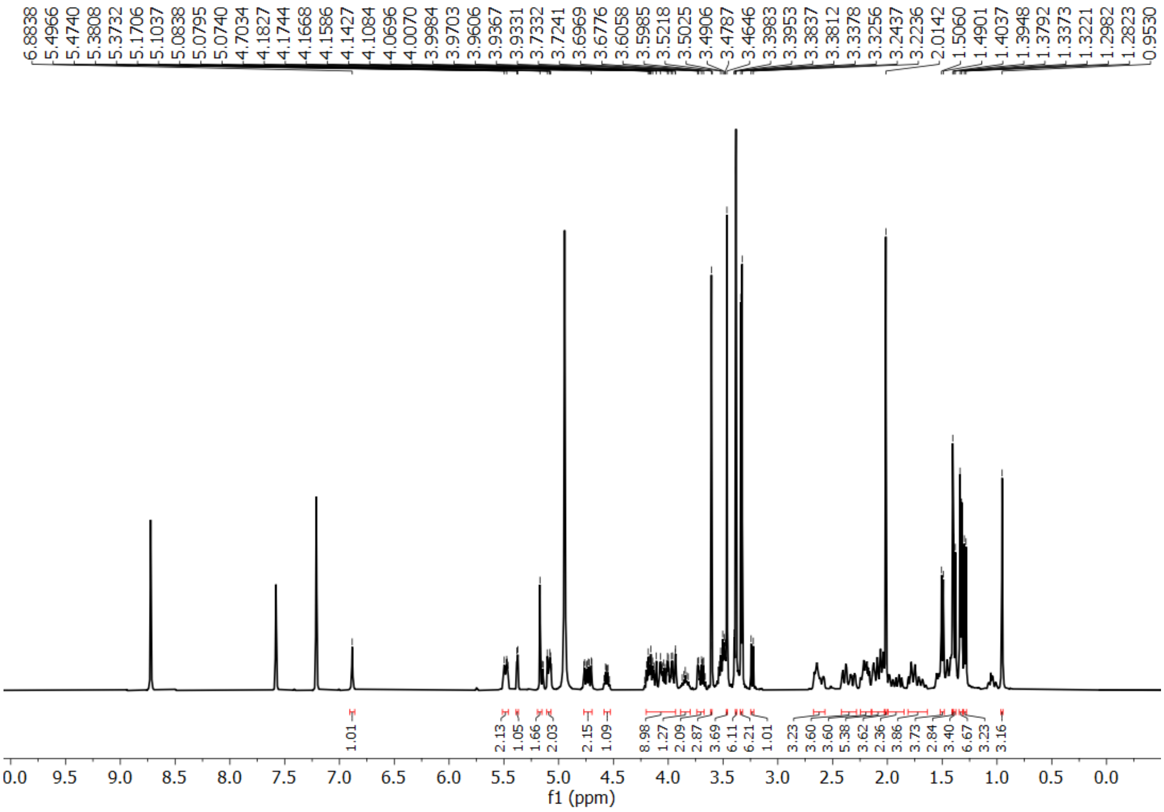

Figure S40: Expansion of ^{1}H NMR spectrum (400 MHz, pyridine-d5) of compound ***3*.**

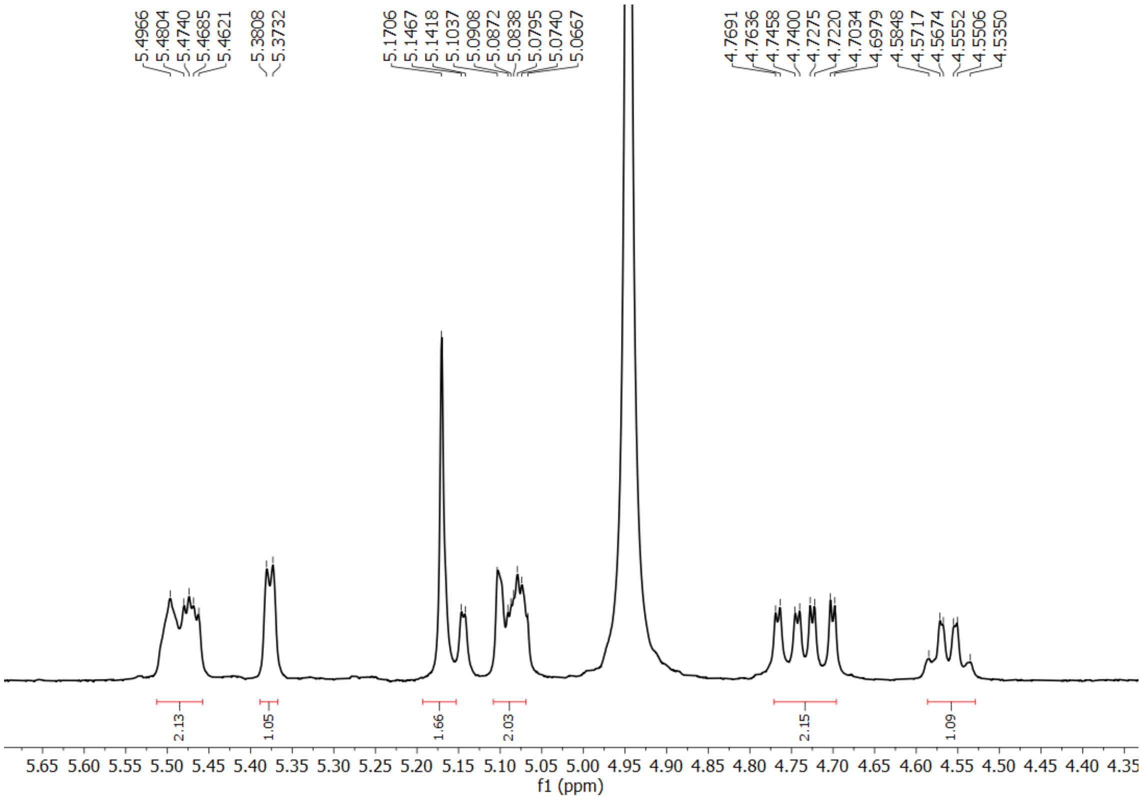

Figure S41: Expansion of ^{1}H NMR spectrum (400 MHz, pyridine-d5) of compound ***3.***

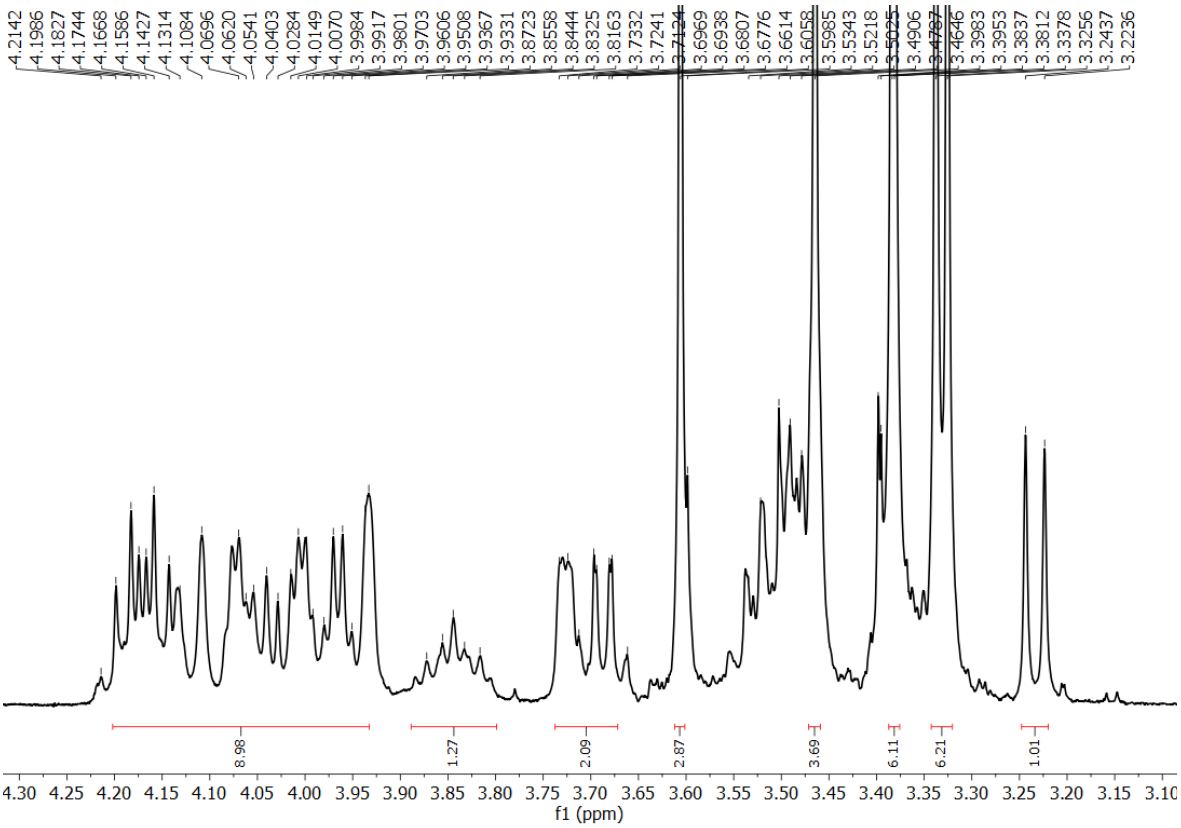

Figure S42: Expansion of ^{1}H NMR spectrum (400 MHz, pyridine-d5) of compound ***3.***

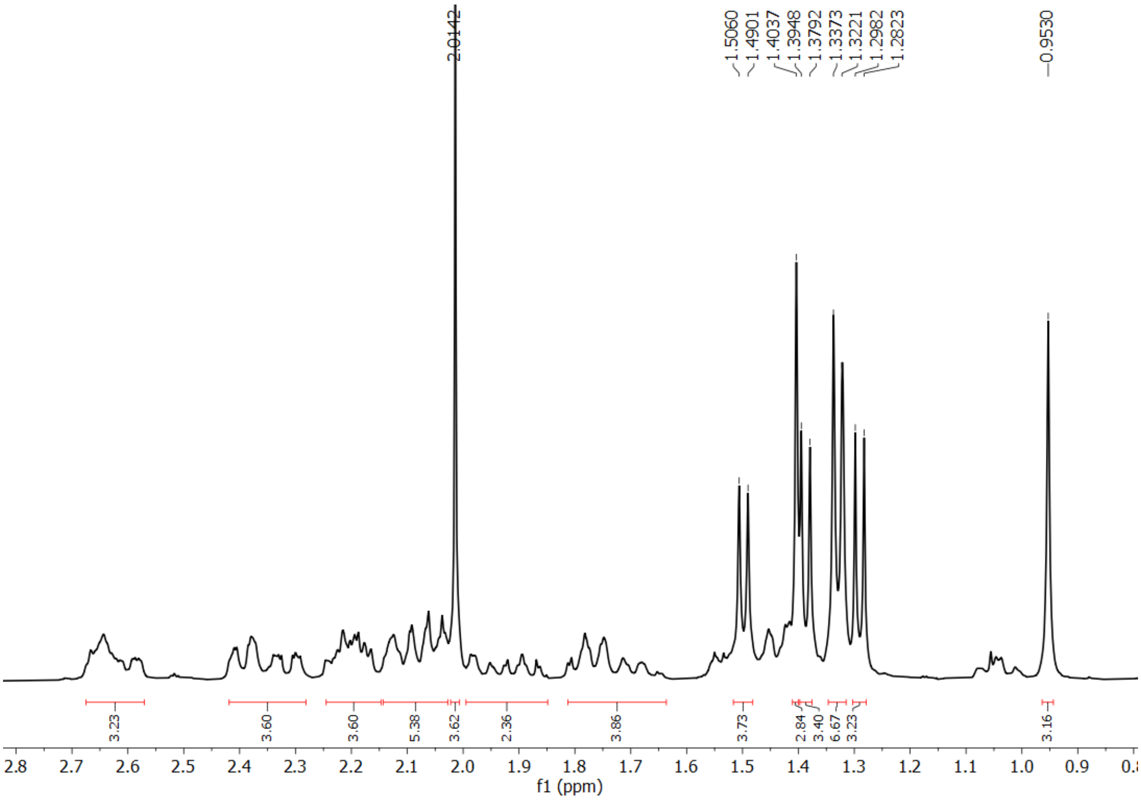

Figure S43: APT NMR spectrum (400 MHz, pyridine-d5) of compound *3*.

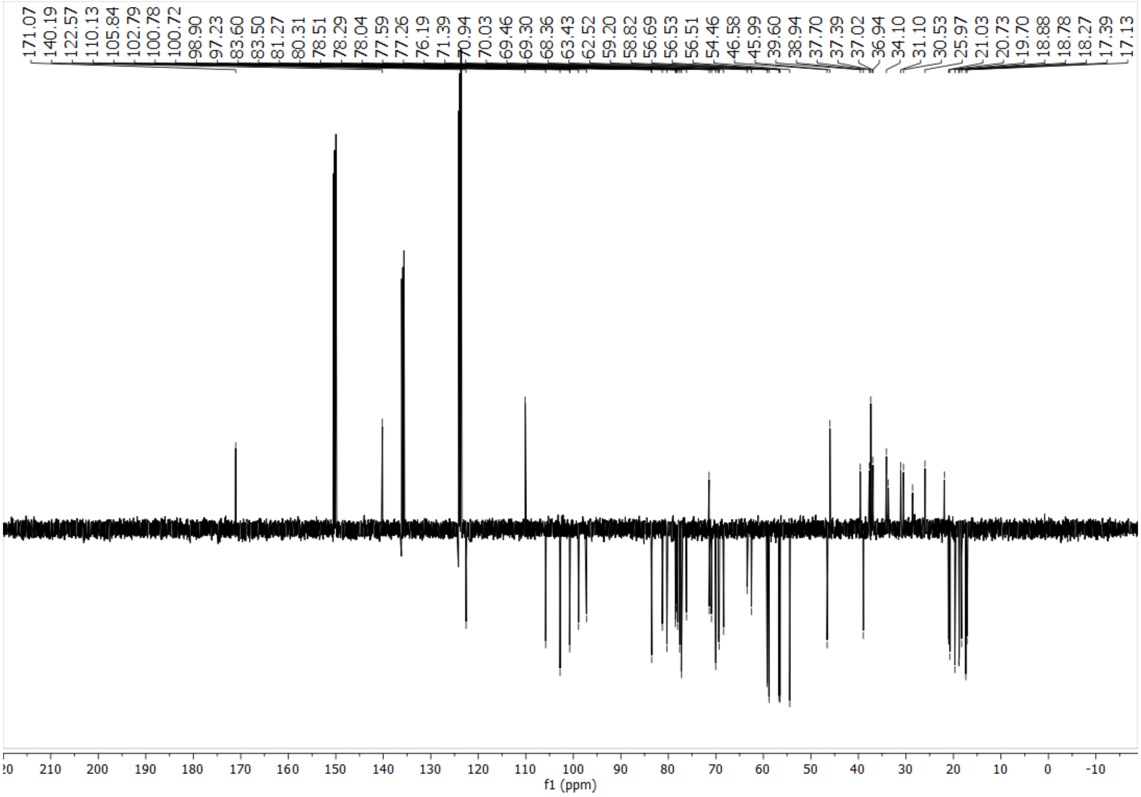

Figure S44: Expansion of APT NMR spectrum (400 MHz, pyridine-d5) of compound *3*.

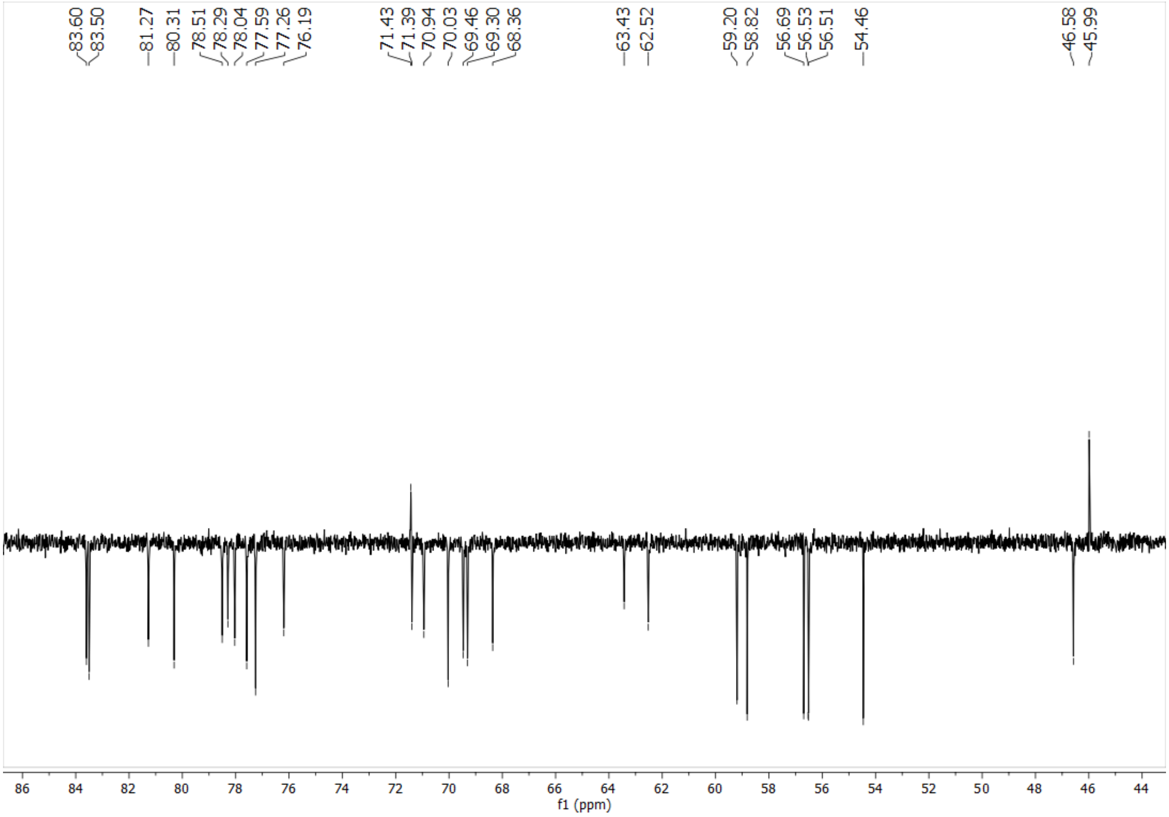

Figure S45: Expansion of APT NMR spectrum (400 MHz, pyridine-d5) of compound *3*.

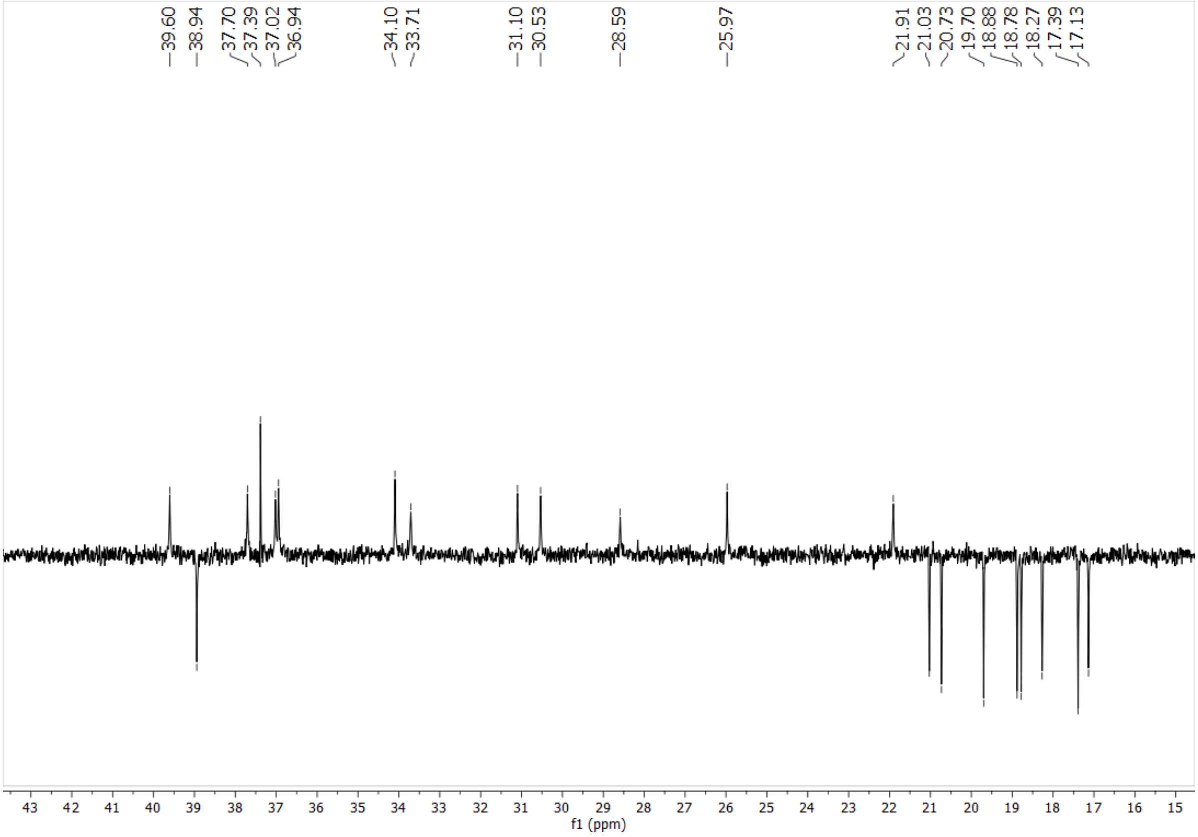

Figure S46: HSQC spectrum (400 and 100 MHz, pyridine-d_5) of compound *3*

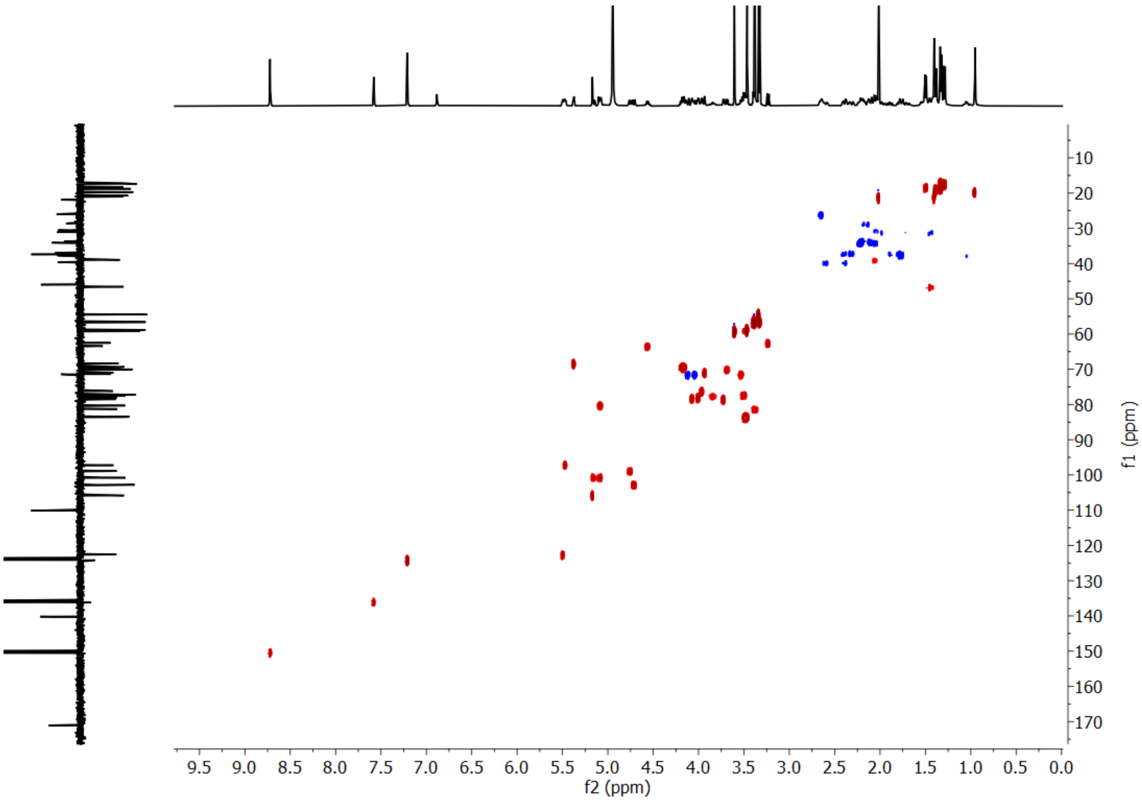

Figure S47: Expansion of HSQC spectrum (400 and 100 MHz, pyridine-d_5) of compound *3*

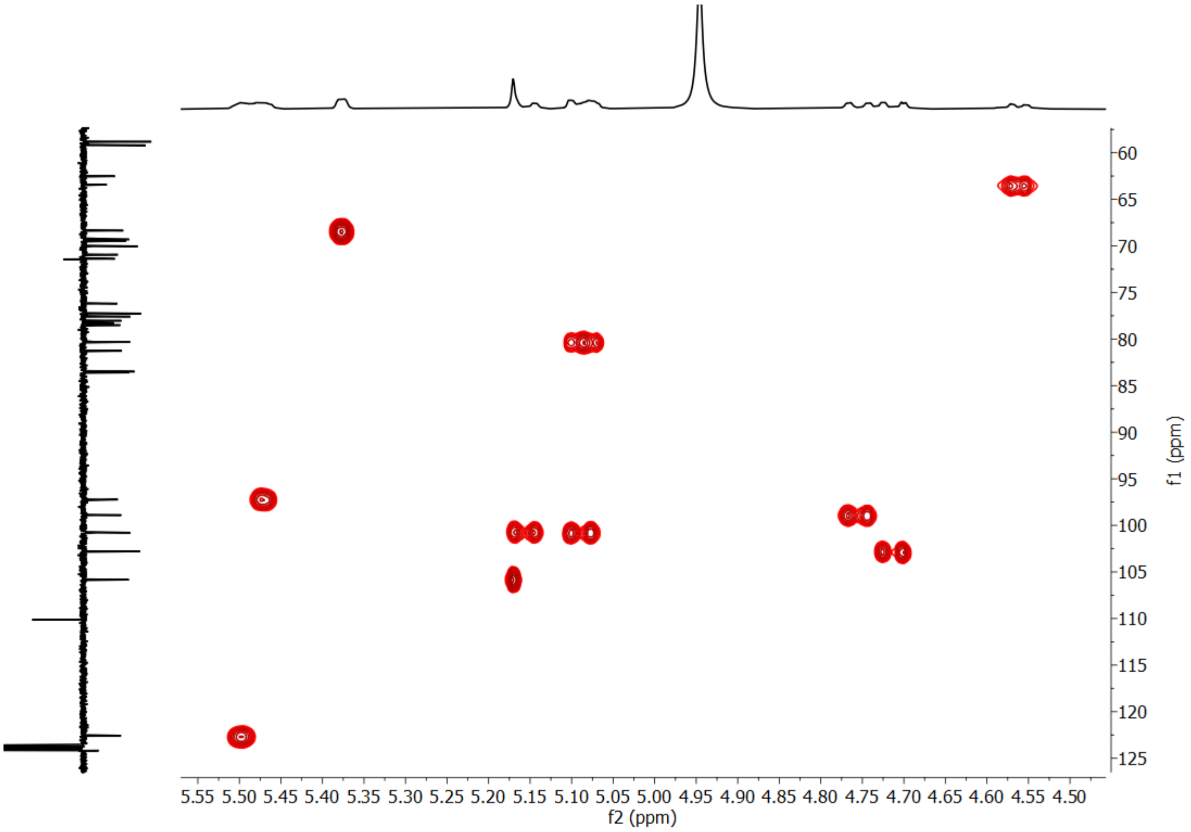

Figure S48: Expansion of HSQC spectrums (400 and 100 MHz, pyridine-d_5) of compound *3*

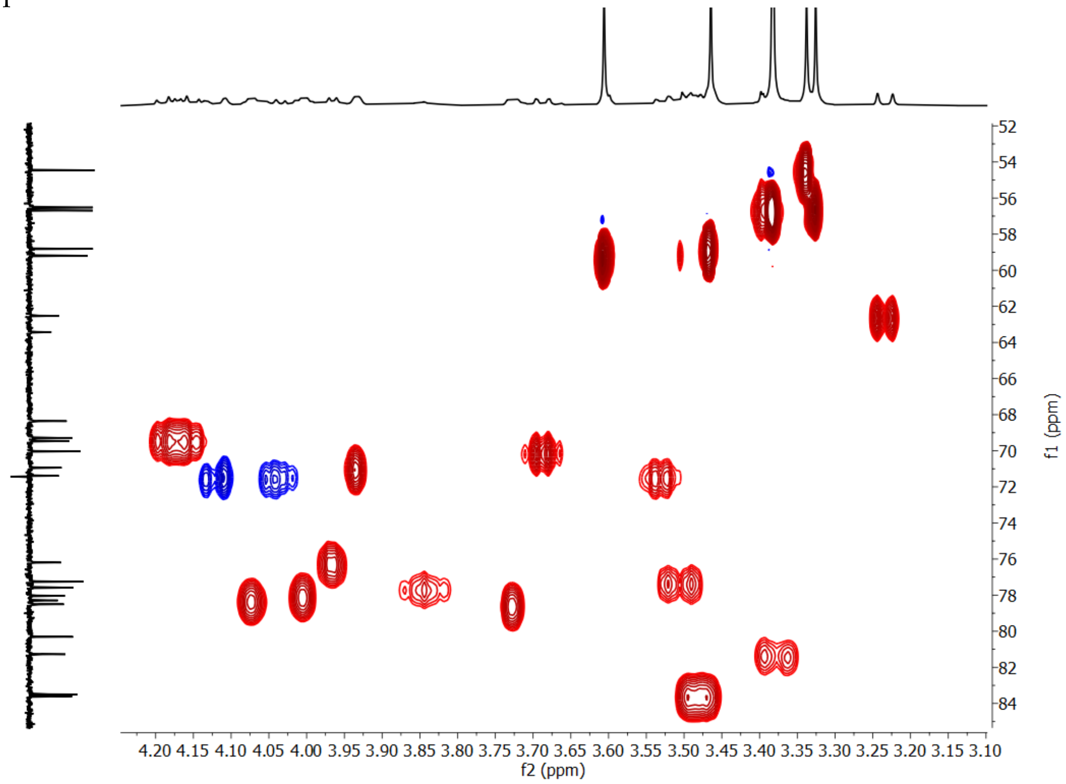

Figure S49: Expansion of HSQC spectrum(400 and 100 MHz, pyridine-d5) of compound *3*

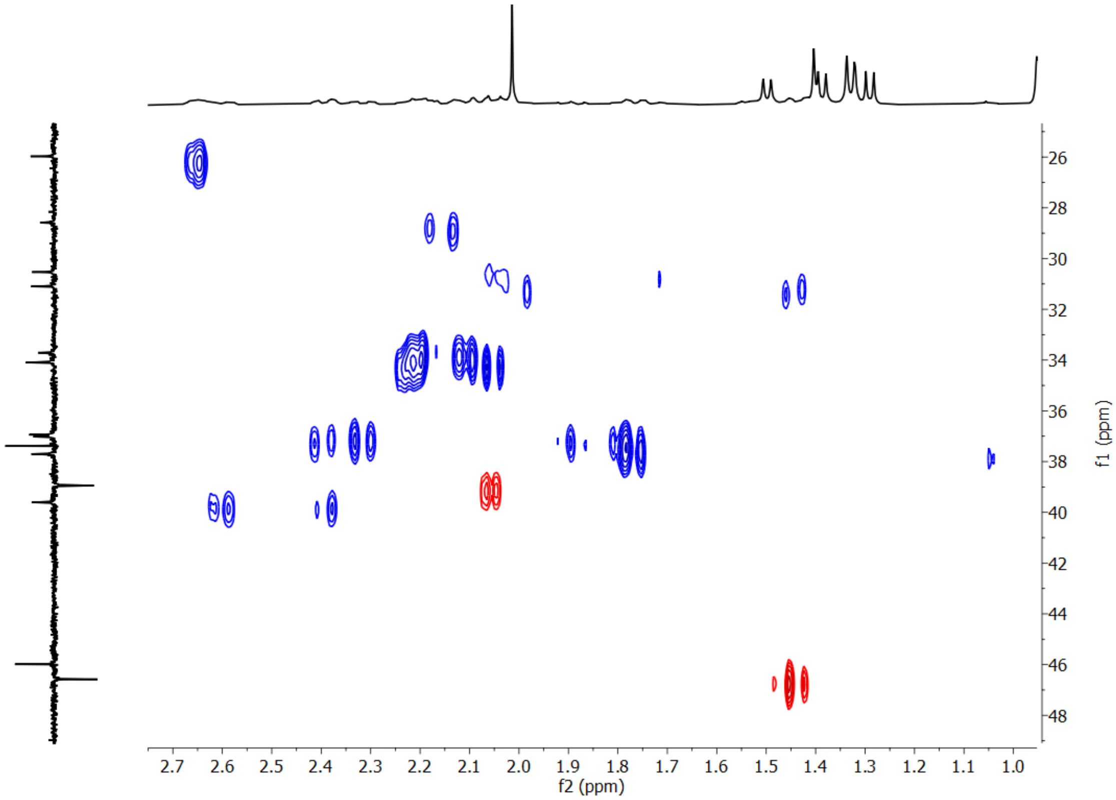

Figure S50: Expansion of HSQC spectrum (400 and 100 MHz, pyridine-d5) of compound *3*

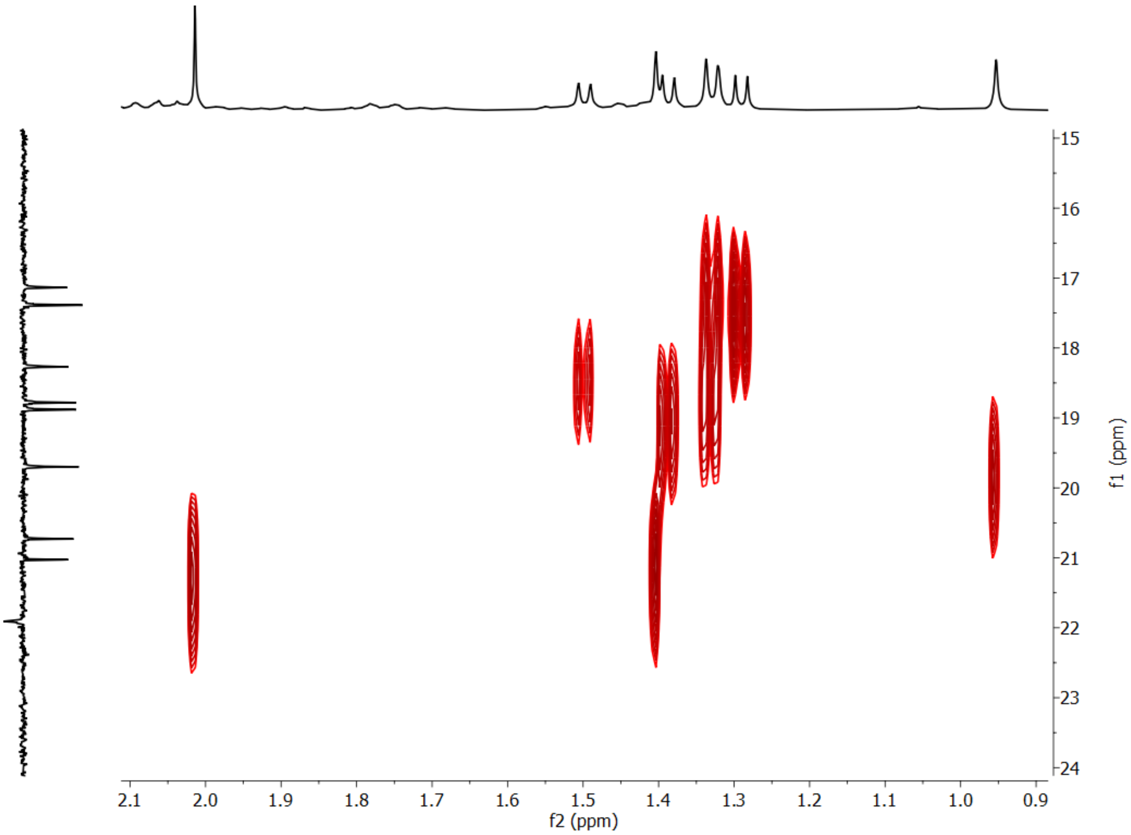

Figure S51: HMBC spectrum(400 and 100 MHz, pyridine-d_5) of compound *3*

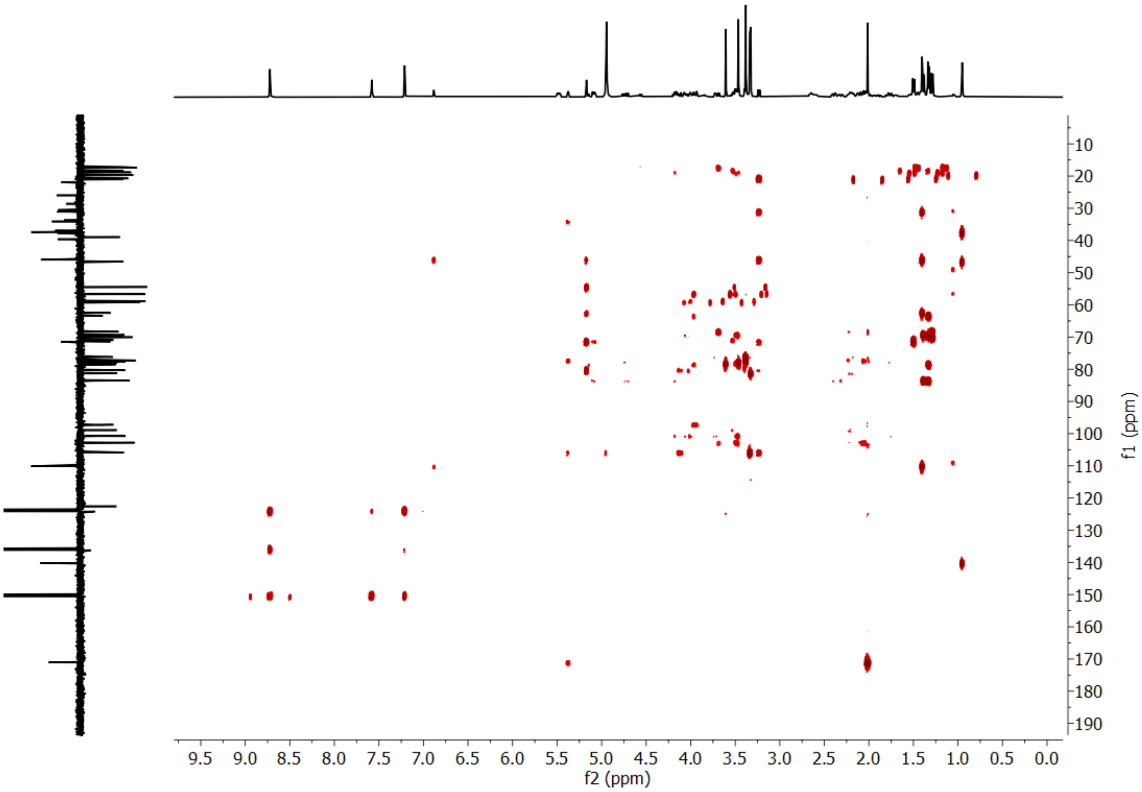

Figure S52: Expansion of HMBC spectrum (400 and 100 MHz, pyridine-d_5) of compound *3*

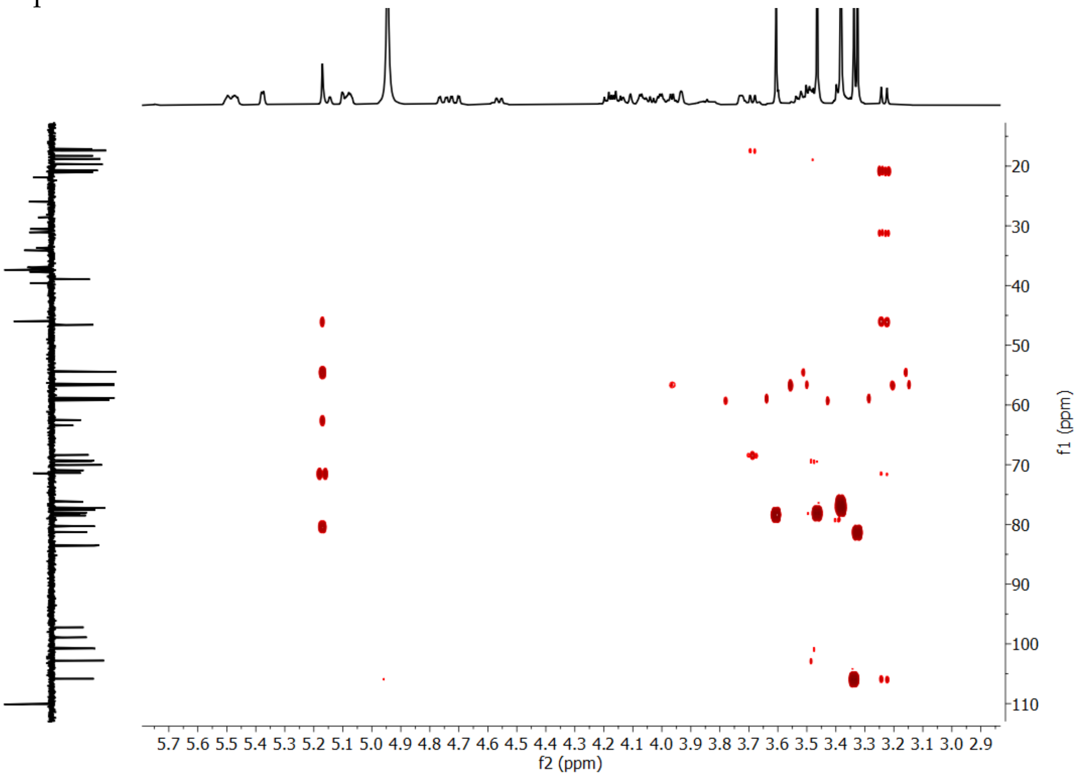

Figure S53: Expansion of HMBC spectrum(400 and 100 MHz, pyridine-d_5) of compound *3*

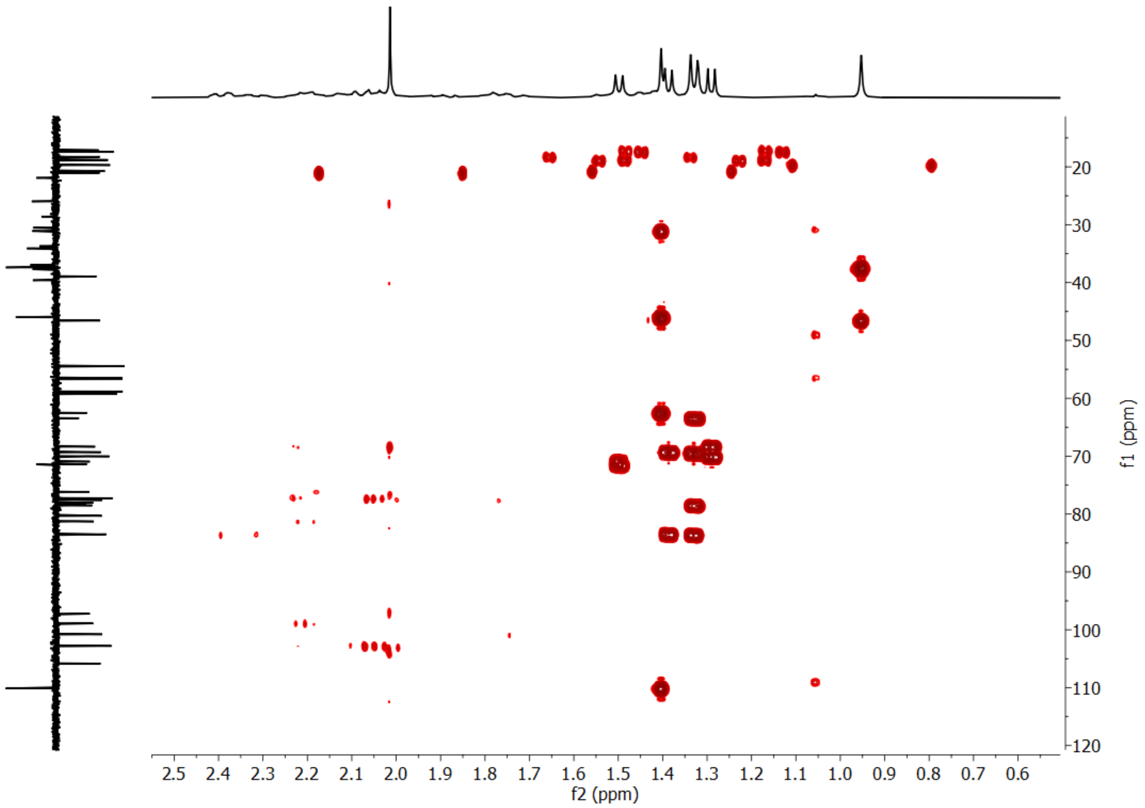

Figure S54: COSY spectrum (400 MHz, pyridine-d_5) of compound **3**

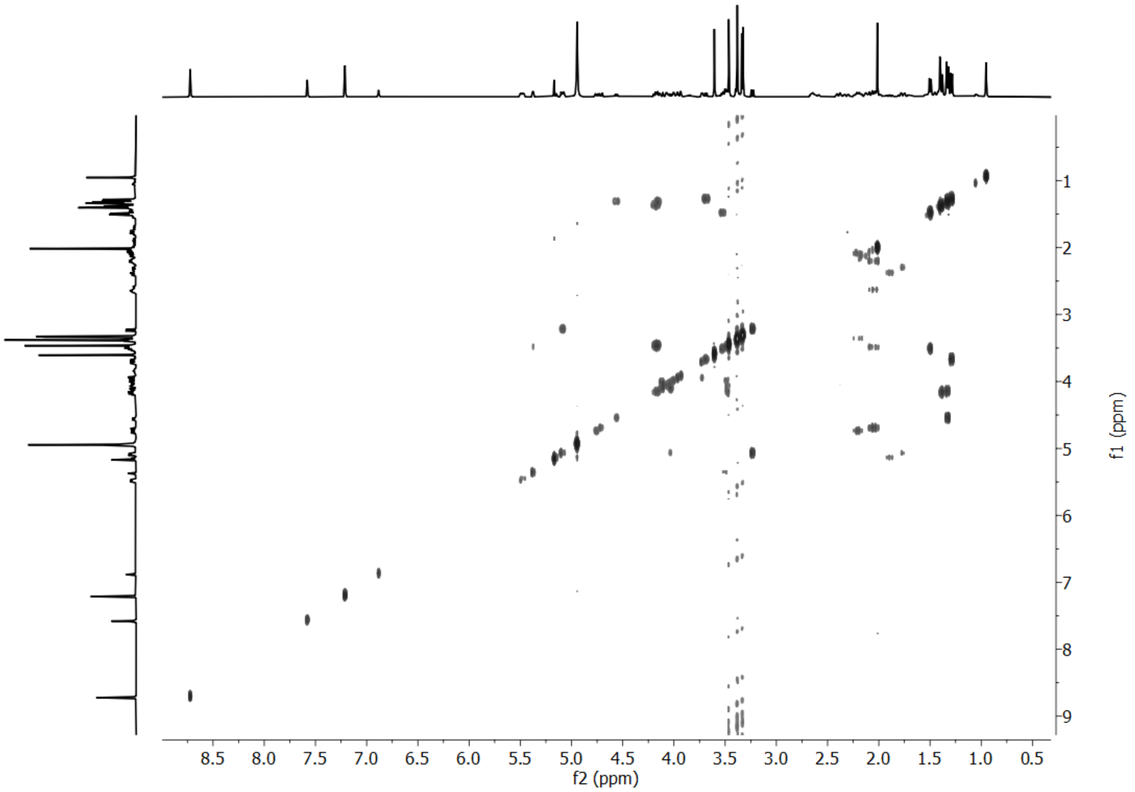

Figure S55: ESI-HRMS spectrum of compound ***4***

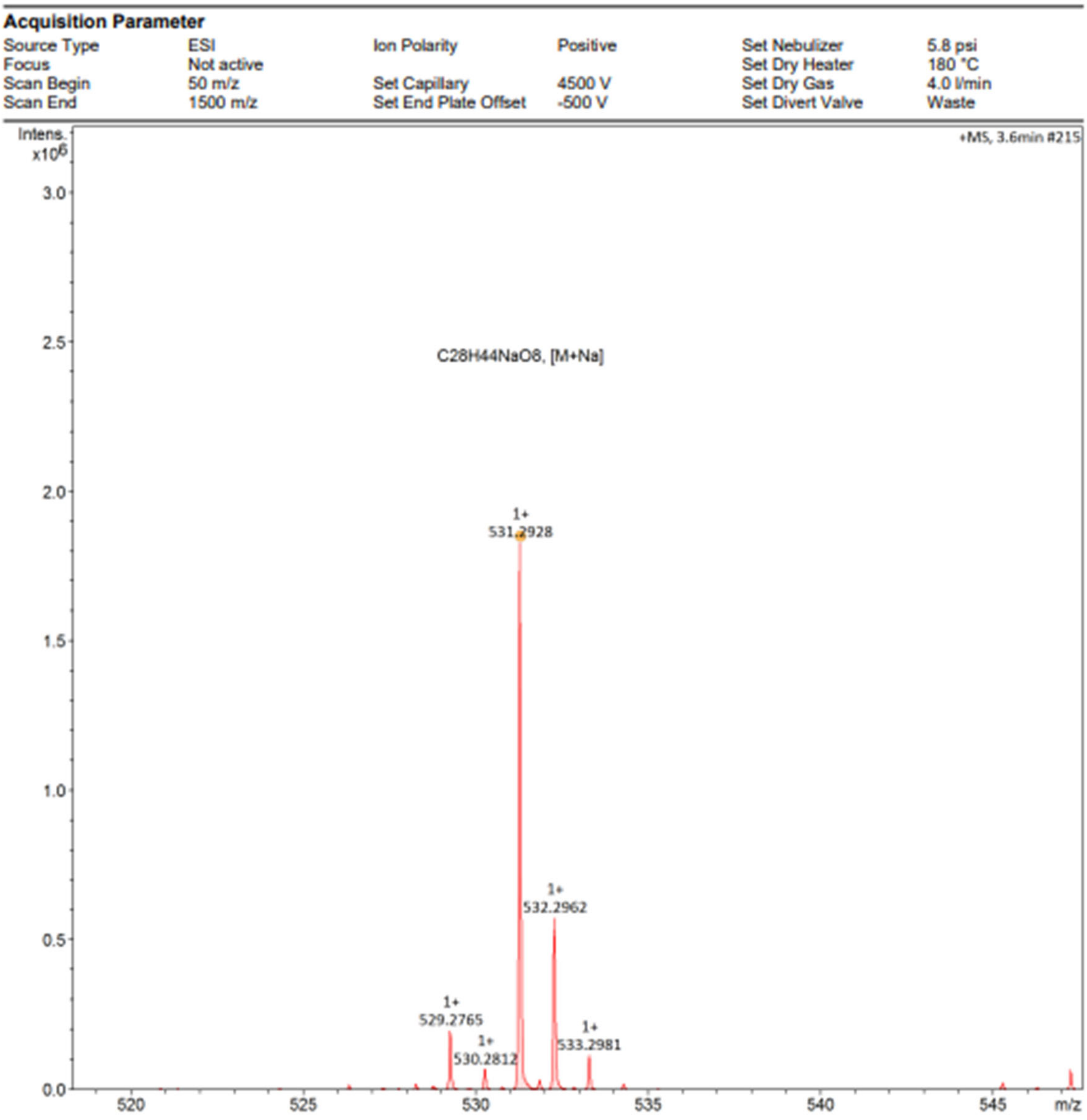

Figure S56: ^1H NMR spectrum (400 MHz, pyridine-d_5) of compound ***4***

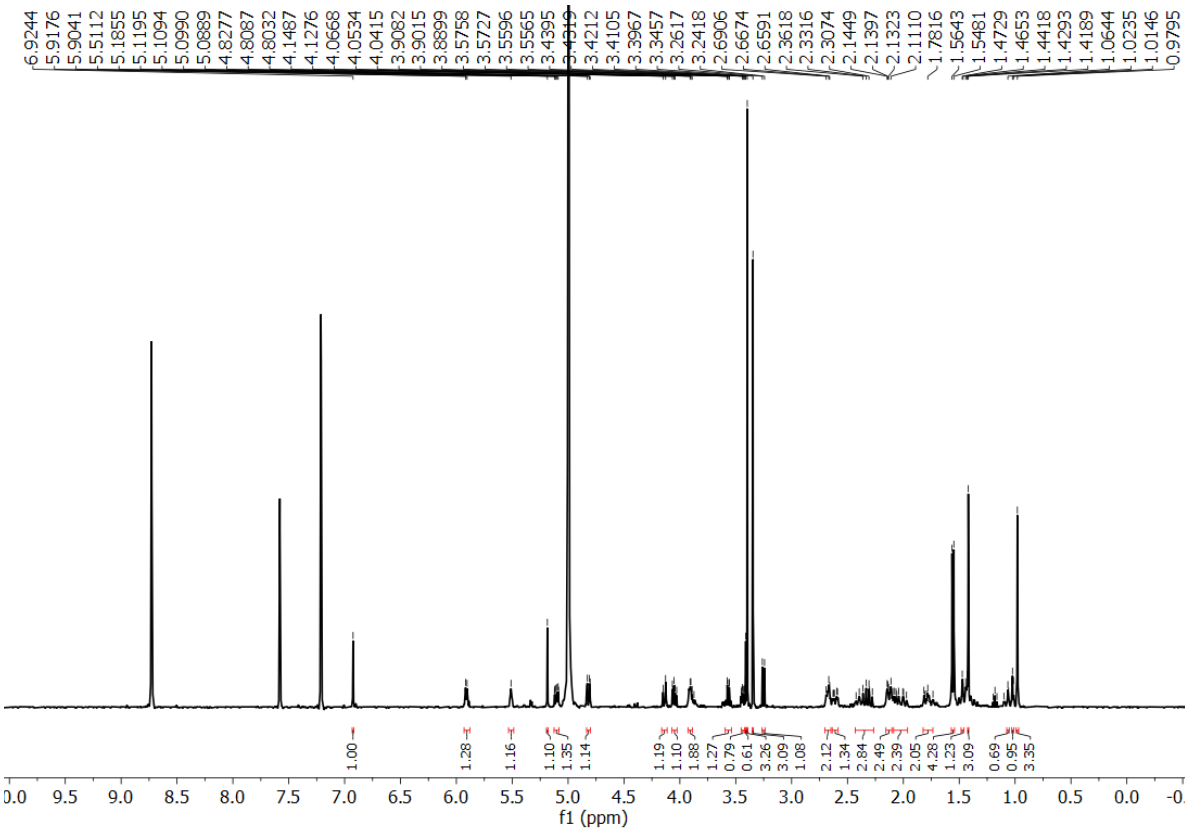

Figure S57: Expansion of ^1H NMR spectrum (400 MHz, pyridine-d_5) of compound ***4***

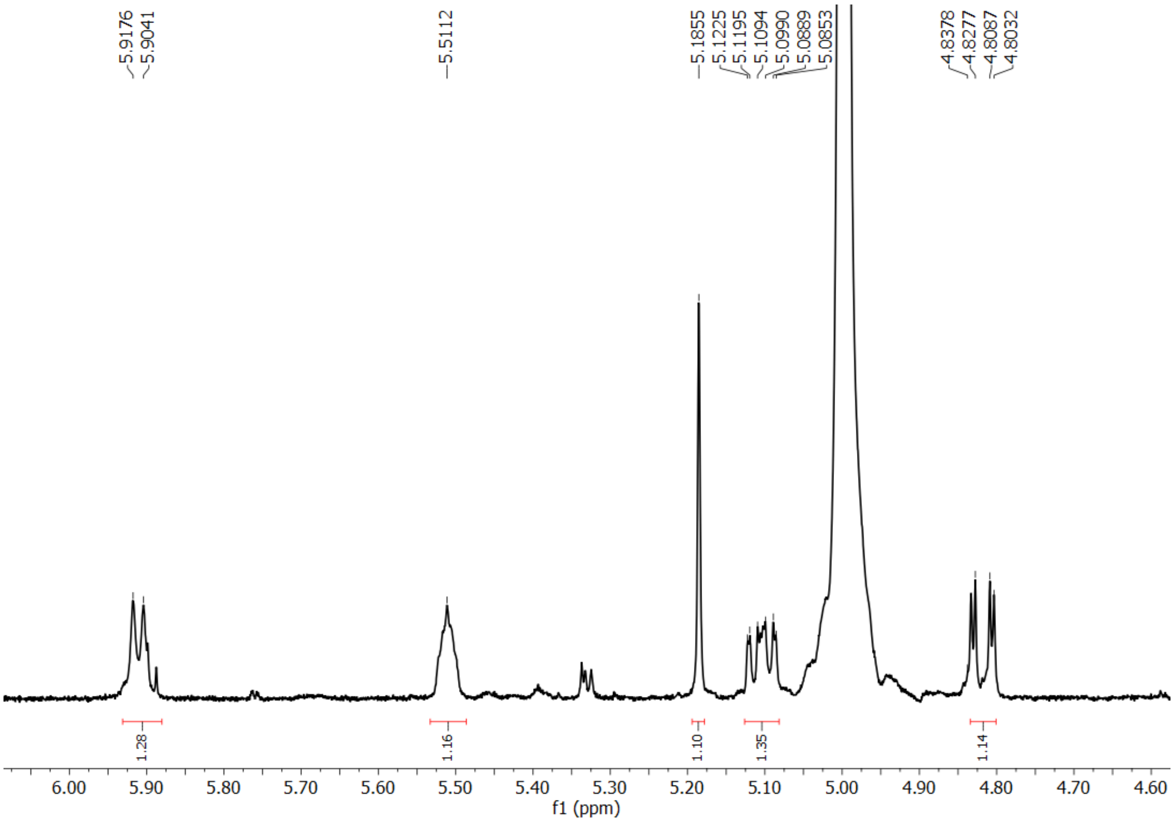

Figure S58: Expansion of ^{1}H NMR spectrum (400 MHz, pyridine-d_5) of compound ***4***

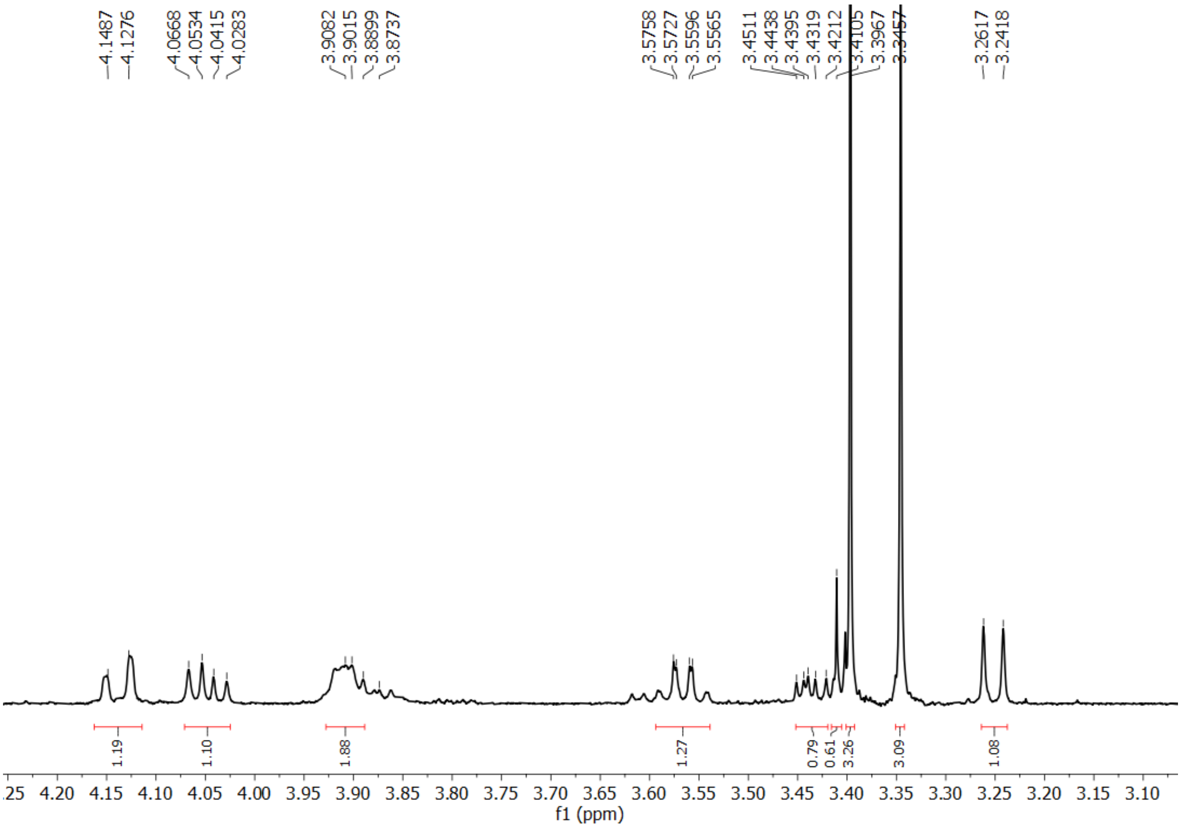

Figure S59: Expansion of ^{1}H NMR spectrum (400 MHz, pyridine-d_5) of compound ***4***

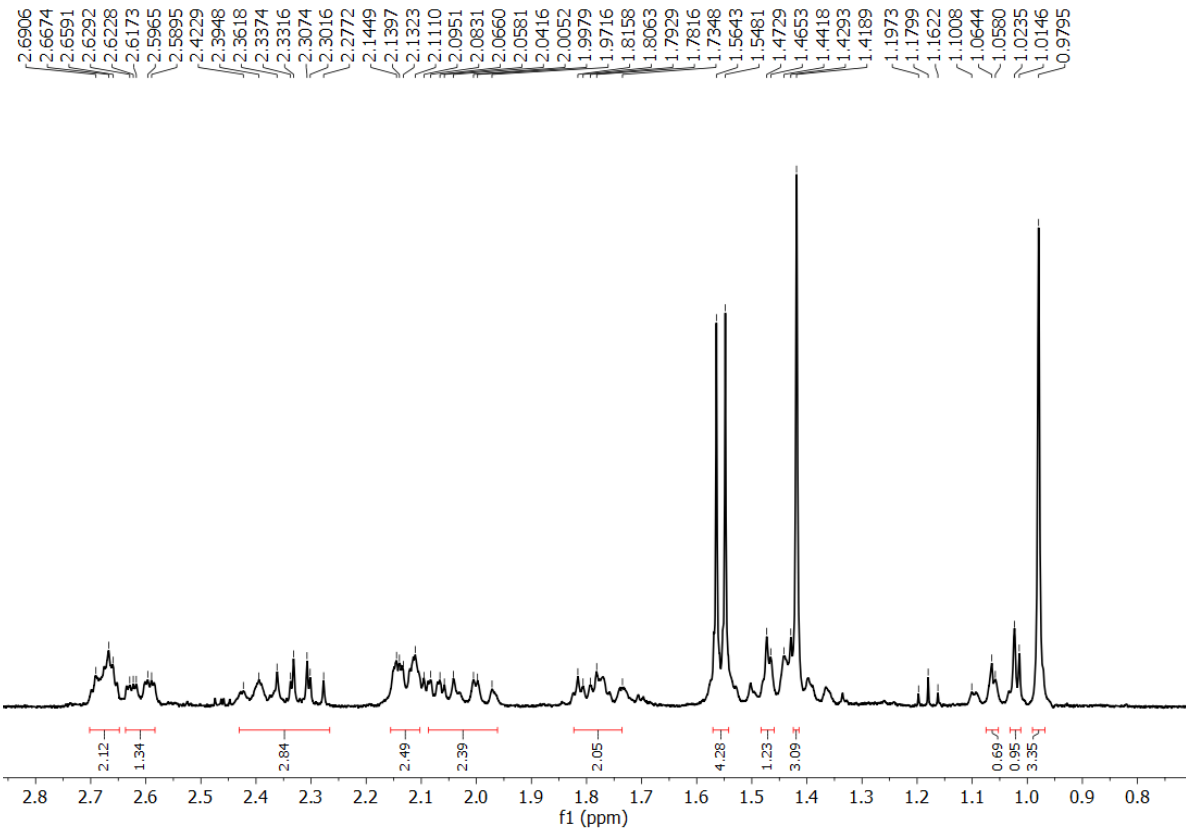

Figure S60: Broadband ^{13}C NMR (100 MHz, pyridine-d_5) of compound ***4***

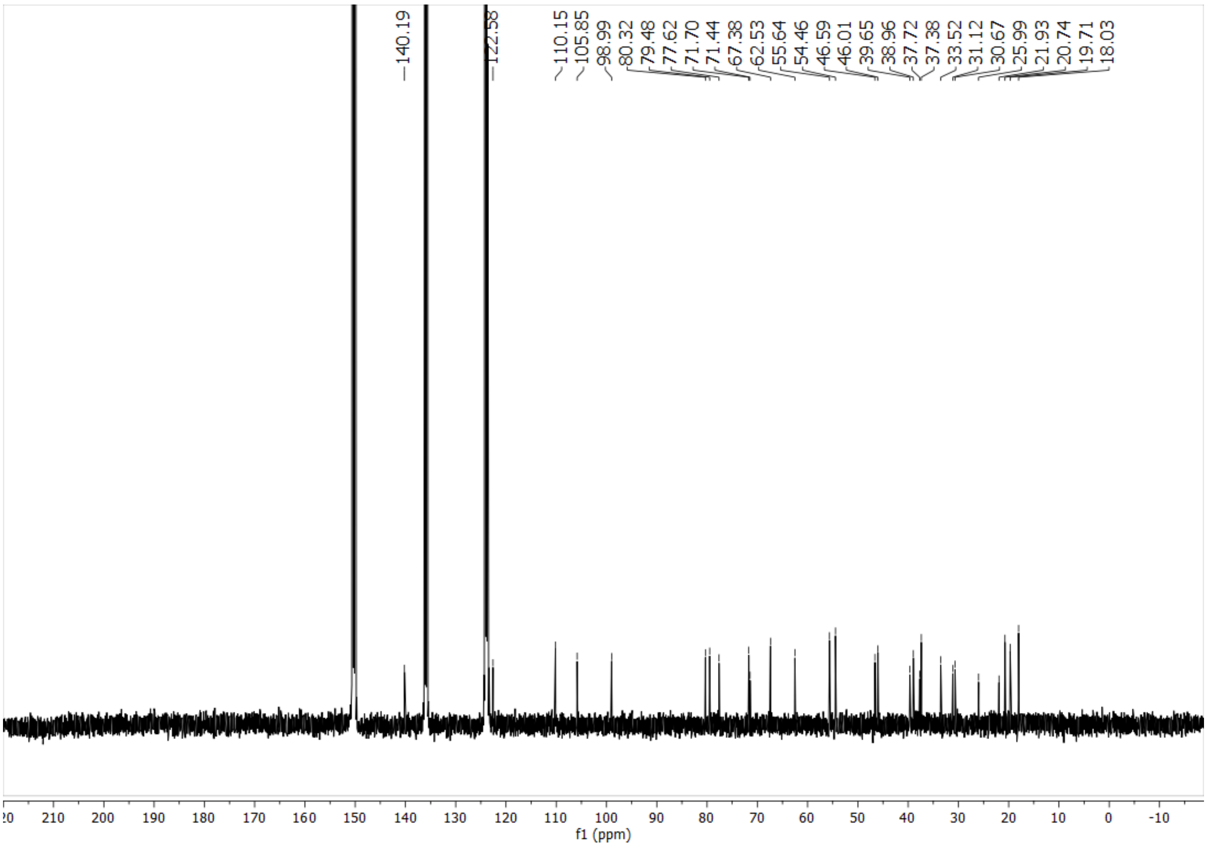

Figure S61: DEPT 135 spectrum (100 MHz, pyridine-d_5) of compound ***4***

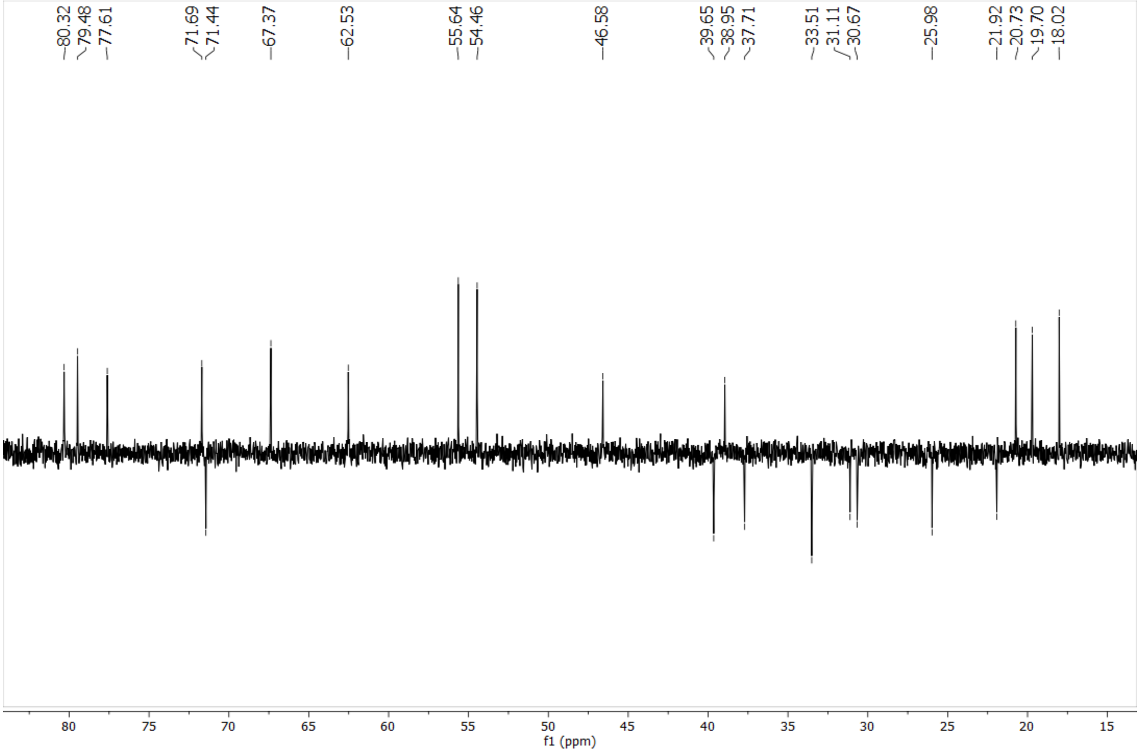

Figure S62: Expansion of DEPT 135 spectrum (100 MHz, pyridine-d5) of compound ***4***

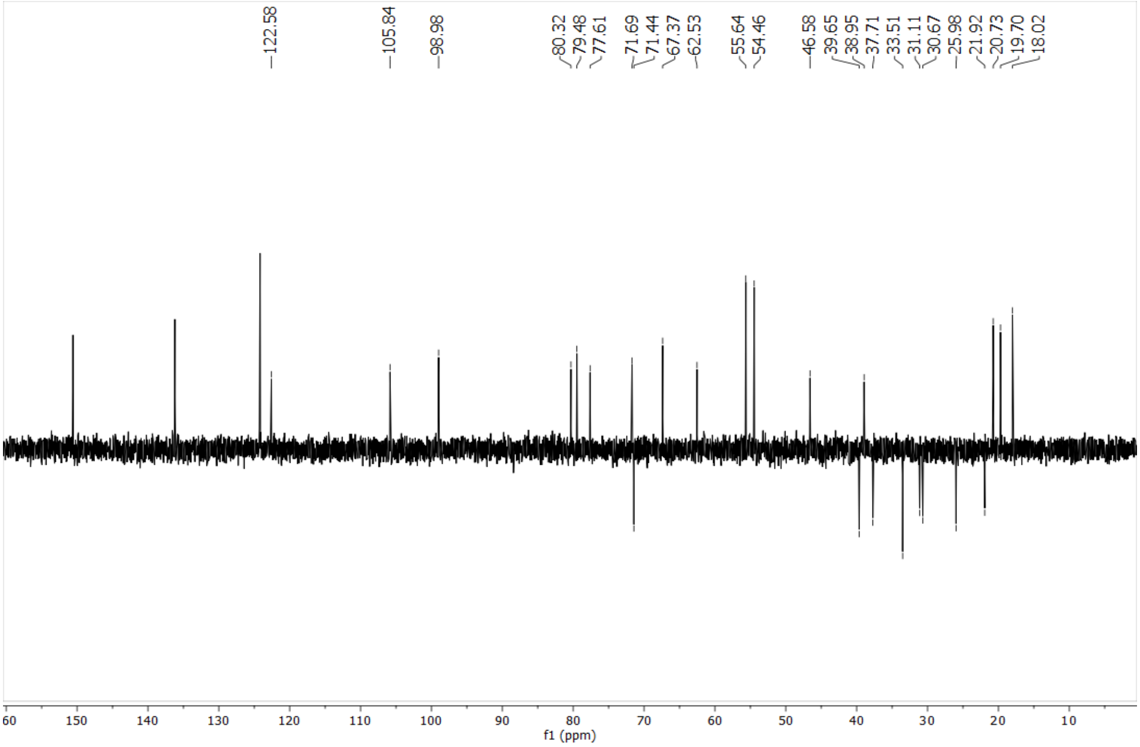

Figure S63: HSQC spectrum (400 and 100 MHz, pyridine-d5)of compound ***4***

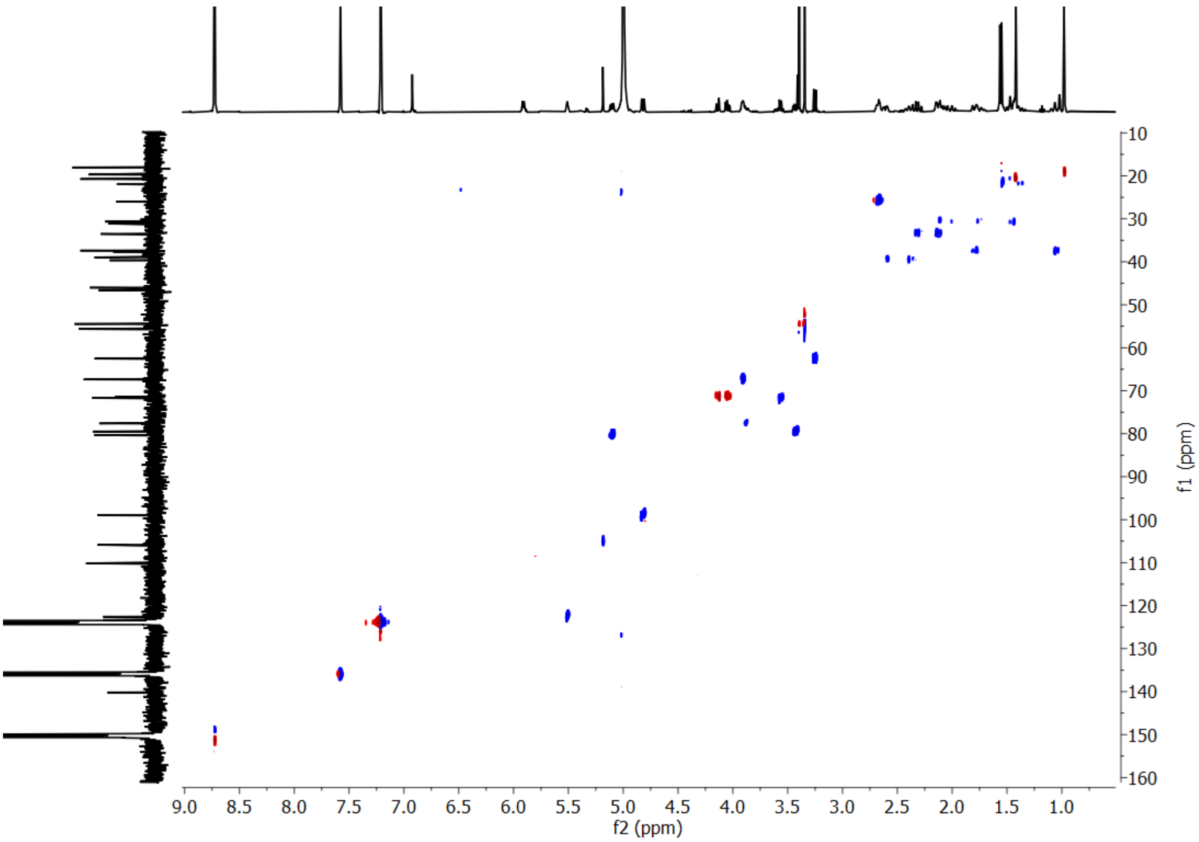

Figure S64: Expansion of HSQC spectrum (400 and 100 MHz, pyridine-d5)of compound *4*

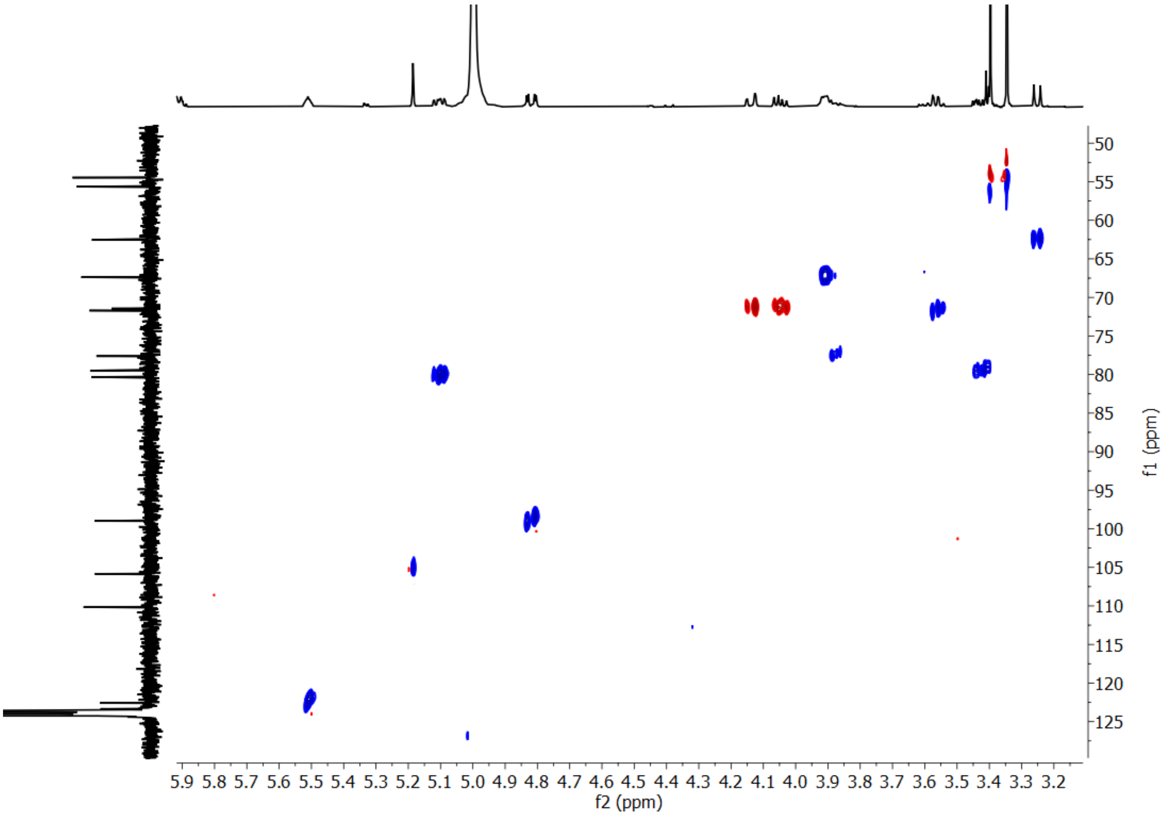

Figure S65: Expansion of HSQC spectrum (400 and 100 MHz, pyridine-d5) of compound *4*

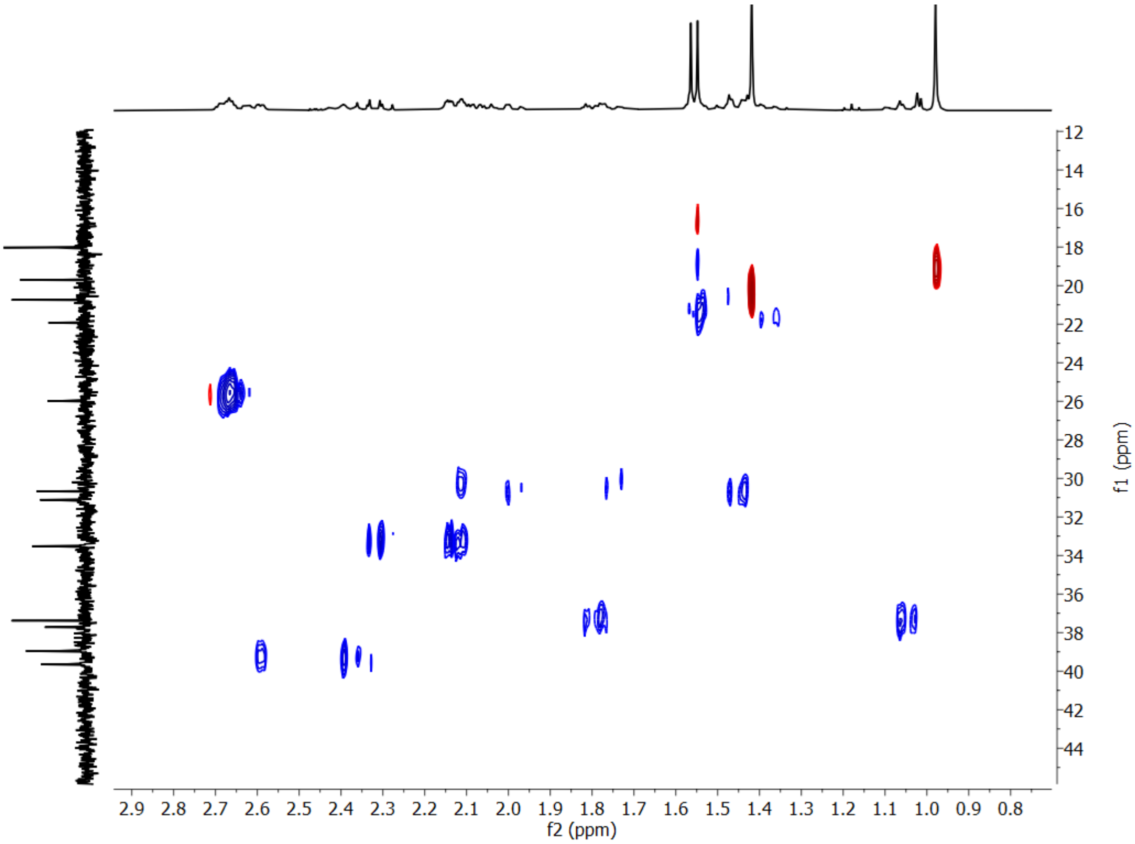

Figure S66: HMBC spectrum (400 and 100 MHz, pyridine-d5) of compound ***4***

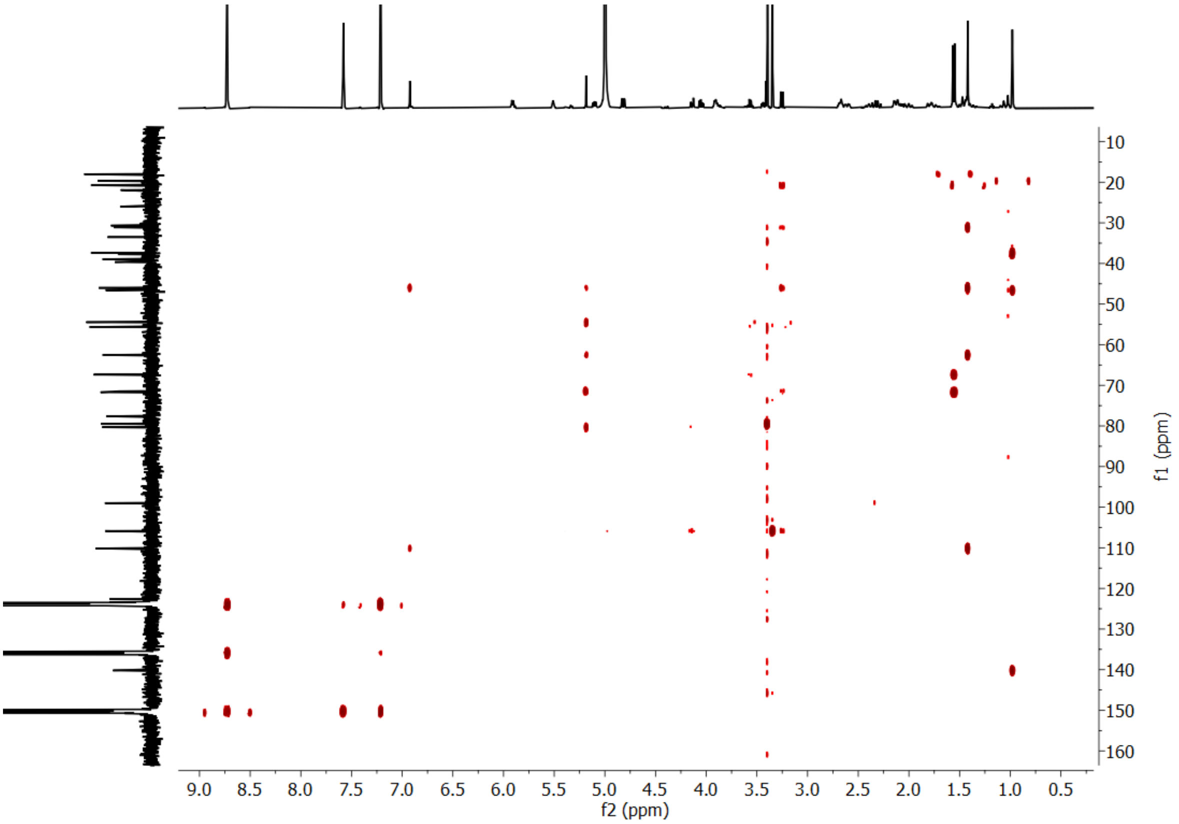

Figure S67: Expansion of HMBC spectrum (400 and 100 MHz, pyridine-d5) of compound ***4***

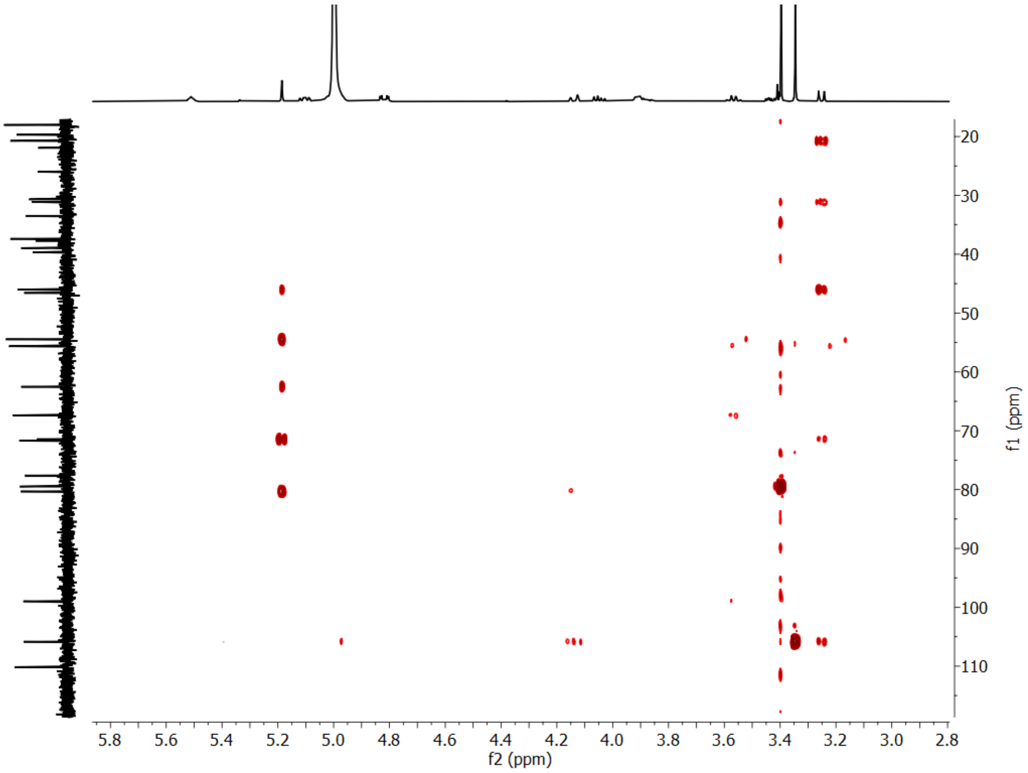

Figure S68: HMBC spectrum (400 and 100 MHz, pyridine-d5) of compound ***4***

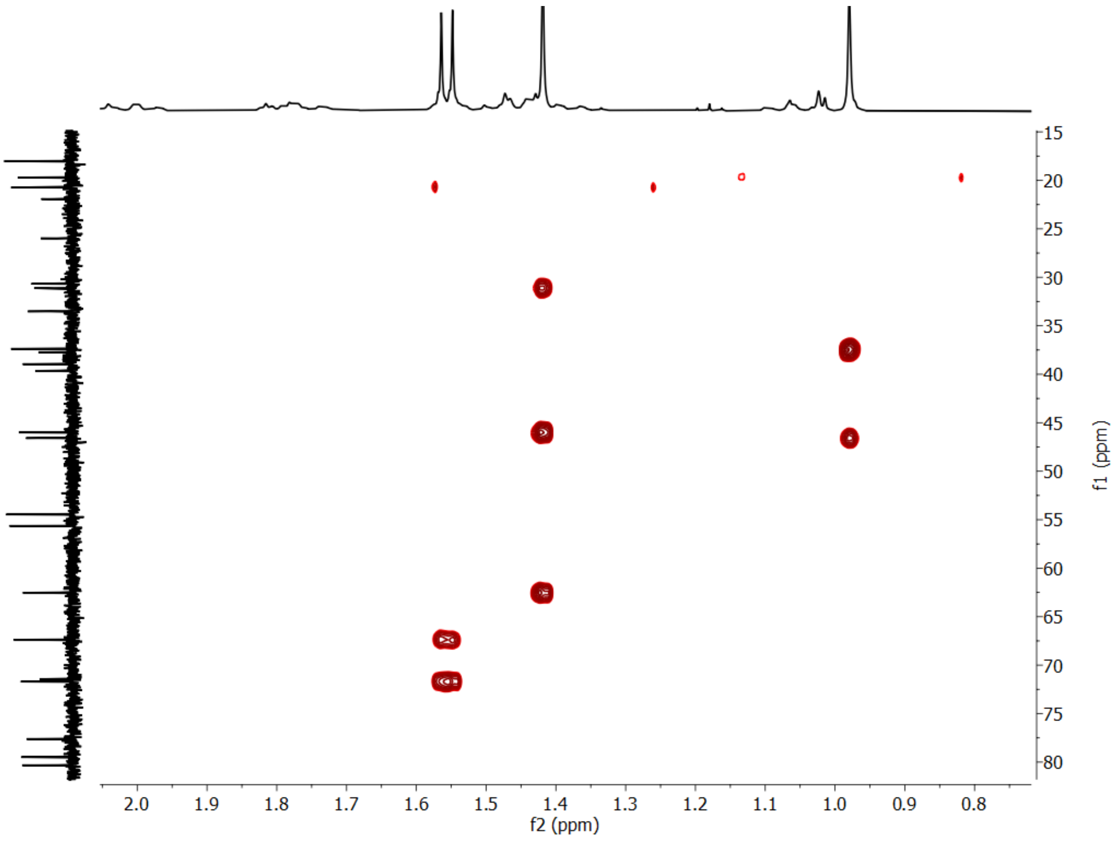

Figure S69: COSY spectrum (400 MHz, pyridine-d5) of compound ***4***

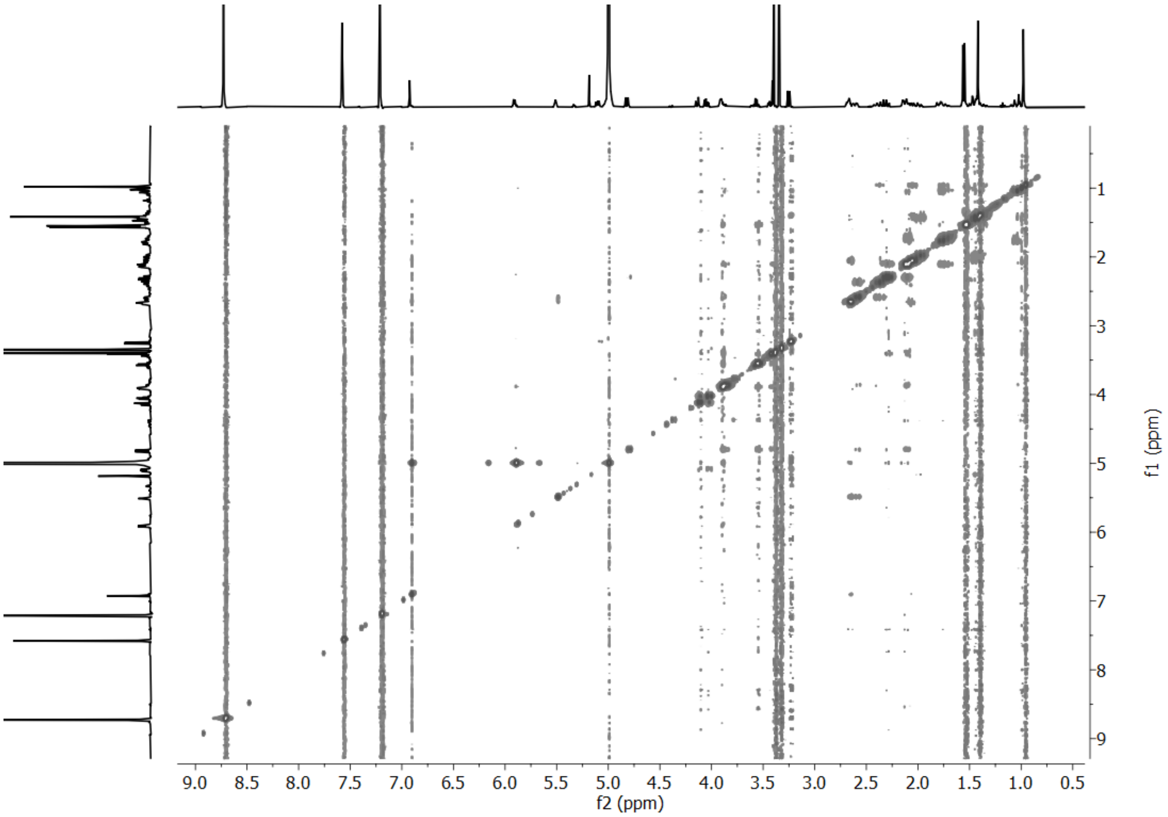

Figure S70: ESI-HRMS spectrum of compound *5*

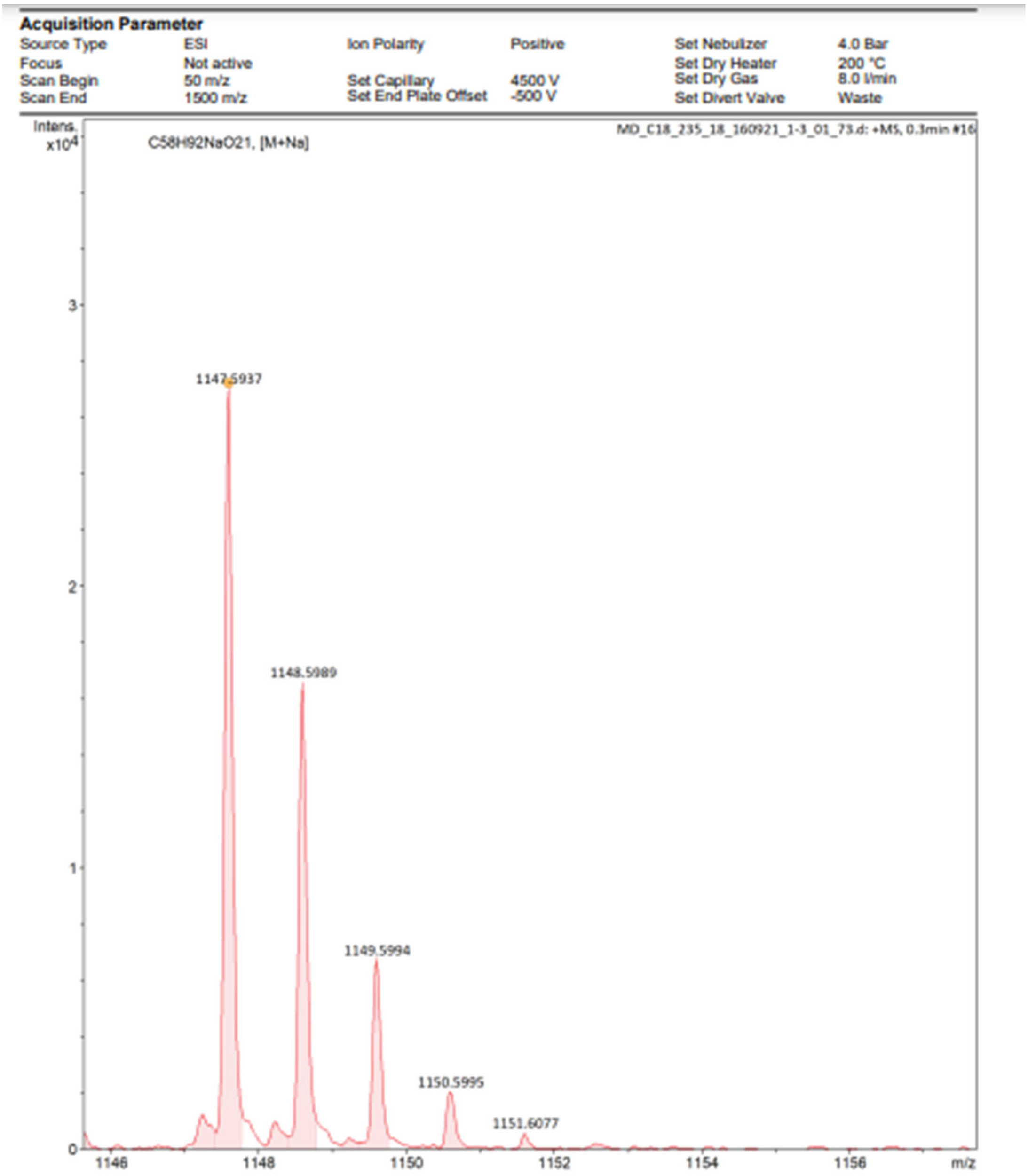

Figure S71: ^{1}H NMR spectrum (400 MHz, pyridine-d_5) of compound ***5***

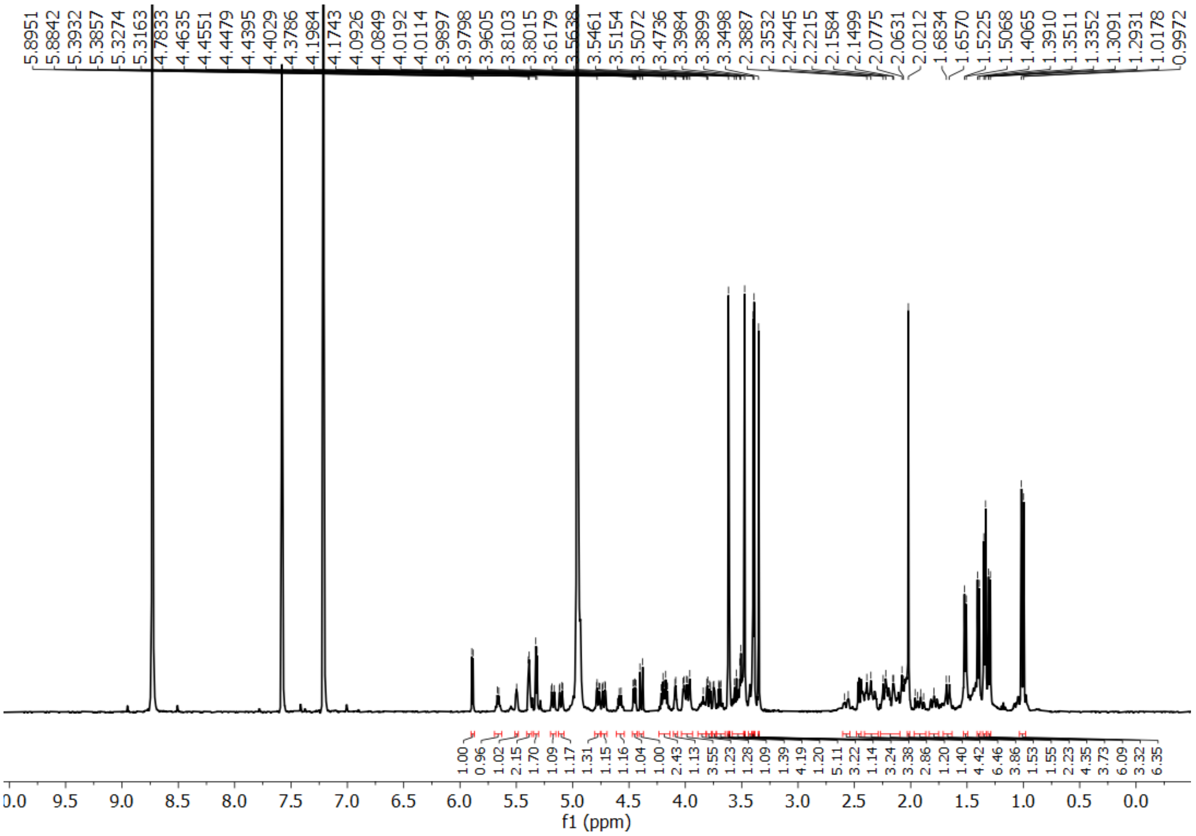

Figure S72: Expansion of ^{1}H NMR spectrum (400 MHz, pyridine-d_5) of compound ***5***

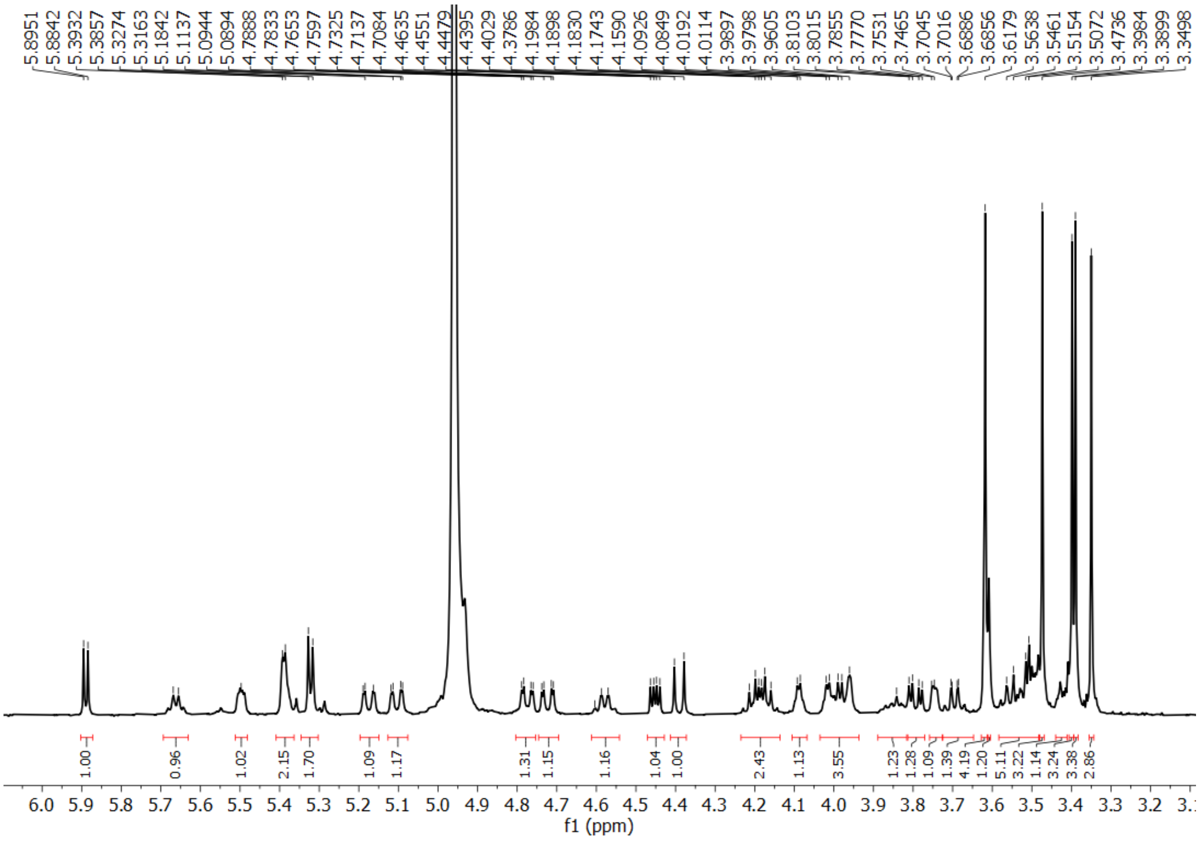

Figure S73: Expansion of ^{1}H NMR spectrum (400 MHz, pyridine-d_5) of compound ***5***

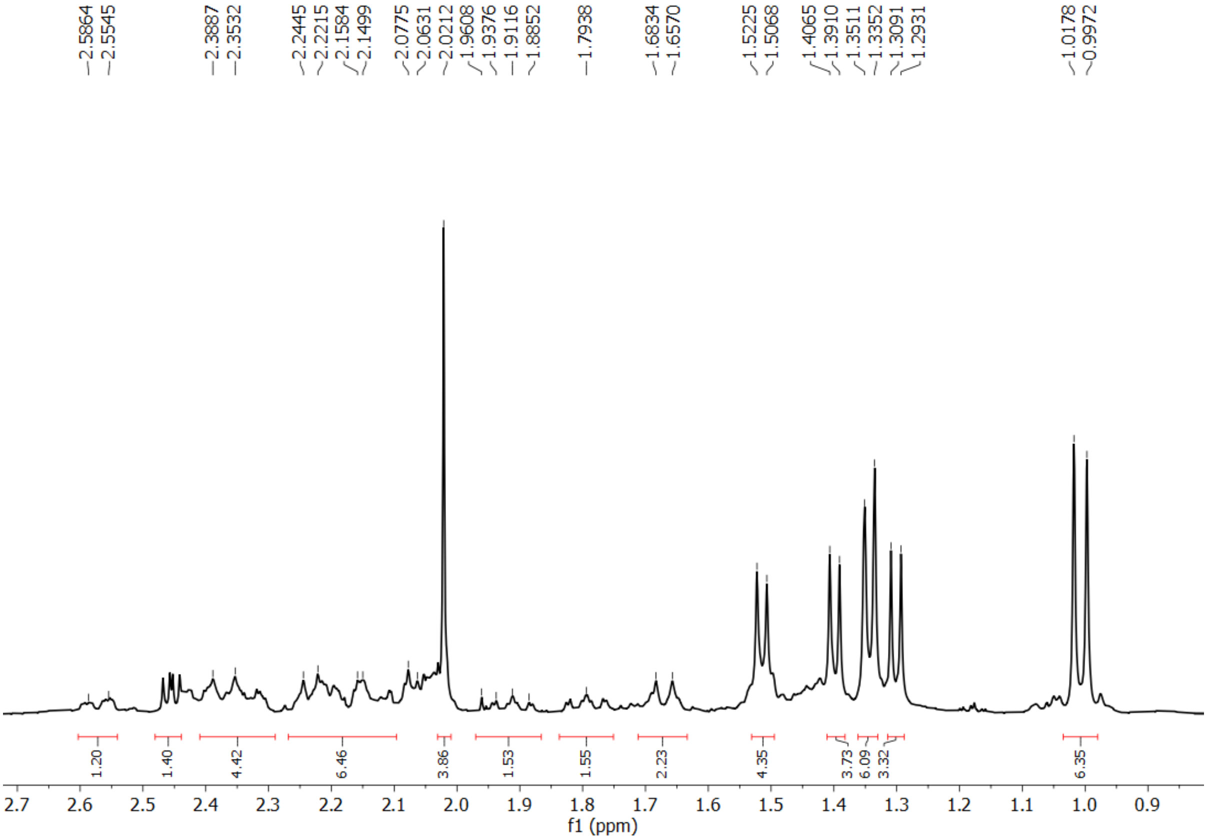

Figure S74: ^{13}C NMR spectrum (100 MHz, pyridine-d_5) of compound ***5***

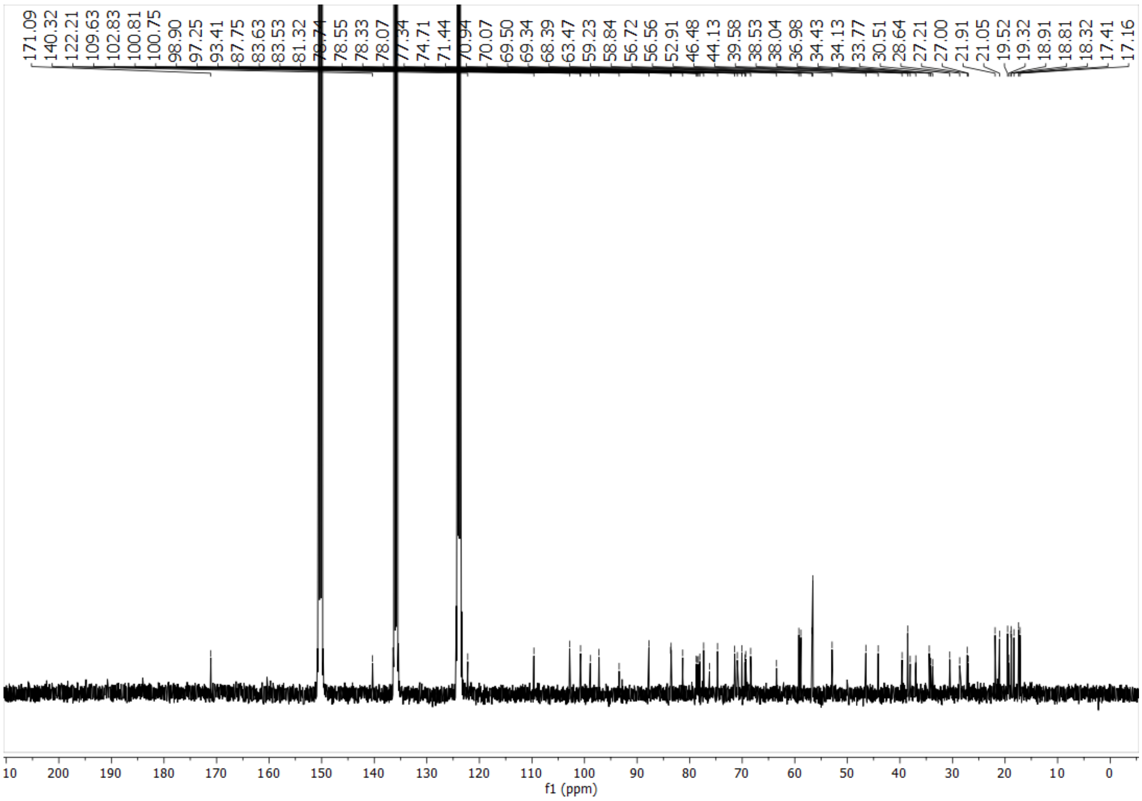

Figure S75: Expansion of ^{13}C NMR spectrum (100 MHz, pyridine-d_5) of compound ***5***

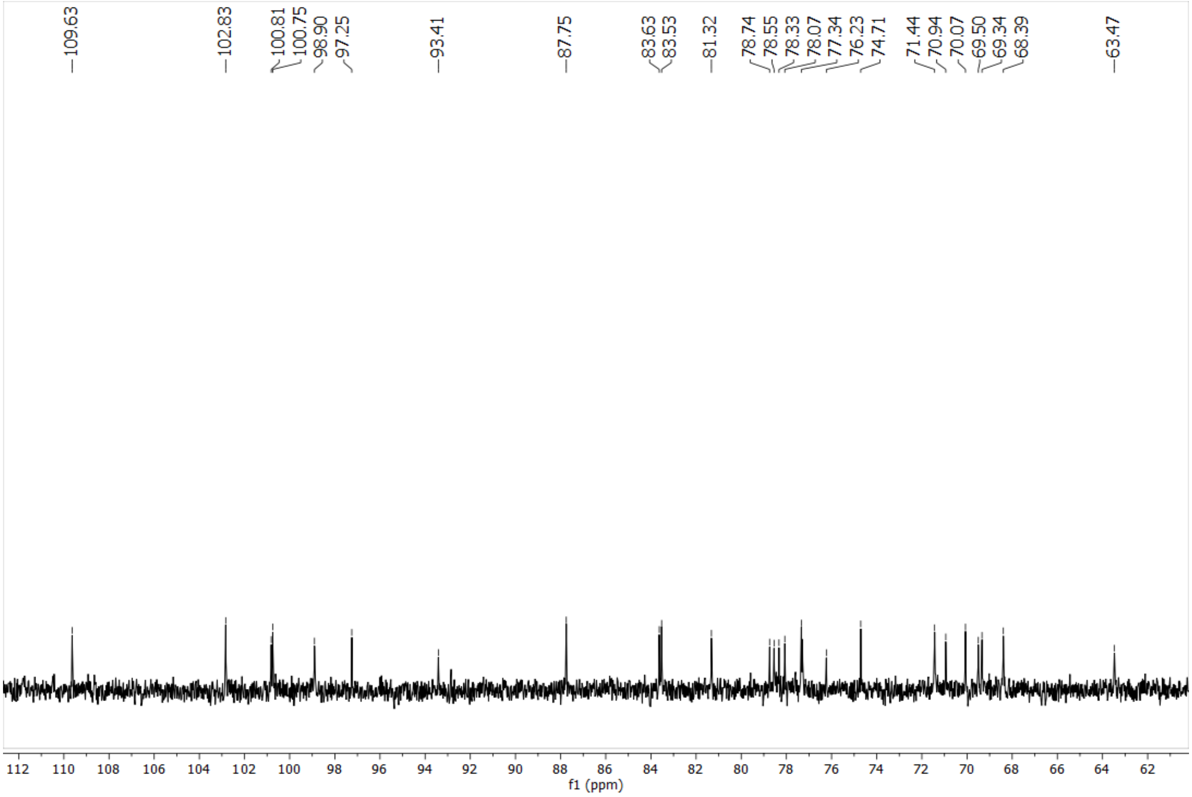

Figure S76: Expansion of ^{13}C NMR spectrum (100 MHz, pyridine-d_5) of compound ***5***

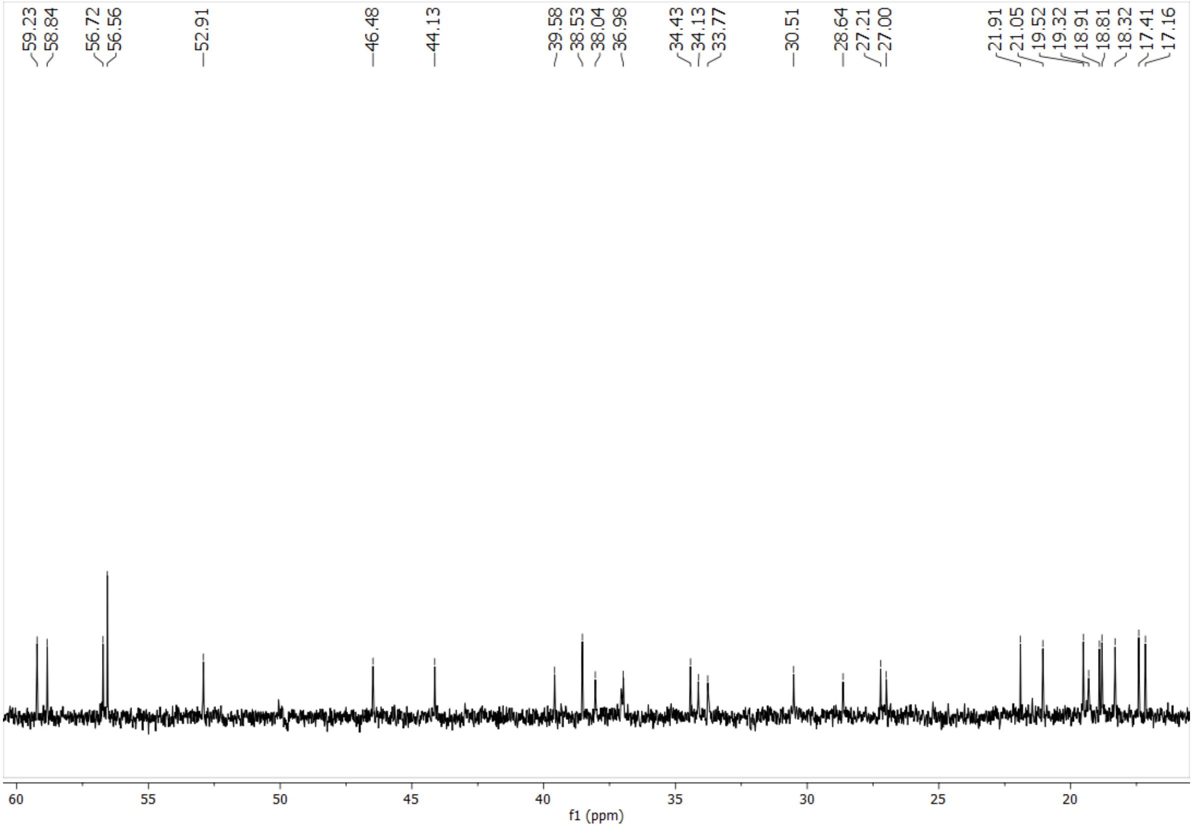

Figure S77: DEPT spectrum (100 MHz, pyridine-d5) of compound ***5***

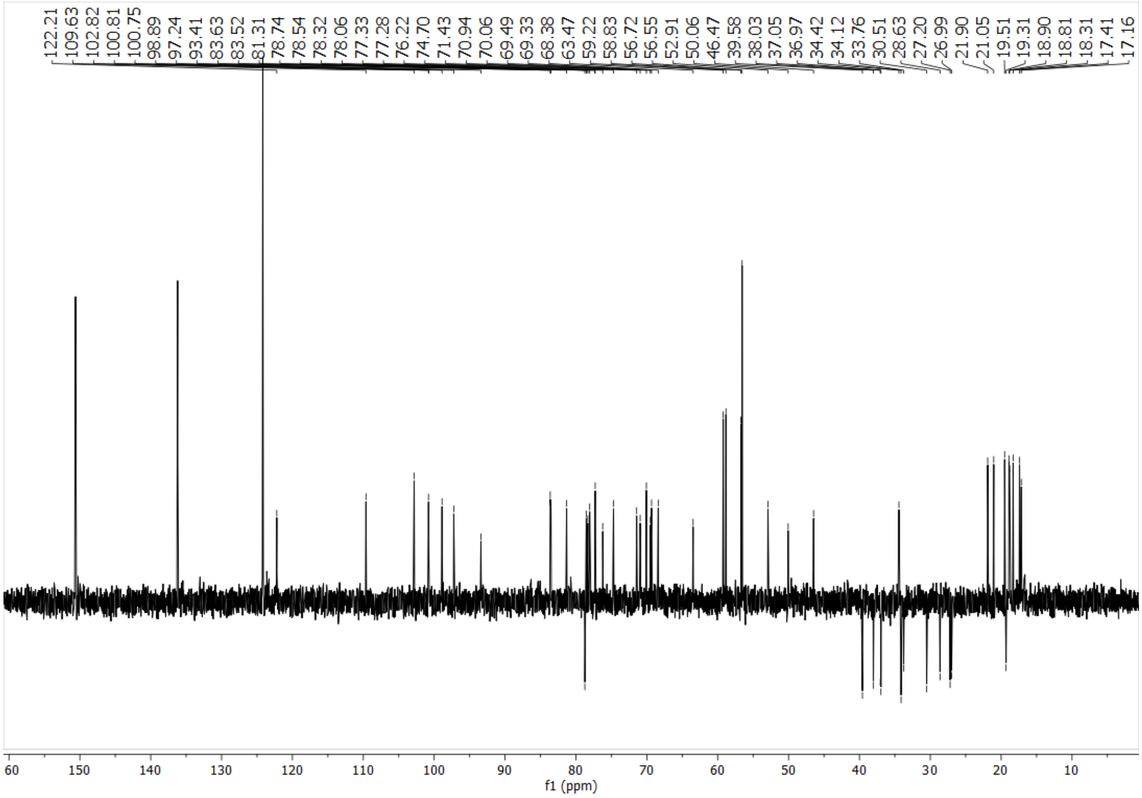

Figure S78: Expansion of DEPT spectrum (100 MHz, pyridine-d5) of compound ***5***

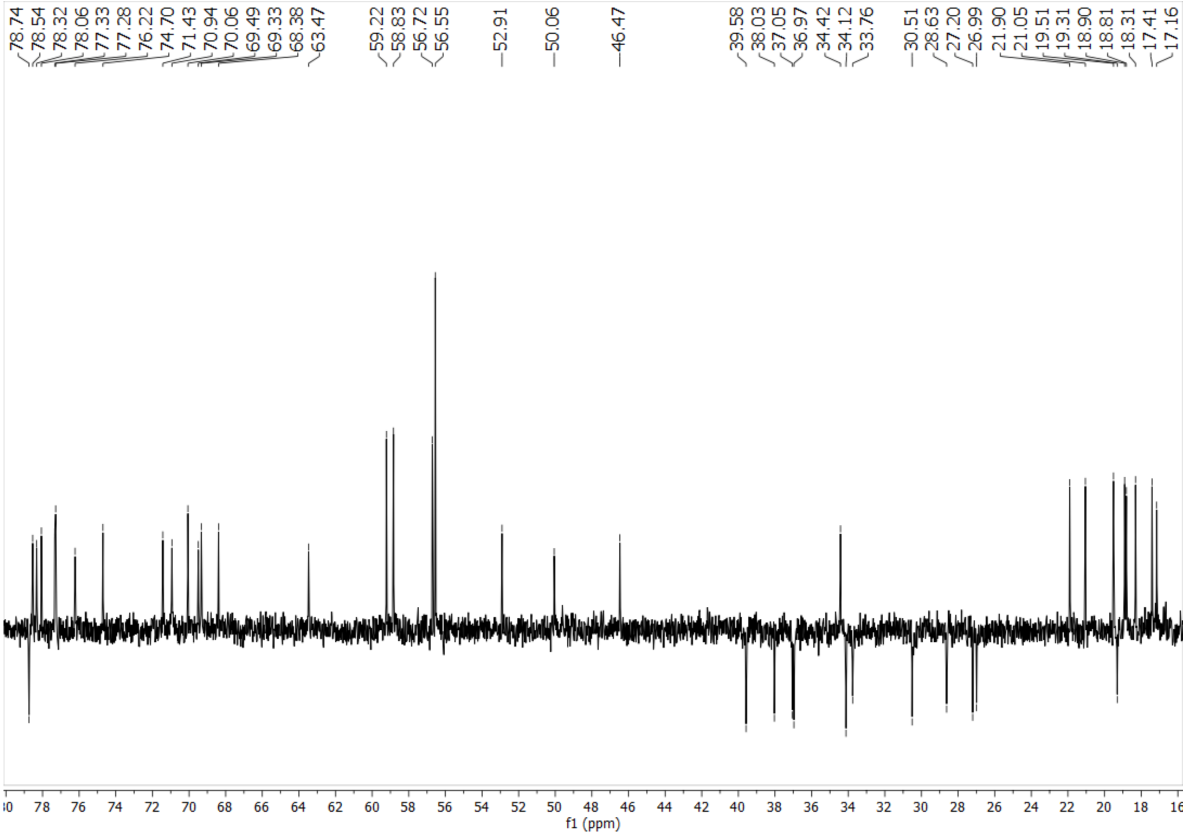

Figure S79: HSQC spectrum (400 and 100 MHz, pyridine-d5) of compound ***5***

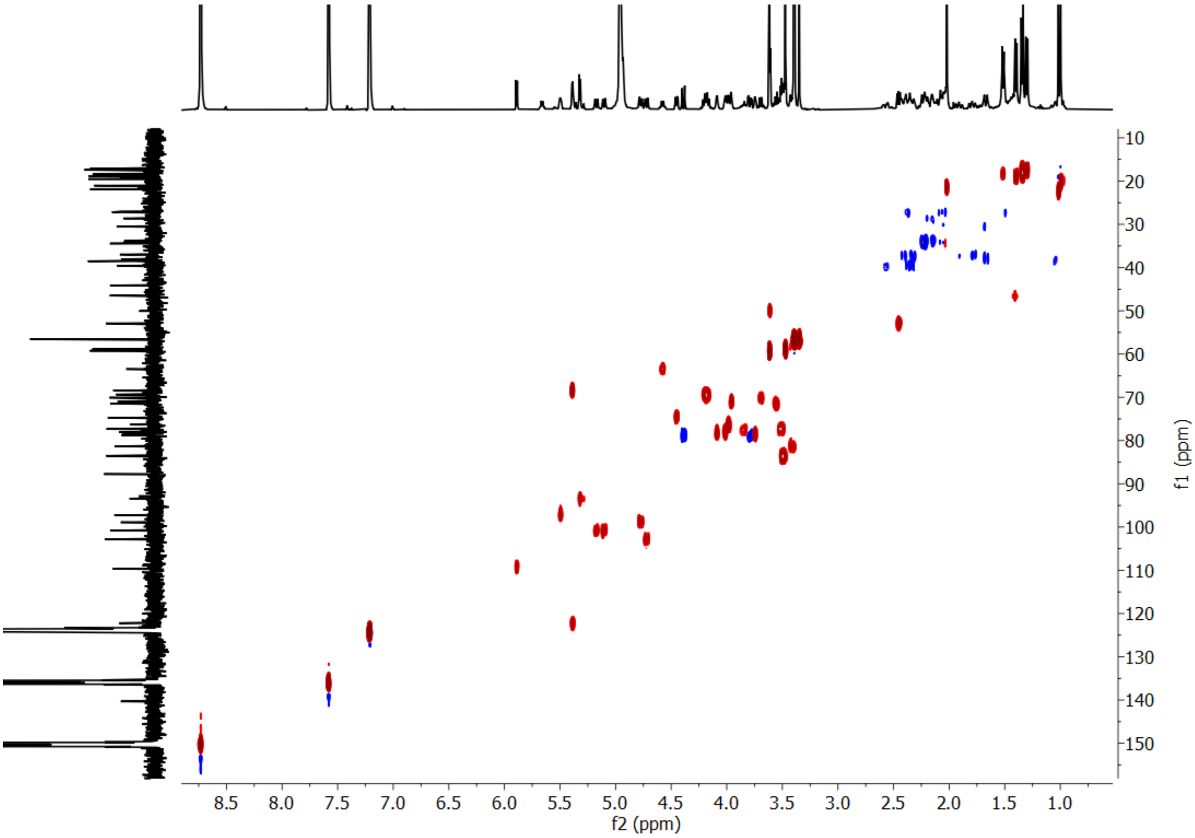

Figure S80: HSQC spectrum (400 and 100 MHz, pyridine-d5) of compound ***5***

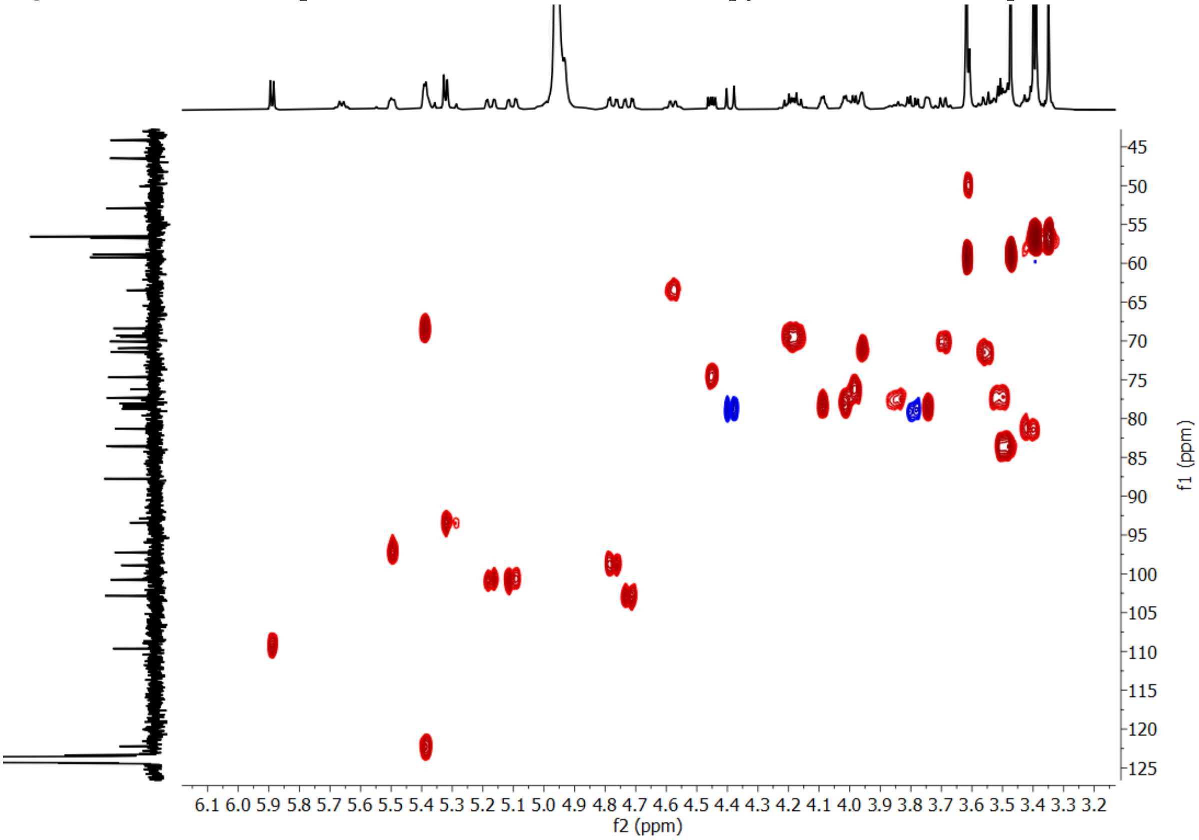

Figure S81: HSQC spectrum (400 and 100 MHz, pyridine-d5) of compound ***5***

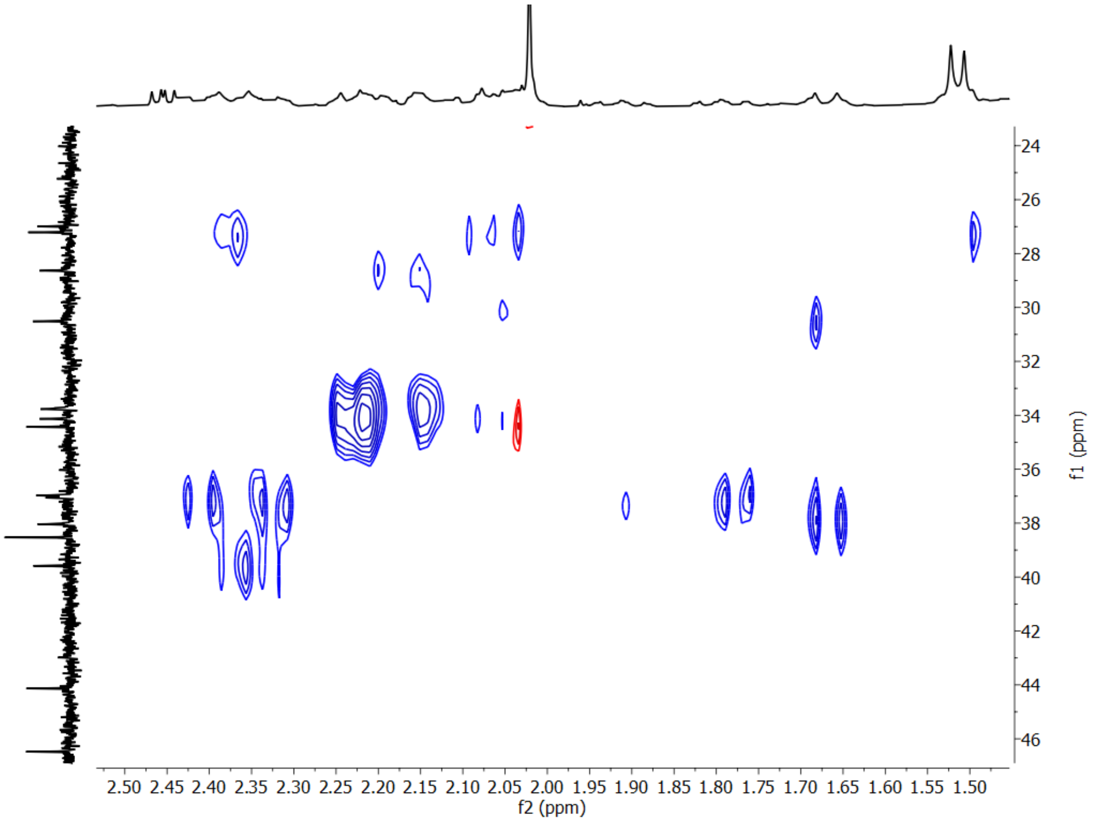

Figure S82: HMBC spectrum (400 and 100 MHz, pyridine-d5) of compound ***5*** in pyridine-d5, 400 MHz

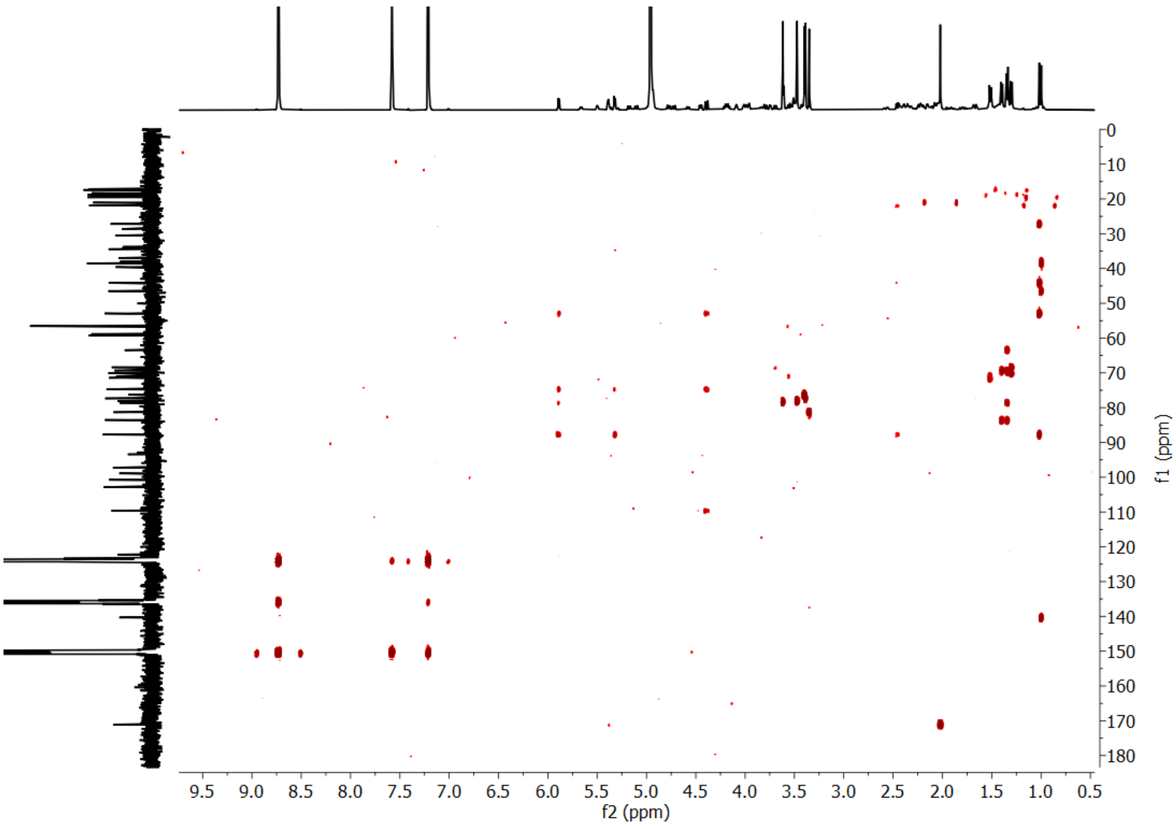

Figure S83: HMBC spectrum (400 and 100 MHz, pyridine-d5) of compound **5**

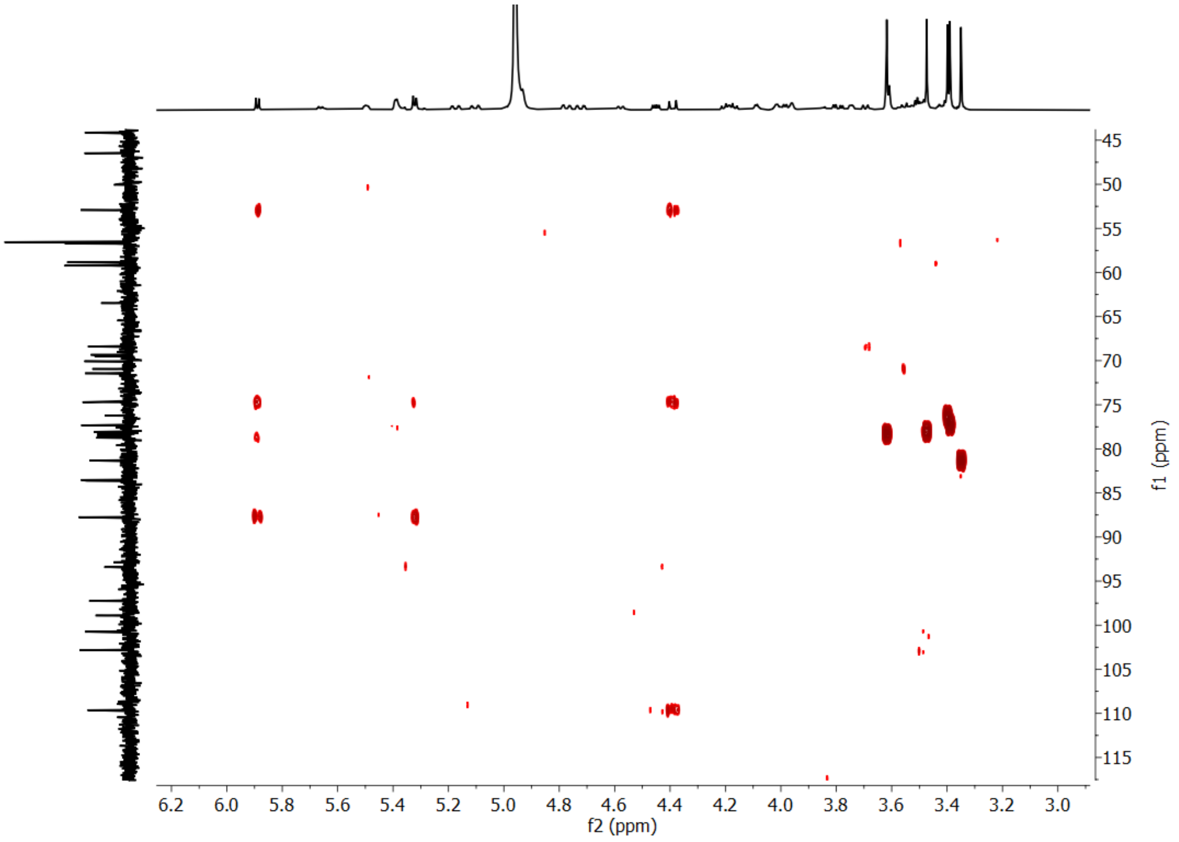

Figure S84: HMBC spectrum (400 and 100 MHz, pyridine-d5) of compound **5**

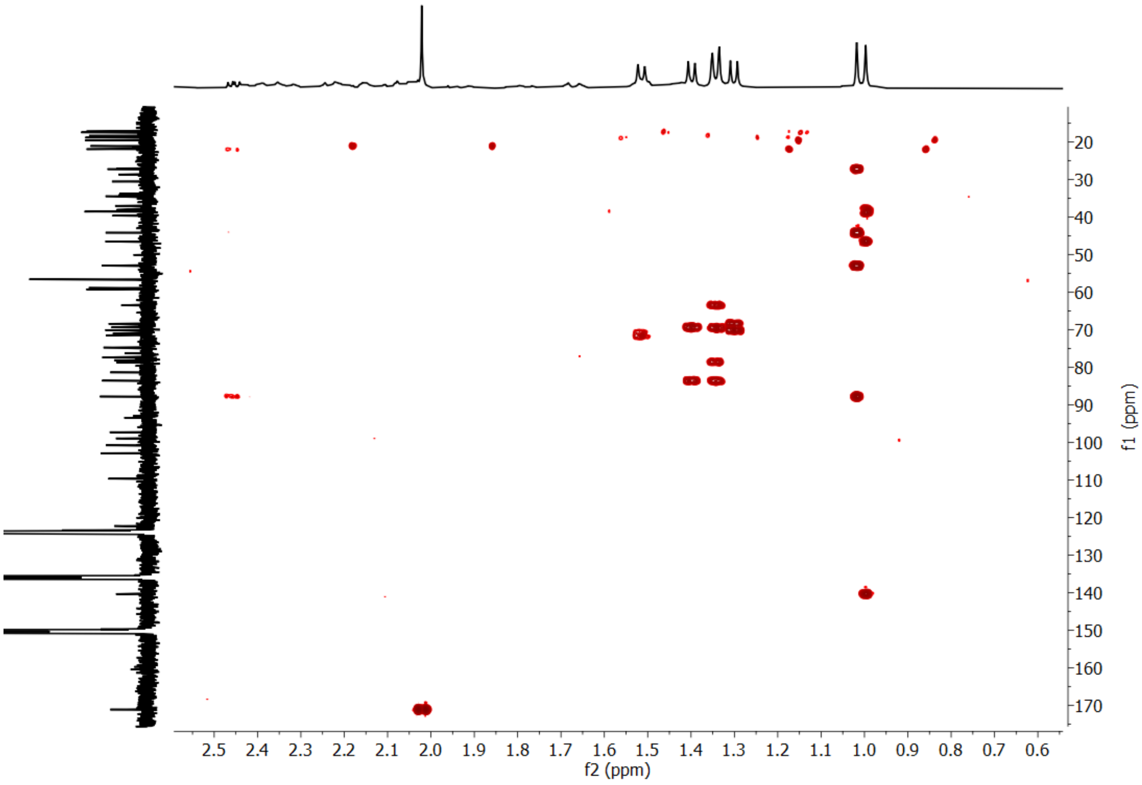

Figure S85: COSY spectrum (400 MHz, pyridine-d5) of compound ***5***

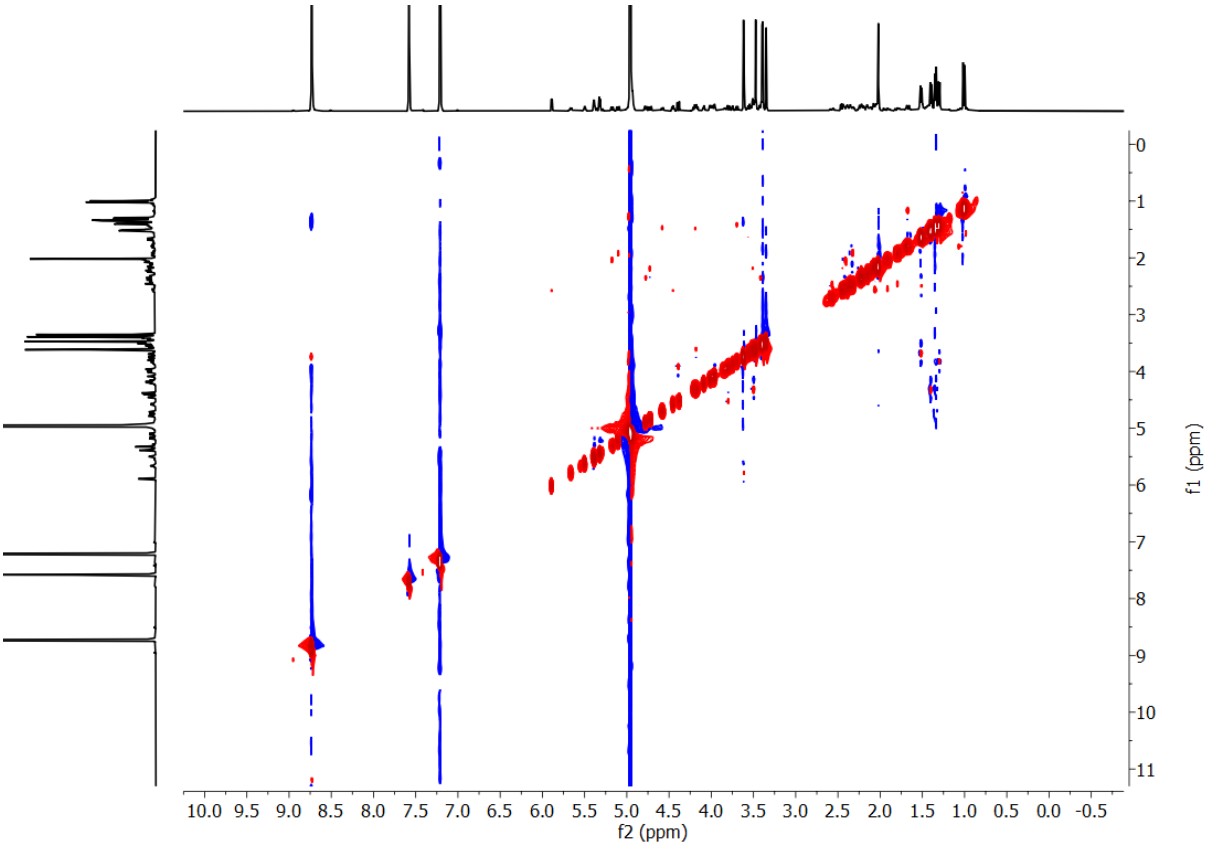

Figure S86. Effect of 1, 2, 3 and 5 on cell viability of stimulated J774 macrophages. Different concentrations of the compounds (50 - 200 μM) or gentian violet (GV; 10 μM, positive control) were added to the macrophage culture and incubated for 72 h. Control group (Ctrl) represents untreated cells stimulated with LPS (500 ng/mL) and IFN-γ (5 ng/mL). *Different from the control (Ctrl) group ($p < 0.0001$).

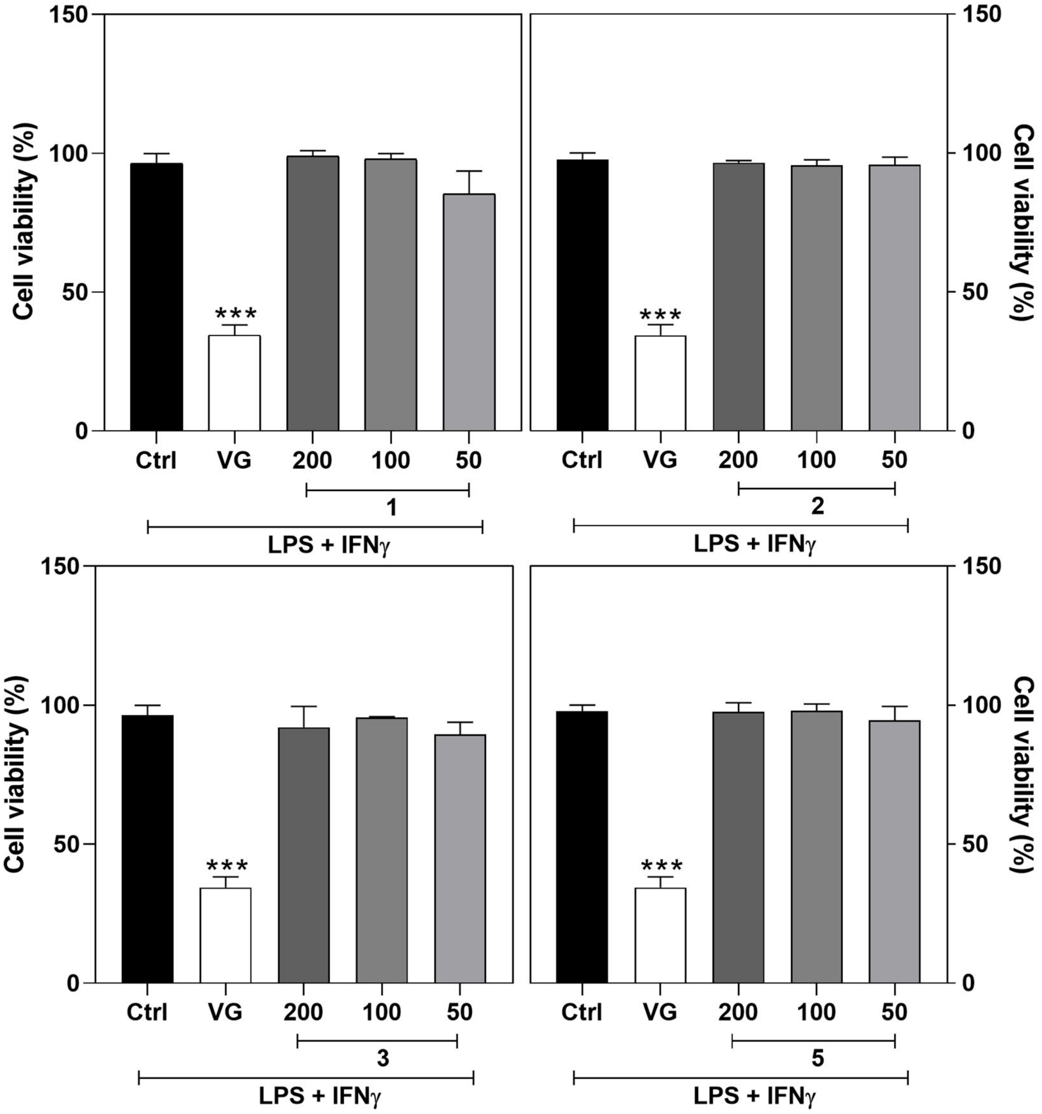

Supplement: Supplementary file 1 [file molecules-27-05992-s001.zip › molecules-1890134-supplementary.pdf]
